# Sequence determinants of human gene regulatory elements

**DOI:** 10.1038/s41588-021-01009-4

**Published:** 2022-02-21

**Authors:** Biswajyoti Sahu, Tuomo Hartonen, Päivi Pihlajamaa, Bei Wei, Kashyap Dave, Fangjie Zhu, Eevi Kaasinen, Katja Lidschreiber, Michael Lidschreiber, Carsten O. Daub, Patrick Cramer, Teemu Kivioja, Jussi Taipale

**Affiliations:** 1 Applied Tumor Genomics Research Program, Faculty of Medicine, University of Helsinki, Helsinki, Finland; 2 Medicum, Faculty of Medicine, University of Helsinki, Helsinki, Finland; 3 Department of Medical Biochemistry and Biophysics, Karolinska Institutet, Stockholm, Sweden; 4 Department of Genetics, Stanford University School of Medicine, Stanford, CA USA; 5 Department of Biochemistry, University of Cambridge, Cambridge, UK; 6 Department of Molecular Biology, Max Planck Institute for Biophysical Chemistry, Göttingen, Germany; 7 Department of Biosciences and Nutrition, Karolinska Institutet, Stockholm, Sweden; 8 Science for Life Laboratory, Stockholm, Sweden

**Keywords:** Systems biology, Computational biology and bioinformatics, Functional genomics, Gene regulation

## Abstract

DNA can determine where and when genes are expressed, but the full set of sequence determinants that control gene expression is unknown. Here, we measured the transcriptional activity of DNA sequences that represent an ~100 times larger sequence space than the human genome using massively parallel reporter assays (MPRAs). Machine learning models revealed that transcription factors (TFs) generally act in an additive manner with weak grammar and that most enhancers increase expression from a promoter by a mechanism that does not appear to involve specific TF–TF interactions. The enhancers themselves can be classified into three types: classical, closed chromatin and chromatin dependent. We also show that few TFs are strongly active in a cell, with most activities being similar between cell types. Individual TFs can have multiple gene regulatory activities, including chromatin opening and enhancing, promoting and determining transcription start site (TSS) activity, consistent with the view that the TF binding motif is the key atomic unit of gene expression.

## Abstract (web summary)

Analysis of massively parallel reporter assays measuring the transcriptional activity of DNA sequences indicates that most transcription factor (TF) activity is additive and does not rely on specific TF–TF interactions. Individual TFs can have different gene regulatory activities.

## Main

The temporal and spatial pattern of gene expression is encoded in the DNA sequence; this information is read and interpreted by TFs, which recognize and bind specific short DNA sequence motifs^1^. Major efforts have been undertaken to determine the DNA-binding specificities of TFs in vitro^2–5^ and map their binding positions in vivo^6,7^. TFs regulate gene expression by binding to distal enhancer elements and to promoters located close to the TSS^8,9^. Both enhancers and promoters are characterized by RNA transcription^10^, the presence of open chromatin^11^ and histone H3 lysine 27 acetylation (H3K27ac)^12^. In addition, promoters and enhancers are preferentially marked by histone H3 lysine 4 trimethylation and monomethylation^13^, respectively. Although these features can be mapped genome-wide in a high-throughput manner, they are correlative in nature and do not establish that an element can act as an enhancer, increasing expression from a promoter irrespective of position and orientation^8^. To more directly measure enhancer activity, MPRAs have been developed to study the activity of yeast^14^, *Drosophila*^15^ and human^16^ gene regulatory elements on a genome-wide scale. However, unbiased discovery of sequence determinants of human gene expression using only genomic sequences is made difficult by the fact that the genome is repetitive and has evolved to perform multiple functions. Furthermore, the human genome is too short to even encode all combinations, orientations and spacings of approximately 1,639 human TFs in multiple independent sequence contexts^1^. Thus, despite the vast amount of information generated by genome-scale experiments, most sequence determinants that drive the activity of human enhancers and promoters, and the interactions between them, remain unknown.

## Results

### Ultracomplex MPRAs with 100 times human genome coverage

**Fig. 1:**
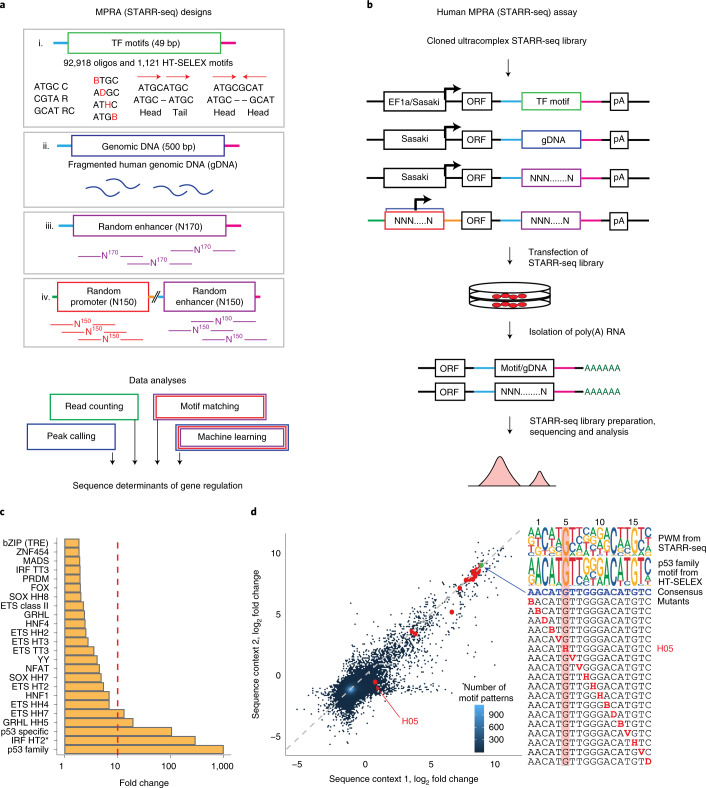
Few TFs display strong transcriptional activity in cells. **a**, Schematic representation of the MPRA (STARR-seq) libraries. For enhancer activity assays, a DNA library comprising synthetic TF motifs (i), human genomic fragments (ii) or completely random synthetic DNA oligonucleotides (iii) is cloned within the 3′ UTR of the reporter gene (open reading frame (ORF)) driven by a minimal δ1-crystallin gene (Sasaki) or EF1α promoter. For binary promoter–enhancer (iv) activity assays, random synthetic DNA sequences are cloned in place of the minimal promoter and in the 3′ UTR (Methods, Supplementary Note and Supplementary Tables 3 and 4). **b**, MPRA (STARR-seq) reporter construct and its variations, and the experimental workflow for measuring promoter or enhancer activity. The MPRA libraries are transfected into human cells, and RNA is isolated 24 h later, followed by enrichment of reporter-specific RNA, library preparation, sequencing and data analysis. The active promoters are recovered by mapping their transcribed enhancers to the input DNA and identifying the corresponding promoter. **c**, Enhancer activity of HT-SELEX motifs measured from the synthetic TF motif library in GP5d cells. Median fold change of the sequence patterns containing a single instance of the motif consensus or its reverse complement over the input library is shown. Red line marks 1% activity related to the strongest motif. Dimeric motifs are indicated by orientation with respect to core consensus sequence (GGAA for ETS, ACAA for SOX, AACCGG for GRHL and GAAA for IRF; HH, head to head; HT, head to tail; TT, tail to tail), followed by gap length between the core sequences. Asterisk indicates an A-rich sequence 5′ of the IRF HT2 dimer. Supplementary Table 5 describes the naming of the motifs in each figure. **d**, The effect of a mismatch on enhancer activity of the p53 family (p63) motif when a consensus base is substituted by any other base one position at a time. The log_2_ fold change compared to input is plotted for the same motif pattern in two different sequence contexts. The PWMs for HT-SELEX and STARR-seq motifs are shown; note that mutating G to any other base (H) at position 5 (H05) leads to almost complete loss of activity.

To systematically characterize the sequence determinants of human gene regulatory element activity, we developed a set of four MPRA libraries that cover more than 100 times the sequence space of the human genome (Fig. 1a and Methods). The libraries are based on the self-transcribing active regulatory region sequencing (STARR-seq) design^15^, in which putative enhancers are cloned downstream of a minimal promoter to the 3′ untranslated region (UTR) of a reporter gene. The constructs are transfected to cells, and the enhancer activity of the UTRs are then determined by RNA sequencing (RNA-seq) (Fig. 1b). Three libraries were designed to measure enhancer activities of combinations of known TF binding motifs embedded within two different 49-bp sequence contexts, ~500-bp fragments of genomic DNA and synthetic random 170-bp sequences; a fourth library was designed to measure both enhancer and promoter activities of synthetic random 150-bp sequences. Sequencing of the input libraries revealed their ultrahigh complexity, reaching billions of unique fragments (Supplementary Fig. 1a,b and Methods**)**.

### Few TFs display strong transcriptional activity in cells

**Extended Data Fig. 1:**
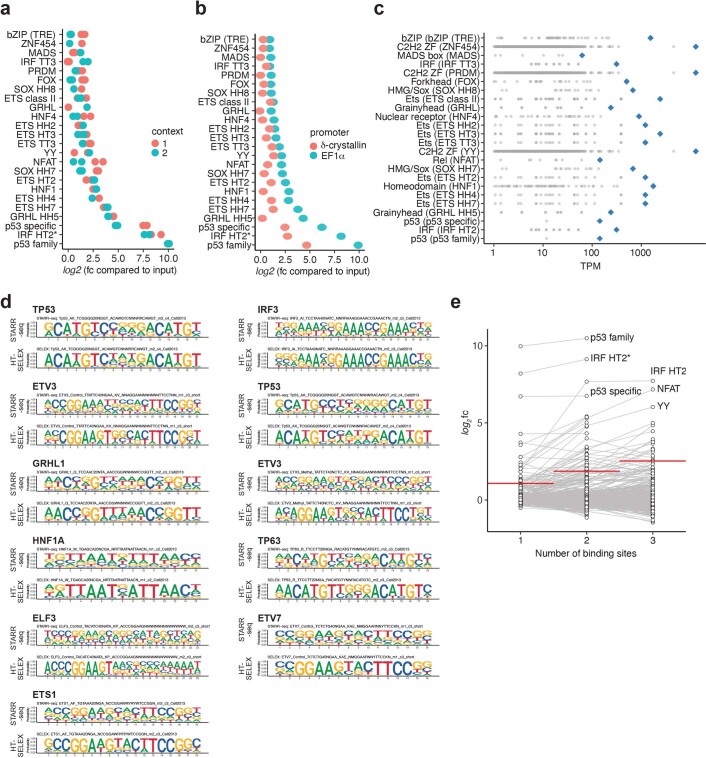
Enhancer activity from TF motifs using STARR-seq in GP5d cells. **a**, Enhancer activities of the HT-SELEX motifs in two different sequence contexts (color). For each motif, the log_2_ fold change of the consensus sequence and its reverse complement (if different) compared to input is shown in both contexts (Pearson R = 0.90; see Methods). Details of motif naming are described in legend to Fig. 1c. **b**, Enhancer activities of HT-SELEX motifs with two different CpG-free promoters, δ1-crystallin gene (Sasaki) or EF1α promoter (red and blue dots, respectively; median log_2_ fold change of the consensus sequence and its reverse complement in both contexts compared to input are shown for both promoters; Pearson R = 0.89). **c**, Expression of TF families that bind to the motifs with strong enhancer activity (see Fig. 1c) in GP5d cells. The DNA-binding domain (DBD) assignments for TFs from ref. ^1^ were used to assign the motifs to a set of DBDs. The blue diamond symbols show the total expression of the TF family (sum of tpm values from RNA-seq data; tpm = transcripts per million). Names for the DBD class and for the motif (in brackets) are shown. **d**, The motifs generated based on enhancer activities measured from motif STARR-seq experiments for sequences in which each of the consensus bases in a motif was substituted by N (*top*) compared to the corresponding SELEX motif (*bottom*). Eleven pairs are shown for which the activity PWM had information content ≥2 bits and for which the original SELEX motif was the best match with similarity *P* value <0.05 (motif similarity test of the TOMTOM program) when compared to all SELEX motifs used in the study. **e**, The effect of the number of binding sites on enhancer activity. For each motif, the fold change (log_2_) compared to the input (y axis) was estimated by taking the median fold change of all the sequences containing the given number of the motif consensus binding sites (x axis). The average fold changes for different numbers of binding sites (red lines) were calculated from the motifs that were detected with all copy numbers (n = 459).

To measure the enhancer activity of the known TF consensus sequences, we transfected GP5d colon carcinoma cells with the motif libraries (Fig. 1a, i) and purified total poly(A)^+^ RNA from the transfected cells. The synthetic motif sequences that were transcribed to RNA were recovered using reverse-transcription PCR, and the abundance of each sequence was then quantified by massively parallel sequencing (Methods). Comparison of the median activities of the individual TF consensus sequences revealed that several TFs had enhancer activity in GP5d cells (Fig. 1c, Extended Data Fig. 1a–c, Supplementary Note and Supplementary Table 5). The consensus sequence corresponding to the p53 protein family (p53, p63 and p73) displayed the strongest enhancer activity in this assay, suggesting that there is constitutive p53 activity in GP5d cells (Fig. 1c and Supplementary Fig. 1c–f). As the library contained each single-base substitution to the consensus sequences, we were able to generate activity position weight matrices (PWMs) for the motifs. For 11 motifs, the activity PWMs were highly similar to that of the motifs derived from an in vitro binding-specificity assay (high-throughput systematic evolution of ligands by exponential enrichment, HT-SELEX; Fig. 1d; Extended Data Fig. 1d), indicating that the measured enhancer activity originated from the TFs that bound to the motifs, demonstrating that the assay can be used to faithfully measure TF activities in cells.

**Fig. 2:**
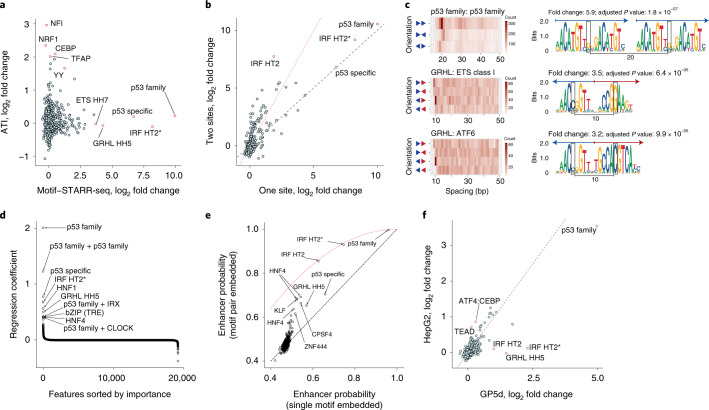
De novo enhancers display weak TF spacing and orientation preferences. **a**, Comparison of motif activities in biochemical binding (ATI assay; *y* axis) and STARR-seq (*x* axis) in GP5d cells (Pearson *R* = 0.032; see Fig. 1c for motif naming). **b**, Effect of number of motifs on enhancer activity from synthetic motif library in GP5d cells. For each motif, fold change (log_2_) compared to input is shown for one versus two sites. The black dashed line and the red dotted line represent the expected fold changes if two sites have the same effect as one and if two sites act in an additive manner, respectively. **c**, Spacing preferences for motif pairs analyzed from random enhancer experiment in GP5d cells. Heatmaps show counts of the motif pairs with the specific orientation (*row*) and spacing (*x* axis). The sequence logos show the most enriched spacing and orientation for each pair according to *P* value; the adjusted *P* value is calculated by comparing it to all others (one-sided Fisher’s exact test) and correcting for the total number of orientations and spacings tested for the pair (Methods and Supplementary Table 5). Blue and red arrows mark the first and second motifs of the pair and their orientations, respectively (unless they are the same). The distance between the information content centers of the motifs is marked. **d**, Regression coefficients for different TFs and TF pairs from logistic regression analysis of enhancer activities from random enhancer library in GP5d cells (Methods); features with the strongest predictive power are labeled. **e**, Nonlinear effect of multiple motifs in CNN trained on GP5d random enhancer STARR-seq data. A pair of the same motifs (indicated by labels) increases the predicted enhancer probability of the sequence above that expected from a single motif (dashed black line), but not above that expected from a model assuming independent binding to the two motifs (red dotted line). **f**, Comparison of enhancer activity of motifs measured from random enhancer library in GP5d and HepG2 cells (log_2_ fold change of motif match count over input in each cell line, Pearson *R* = 0.78; dashed line indicates identical activity between the cell lines).

Comparison of enhancer activities of motifs with the DNA-binding activities of respective TFs measured from the nuclear extract of GP5d cells by an active TF identification (ATI) assay^17^ (Methods) revealed that the transcriptional and DNA-binding activities were only weakly correlated (log_2_ fold change, Pearson *R* = 0.032; Fig. 2a and Supplementary Note). These results suggest that largely distinct sets of TFs display strong enhancer activity and strong DNA-binding activity in a cell.

### Synergy, additivity and saturation of activity

Apart from simple cellular alarm signals, most transcription is thought to require combinatorial action of many TFs^18–20^. Consistent with this, we observed that the average activity of all consensus sequences was very low, and for the majority of the TFs, the enhancer activity increased as a function of the number of consensus sequences (Extended Data Fig. 1e, red horizontal lines). Conversely, for the TFs that can activate transcription alone (e.g., p53 and IRF), two consensus sequences had lower activity than that predicted from an additive model (Fig. 2b, red dotted line), presumably due to saturation of both the occupancy and the downstream transcriptional activation. For TFs with intermediate activity levels (e.g., NFAT and YY), activity increased linearly rather than synergistically as a function of the number of binding sites (Extended Data Fig. 1e). The simplest model consistent with these observations is that human enhancer activation requires overcoming a repressive activity, after which activation is linear (additive) until it starts to saturate as it approaches a maximum level.

### Enhancers show weak TF spacing and orientation preferences

**Extended Data Fig. 2:**
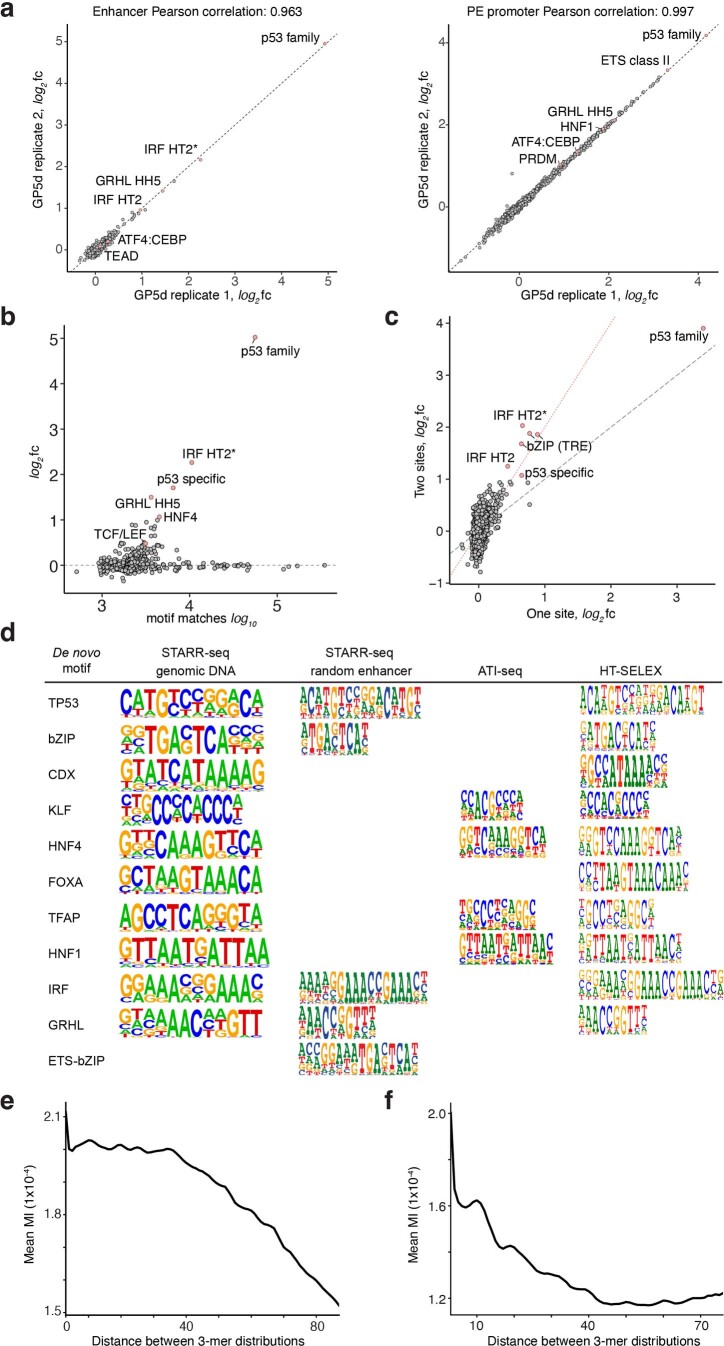
Features of enhancer activity measured from synthetic random DNA sequences in GP5d cells. **a**, Correlation of replicate experiments in GP5d cells. Motif-match log_2_ fold change values compared between two replicates from random enhancer experiment (*left*) and from promoters in binary STARR-seq experiment (*right*). See legend for Fig. 1c for details of motif naming. **b**, Motif matches enriched in the oligonucleotides showing the enhancer activity measured from random synthetic DNA (see Methods). **c**, Comparison of the effect of number of binding sites on enhancer activity from enhancer assay using random synthetic DNA. For each motif, the fold-change compared to the input is shown for one versus two sites. The matching was done separately for each strand so that the background probability of a match was 1 × 10^−5^. The black dashed line and the red dotted line represent the expected fold changes if two sites have the same effect as one and if two sites act independently of each other, respectively. **d**, *De novo* motif analysis for over-representation of TF motifs within DNA fragments enriched for enhancer activity in GP5d cells from genomic STARR-seq library, from synthetic random DNA library, and from the active transcription factor identification (ATI) assay. The motifs identified by HT-SELEX are shown for comparison. Only the motifs similar to those detected from genomic STARR-seq are highlighted here. Note the ETS-bZIP composite motif enriched in the random STARR-seq data. **e-f**, Mean of mutual information between 3-mer distributions as a function of distance separating the 3-mers in random enhancers (**e**) and binary STARR-seq enhancers (**f**). Pairwise dependency between 3-mer distributions varies with a period of approximately 10 bp and decays as a function of distance separating the k-mers, indicating that most of the dependencies in the enhancers are short-ranged.

To discover sequence features that contribute to human enhancer activity in an unbiased manner, we used extremely complex random enhancer library (Fig. 1a, iii) in GP5d cells. Motif mapping across replicate experiments indicated that motif activities were highly reproducible (Pearson *R* = 0.963; Extended Data Fig. 2a), displaying additivity and saturation similar to that observed with the motif library (Extended Data Fig. 2b,c). Enrichment of motifs corresponding to known TFs specific to colon cancer and intestinal lineage, such as TCF/LEF, GRHL and HNF4, was clearly observed (Extended Data Fig. 2b). De novo motif mining identified 22 TF motifs; most of these were for individual TFs or conventional heterodimers (Extended Data Fig. 2d and Supplementary Fig. 2a). One strong de novo ETS-bZIP composite motif was also identified, revealing a potential role for ETS-bZIP combinatorial control in colon cancer cells (Extended Data Fig. 2d). Analysis of spacing between motif matches identified few significantly overrepresented spacing preferences for motif pairs such as p53 family–p53 family, GRHL–ETS class I and GRHL–ATF6 (Fig. 2c); weak overall preference for motifs that were relatively close (<50 bp) was also observed (Extended Data Fig. 2e,f; Supplementary Note). These results suggest that TF grammar is strong at the level of heterodimers (analogous to ‘compound words’) but relatively weak at the level of specific combinations and spacing and orientation preferences between TFs (‘sentences’).

**Extended Data Fig. 3:**
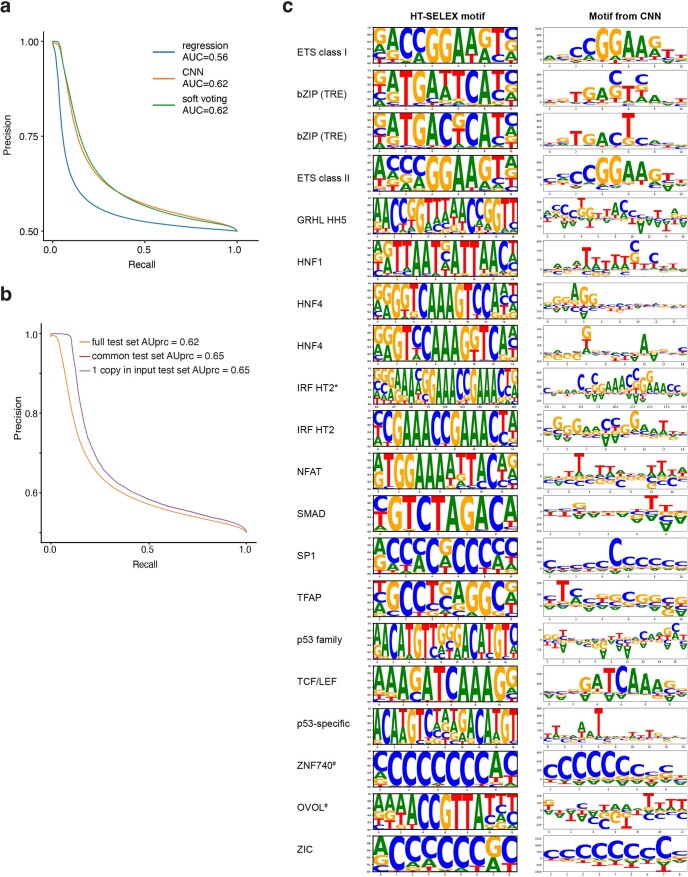
Analysis of random enhancer STARR-seq data using machine learning models. **a**, Binary classification accuracy of models trained to separate inactive (input) and active (STARR-seq) sequences from the random enhancer STARR-seq data in GP5d cells. CNN classifier (orange) outperforms a logistic regression model (blue) that uses HT-SELEX motifs (see Methods). Soft voting classifier (green) combining the predictions of the CNN and regression models does not improve over the CNN model. Classes are balanced in the test set so that a classifier assigning samples with random labels with equal probabilities would obtain an AUprc score of 0.5. **b**, Classification of high-confidence test set (class 1 sequences observed in both replicates of GP5d random enhancer STARR-seq experiment, red curve) with the GP5d random enhancer STARR-seq CNN classifier results in ~4% better AUprc value than classifying the full test set (yellow curve; see Methods), indicating that nonspecific carryover can explain only a small part of the relatively poor performance of the random enhancer STARR-seq classifiers. Removing the sequences that were sequenced more than once from the input library (~0.02% of all sequences, violet curve) does not further improve classification accuracy, indicating that sequences present in the highly complex input library in multiple copies do not affect the classification performance. Note that red and violet curves are overlapping. Classes are balanced in the test set as described for panel **a**. **c**, CNN activity contribution weight matrices (CACWM) learned by the CNN model from the random enhancer STARR-seq data analyzed using the ‘N-sweep’ approach. The N-sweep motifs generated using DeepLIFT^68^ and the random enhancer CNN model (*right*) for the 20 HT-SELEX motifs (*left*) with the largest absolute values for regression coefficient in the simple logistic regression model (see Methods for details, Supplementary Table 5 for SELEX motif patterns). Note that the contributions of the repressive motifs are negative towards predicting active enhancers and thus they appear below zero in the CACWM logos. Hash symbol marks the motifs classified as repressive by the logistic regression analysis. Parts of the HT-SELEX motif replicated in the CACWM indicate patterns learned by the CNN model.

To determine sequence features present in the de novo enhancers, we used machine learning classifiers. First, we determined the importance of known motif features using a logistic regression model (Methods); we found that only a handful of known TF binding motifs are needed for optimal classification, as only 26 out of 19,150 features had regression coefficient absolute values within 10% of the largest regression coefficient (Fig. 2d, Extended Data Fig. 3a and Methods). The most predictive features were motifs for known TFs important for tumorigenesis and colon development (Fig. 2d). These motifs were enriched, suggesting that the corresponding TFs act as transcriptional activators. The interactions between the motifs were largely additive, as specific pairwise combinations did not add substantially to the predictive power.

**Extended Data Fig. 4:**
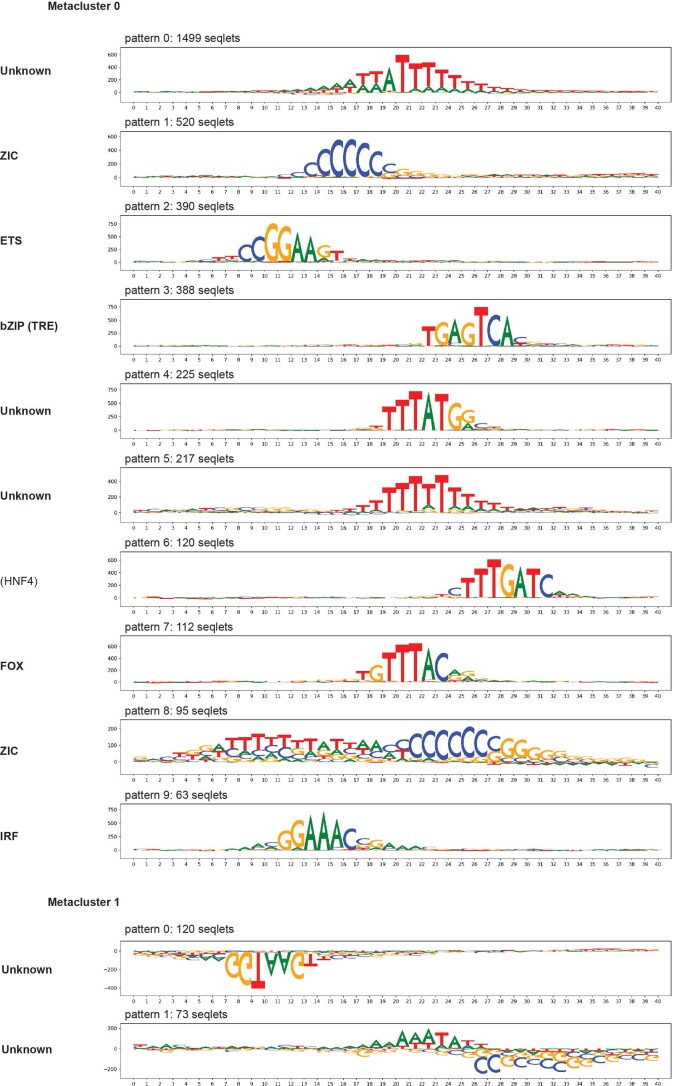
Motifs learned by CNN from random enhancer STARR-seq data analyzed using TF-MoDISco approach. Analysis of motifs learned by the CNN from random enhancer STARR-seq data using TF-MoDISco^69^ (transcription factor motif discovery from importance scores; see Methods). Patterns in metacluster 0 (*top*) and metacluster 1 (*bottom*) discovered by TF-MoDISco from unseen *in silico* generated random sequences classified as active enhancers by the CNN model using the in-built background model (see Methods for details). Number of seqlets supporting each pattern is shown for each pattern separately. Seqlets are segments of input that have substantial contribution to classification. Metacluster 0 from TF-MoDISco analysis contains patterns that increase enhancer probability according to the CNN model and metacluster 1 contains patterns that decrease enhancer probability. Motifs identified by TOMTOM are marked by bold typeface and the closest known motif by similarity is marked in parentheses.

Next, to identify possible novel sequence features that would allow more optimal classification, we trained a convolutional neural network (CNN)–based classifier similar to DeepBind^21^ on the sequence data. This method is capable of learning the sequence motifs, their combinations and their relative weights de novo. The CNN classifier performed substantially better than logistic regression using the same training, validation and test sets (11% increase in the area under the precision-recall curve (AUprc); Extended Data Fig. 3a,b and Methods). Analysis of the CNN classifier revealed that it had learned motif features similar to those identified by logistic regression (Fig. 2e, Extended Data Figs. 3c and 4, Supplementary Fig. 2 and Supplementary Note). In conclusion, these results indicate that individual TFs contribute to de novo enhancer function mostly without specific interactions between them.

### Only small number of TFs are specific for each cell type

**Fig. 3:**
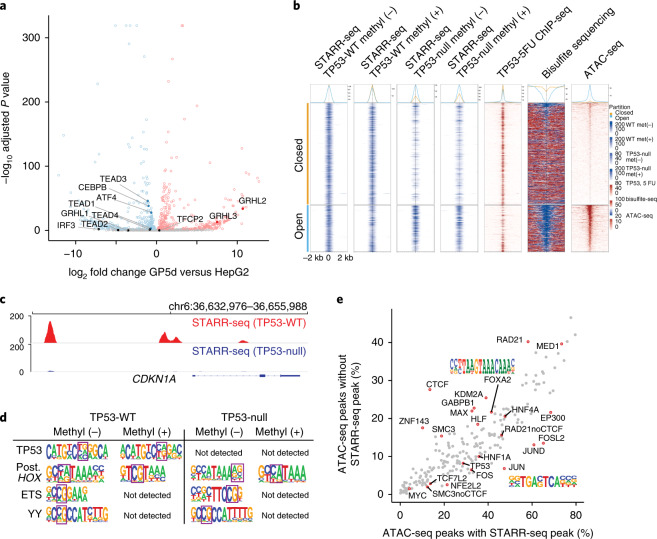
Cell type–specific gene expression and the effect of methylation on enhancer activity. **a**, Differential expression of genes encoding TFs (from Lambert et al.^1^) between GP5d and HepG2 cells. Red and blue dots represent the genes with higher expression in GP5d and HepG2 cells, respectively (multiple-testing adjusted *P* value < 0.01, Wald test; Methods). TFs with different motif activities between the cell lines are marked, including TEADs, GRHLs (and TFCP2, as it has motif similar to GRHLs), IRF3, ATF4 and CEBPB. **b**, Effect of CpG methylation on enhancer activity in *TP53*-null and wild-type (WT) GP5d cells. The regions around the summits of the top 1,000 genomic STARR-seq peaks in the unmethylated wild-type sample were classified as open (blue) or closed (orange) chromatin based on overlap with ATAC-seq peaks. For STARR-seq and ATAC-seq, average unique fragment coverage and read coverages are shown, respectively. For ChIP-seq and bisulfite sequencing, the average read coverage normalized to IgG and smoothed CpG methylation level for each window is shown, respectively. Top panel shows the average signal for each window in open (blue) and closed (orange) chromatin regions. 5FU, 5-fluorouracil (treatment to induce p53 binding to the genome); met, methyl. **c**, Genome browser snapshot showing the active enhancer peaks measured from the genomic STARR-seq library in GP5d cells. Loss of *TP53* results in the loss of STARR-seq peaks near the known p53 target gene *p21* (*CDKN1A*). **d**, De novo motif mining analysis for STARR-seq peaks from CG-methylated and unmethylated genomic DNA library in *TP53*-null and wild-type GP5d cells; CG is marked with a square box. Note that p53 motif is lost in *TP53*-null cells, motifs for many methylation-sensitive TFs (ETS and YY; see also Yin et al.^5^) are not detected after library methylation, and conversely, the posterior (*Post.*) homeodomain motif (italic) displays stronger CG dinucleotide after methylation. **e**, Comparison of ChIP-seq peaks within ATAC-seq peaks with (*x* axis) and without (*y* axis) STARR-seq peaks in HepG2 cells (Methods). The percentage of respective ATAC-seq peaks overlapping with at least one ChIP-seq peak is shown. For the cohesin subunits RAD21 and SMC3, the ATAC-seq peaks not overlapping a CTCF peak are marked separately (RAD21noCTCF and SMC3noCTCF).

To determine whether enhancers are cell-type specific, we used the random enhancer library (Fig. 1a, iii) to identify sequence features important for enhancer activity in HepG2 hepatocellular carcinoma cells. Comparison of enhancer motifs between the GP5d and HepG2 cells revealed that most motifs had similar enhancer activity across the cell lines (Pearson *R* = 0.78; Fig. 2f). The motifs with differential activity corresponded to lineage-determining TFs (GRHL in GP5d) and TFs important for tissue function (TEAD and ATF4:CEBPB in HepG2 cells). Importantly, the lineage-determining factors showed clear differential expression between the two cell types (Fig. 3a), indicating that activities of individual TFs are commonly affected by their expression level, although the overall correlation between motif activity and expression of corresponding TF family was weak (Extended Data Fig. 1a–c). These results show that the transcriptional landscape of a cell is dominated by cell-biological or ‘housekeeping’ TFs that show comparable activity across cell types and that the largest differences of motif activity between cell types are driven by TFs important for lineage specification.

### Genomic analysis reveals three types of active enhancers

**Fig. 4:**
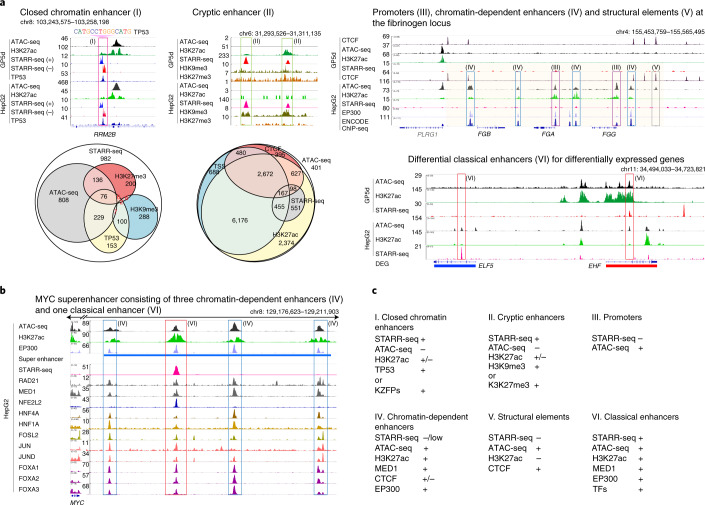
Genomic analysis reveals three types of transcriptionally active enhancers. **a**, Six types of regulatory elements classified on the basis of STARR-seq signal and chromatin features such as accessibility (ATAC-seq), TF binding and epigenetic modifications. Euler diagrams (bottom) show the overlap between genomic STARR-seq peaks and different genomic features (left) and between ATAC-seq peaks and different genomic features (middle) in HepG2 cells. Note that some of the small intersections are not shown (a full list of interactions is shown in Extended Data Fig. 6a). Genome browser snapshots showing examples of different types of regulatory features in HepG2 and GP5d cells are also shown. Colored boxes marked with roman numerals correspond to the different types of elements listed in panel **c**; clockwise from top: closed-chromatin enhancer (I) devoid of H3K27ac or ATAC-seq signal at TP53-target gene *RRM2B* (both the plus- and minus-strand STARR-seq signal is shown), cryptic enhancer (II) overlapping with repressive histone marks, promoters (III) and chromatin-dependent enhancers (IV) and structural CTCF element (V) at the fibrinogen locus (the ENCODE ChIP-seq track shows the number of overlapping TF peaks, with 206 TFs^7^ in total) and tissue-specific classical enhancers (VI) detected for *ELF5* (higher expression in HepG2, blue) and *EHF* (higher expression in GP5d, red). Note that the STARR-seq peaks are specific to the cell types where the adjacent gene is expressed. For ATAC-seq data, traces from the BAM coverage files are shown. DEG, differentially expressed gene. **b**, Chromatin-dependent enhancers and classical enhancers combine to form superenhancers. Genome browser snapshot of a *MYC* superenhancer in HepG2 cells marked by a STARR-seq peak overlapping with the binding site for TF with strong transactivation activity (NFE2L2) converging on equidistant chromatin enhancers bound by cohesin, Mediator, forkhead and other liver-specific TFs. **c**, Summary of the features that define the six genomic element types.

**Extended Data Fig. 5:**
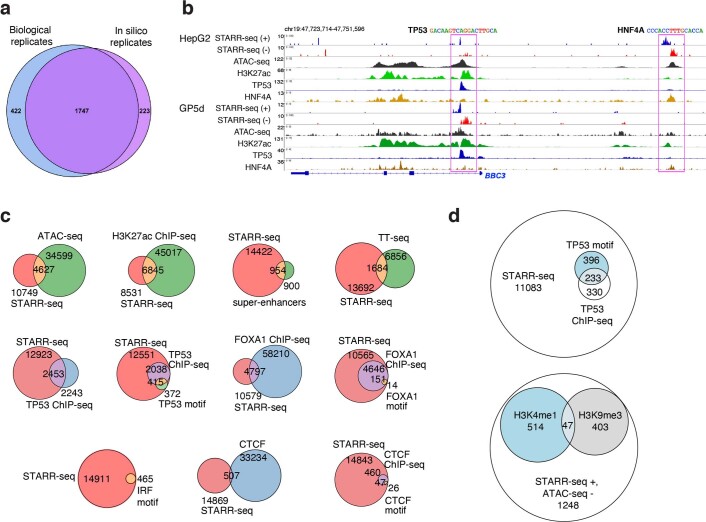
Features of enhancer activity measured from human genomic DNA. **a**, Venn diagram showing the concordance of biological and *in silico* replicate IDR peaks from genomic STARR-seq in HepG2 cells, demonstrating that the IDR method yields similar peak-calls (~90% specificity if biological replicate analysis is considered ground truth) when it is used as an internal control approach (see Methods). **b**, Genome browser snapshot of enhancer activity and TF binding at the *BBC3* gene locus in GP5d and HepG2 cells, demonstrating the excellent signal-to-noise ratio of the STARR-seq data. Both plus and minus-strand STARR-seq signal is shown, as well as ATAC-seq coverage and ChIP-seq for H3K27ac, TP53, and HNF4A. Cell type–specific STARR-seq signals agree with tissue-specific TF binding with a common TP53-bound enhancer and a HepG2-sepcific HNF4A-bound enhancer highlighted with pink boxes. Genomic sequences at these loci with TP53 and HNF4A motifs are shown. **c**, Overlap between genomic STARR-seq peaks and genomic features (regulatory element features, ChIP-seq peaks for individual DNA-binding proteins and their motifs) in GP5d cells (see Methods). The total number of peaks for each experiment is the sum of the values inside its circle. The significance of the overlaps between STARR-seq and the other measurements (two-tailed Fisher’s exact test, see Methods for details): ATAC-seq: *P* < 2.2251×10^−308^; H3K27ac ChIP-seq: *P* < 2.2251×10^−308^; superenhancers: *P* < 2.2251×10^−308^; TT-seq enhancers: *P* < 2.2251×10^−308^; TP53 ChIP-seq: *P* < 2.2251×10^−308^; CTCF ChIP-seq: *P* = 4.1888×10^−111^; FOXA1 ChIP-seq: *P* < 2.2251×10^−308^. **d**, *Top*, overlap of genomic STARR^−^seq peaks with TP53 ChIP-seq peaks and the p53 motif in HepG2 cells. The motif-match overlap was calculated using 24,176 highest affinity matches in the genome (all with score > 9). *Bottom*, overlap between STARR^+^, ATAC^-^ peaks and ChIP-seq data for H3K4me1 and H3K9me3 histone modifications in HepG2 cells. Collectively, the results shown in panels **c** and **d** demonstrate that although p53 and IRF motifs are the strongest activators in the cells (see Fig. 1c, Fig. 2f), they contribute to a relatively small proportion of the overall enhancer activity, based on the overlap analysis of genomic STARR-seq peaks with TP53 ChIP-seq peaks and with p53 and IRF3 motifs (see also Supplementary Note).

**Extended Data Fig. 6:**
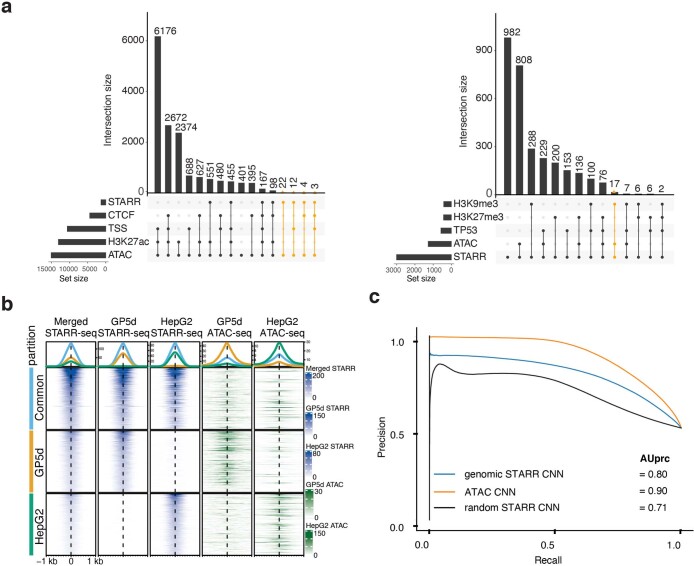
Overlap analysis of human genomic STARR-seq and ATAC-seq data. **a**, Overlap between ATAC-seq peaks (*left*) and STARR-seq peaks (*right*) with different genomic features in HepG2 cells. Full lists of interactions related to Euler plots in Fig. 4a are shown here, and the ones not shown in the Euler plots are highlighted with orange color. The horizontal bars show the total number of each type of feature overlapping the top quartile of the ATAC-seq peaks (15125 peaks; *panel on the left*) or STARR-seq peaks (3010 peaks; *panel on the right*) according to the maximum fragment coverage. The vertical bars show the size of the intersection indicated by the circles in the matrix. **b**, Comparison of active genomic regions in GP5d and HepG2 cells. Color scales indicate STARR-seq and ATAC-seq fragment coverages for three groups of peaks: STARR-seq peak in both cell lines (Common), and only in GP5d or in HepG2 cells. From each group, the top 1000 highest ranking peak regions (±1 kb of the summit) according to fold-change over control are shown (sum of ranks used for common peaks). The ‘Merged STARR-seq’ column shows the sum of fragment coverages from the two cell lines. The tracks are centered according to individual peak summits, or to the middle point of the two summits (for common peaks). **c**, Binary classification between ATAC-seq peaks and randomly sampled background from the genome (see Methods) using different CNN classifiers trained on GP5d ATAC-seq peaks (orange), sequences from the GP5d genomic STARR-seq experiment (blue), or sequences from the GP5d random STARR-seq experiment (black). Note that the classifier performs well even when trained using genomic STARR-seq data, suggesting that the sequence features in the two types of elements are similar. Classes are balanced in the test set, meaning that a classifier assigning samples with random labels with equal probabilities would obtain an AUprc (area under precision-recall curve) score of 0.5.

To determine how sequence features combine to generate functional genomic enhancers, we assayed genomic enhancer activity in GP5d and HepG2 cells at ~1.5-bp resolution (Fig. 1a, ii, Supplementary Fig. 1a,b, Extended Data Fig. 5a,b and Methods) before and after methylation of the library (Fig. 3b). To determine the role of TP53 in enhancer activity, we performed similar experiments in *TP53*^−/−^ GP5d cells. The signal was highly specific, as indicated by the fact that loss of TP53 resulted in loss of most enhancer peaks containing its motif (Fig. 3c,d). However, despite being the strongest activator in both cell types (see Fig. 2f), TP53 contributed to a relatively small proportion of the overall enhancer activity in both GP5d and HepG2 cells; only 16% and 4.9% of the genomic STARR-seq peaks overlapped with TP53 chromatin immunoprecipitation sequencing (ChIP-seq) peaks (Extended Data Fig. 5c,d and Supplementary Note). Analysis of the methylated libraries revealed that activities of methylated genomic elements were consistent with the known effect of methylation on TF DNA binding (Fig. 3d and Yin et al.^5^). Consistent with the known association between accessible chromatin, TF binding and enhancer activity, the STARR-seq peaks overlapped significantly with chromatin accessibility; specifically, 30% of the STARR-seq peaks in GP5d and 27% in HepG2 cells overlap with assay for transposase-accessible chromatin using sequencing (ATAC-seq) peaks in the same cell types (Figs. 3e and 4a–c and Extended Data Figs. 5c and 6a,b). Furthermore, ATAC-seq peaks could be predicted by a CNN trained using genomic or random synthetic STARR-seq sequences (AUprc 0.80 and 0.71, respectively; Extended Data Fig. 6c), indicating that the sequence features discovered using STARR-seq correspond partially to the features that are associated with open chromatin in vivo.

We next used the differential signals for chromatin accessibility (ATAC^+^ or ATAC^−^) and classical enhancer activity (STARR^+^ or STARR^−^) for defining different classes of gene regulatory elements along with ChIP-seq data for individual TFs; the histone marks H3K27ac, H3K9me3 and H3K27me3; and the structural chromatin protein CTCF (Fig. 4a,c). This analysis revealed six classes of elements: (1) closed-chromatin enhancers (STARR^+^ and ATAC^−^), (2) cryptic enhancers (silenced STARR^+^ and ATAC^−^ regions), (3) promoters (ATAC^+^ and STARR^+/−^), (4) chromatin-dependent enhancers (STARR^−/low^ and ATAC^+^ with active histone mark H3K27ac), (5) structural chromatin elements (STARR^−^, ATAC^+^ and CTCF^+^) and (6) classical enhancers (STARR^+^ and ATAC^+^).

**Extended Data Fig. 7:**
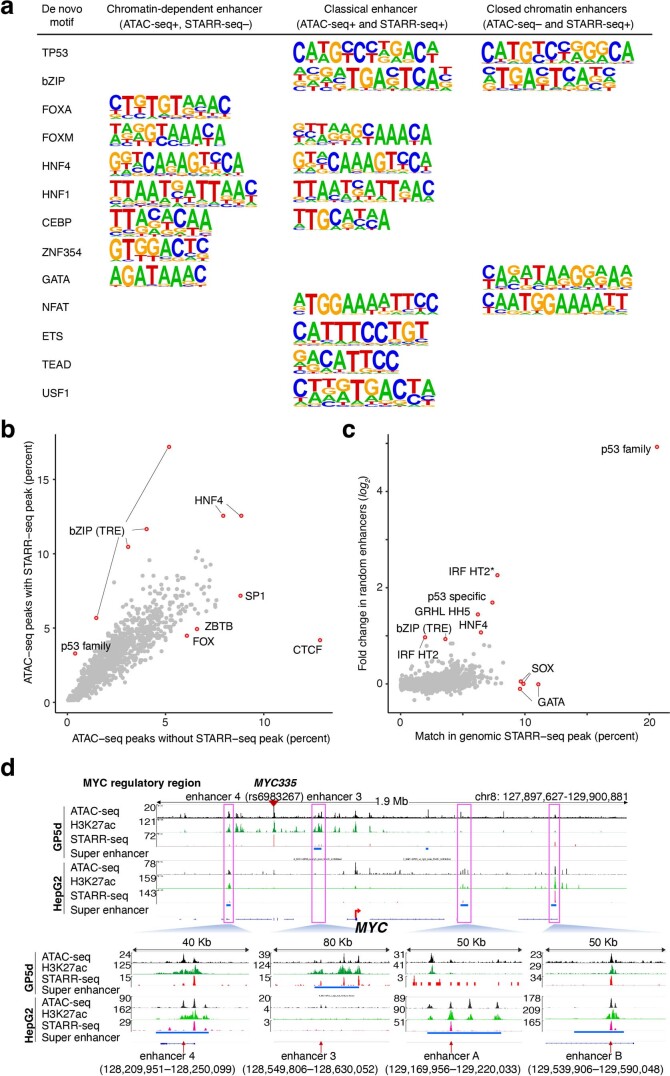
Comparison of regulatory features of different enhancer classes. **a**, *De novo* motif analysis for over-representation of TF motifs within different enhancer classes in HepG2 cells defined on the basis of genomic STARR-seq and ATAC-seq signals (intersections as in Fig. 4a and Extended Data Fig. 6a, but the TSS-overlapping sequences have been excluded from the analysis, see Methods). **b**, Motif matches enriched in the chromatin-dependent enhancers (STARR-seq-, ATAC-seq+) and the classical enhancers (STARR-seq+, ATAC-seq + ) in HepG2 cells; intersections as in Fig. 4a and Extended Data Fig. 6a, but the TSS-overlapping sequences have been excluded from the analysis, see Methods). **c**, Motif matches enriched in the oligonucleotides showing the enhancer activity measured from the synthetic random enhancer library (y axis) compared to the genomic STARR-seq peaks (x axis) in GP5d cells. For each motif, the overlaps of the 100,000 highest affinity matches in the genome (or all with a score at least 9 if no 100,000 such matches) with the STARR-seq *in silico* IDR peaks (3250; see Methods for details) were included. Dimeric motifs are indicated by orientation with respect to core consensus sequence as described in legend to Fig. 1c. Asterisk indicates an A rich sequence 5’ of the IRF HT2 dimer. **d**, Genome browser snapshot of the regulatory region of the MYC gene showing STARR-seq signal from GP5d and HepG2 cells along with ATAC-seq, and ChIP-seq for H3K27ac. Superenhancers for HepG2 are from http://www.licpathway.net/sedb and for GP5d analyzed as described in Methods. Enhancers 3 and 4 have been previously described in ref. ^70^.

Analysis of the methylated genomic elements revealed that the cryptic enhancers were not silenced by methylation (Fig. 3b). Instead, they were inactive due to the presence of either H3K27me3 (polycomb; ‘poised’ enhancer^22^) or H3K9me3/HP1 repressive chromatin marks. The three other types of enhancers (closed chromatin, chromatin dependent and classical) appeared active based on the fact that inclusion of the corresponding features improved prediction of differential gene expression between GP5d and HepG2 cells (Supplementary Table 6 and Methods). Analysis of ChIP-seq peaks and motifs present in the different classes of elements revealed that classical and closed-chromatin enhancers bound to TFs and contained motifs that were similar to those that were found in active elements selected from random sequences (Extended Data Fig. 7a and Fig. 2f). Classical enhancers were preferentially bound by TFs with strong activator domains (e.g., FOS and JUN), whereas chromatin-dependent enhancers displayed relatively weak preference for HLF and FOXA motifs, and both types of enhancers were bound by HNF4A (Fig. 3e and Extended Data Fig. 7b). These results indicate that cells contain three distinct classes of enhancers (Supplementary Note): (1) classical enhancers^8^ that overlap with open chromatin and transactivate a heterologous promoter regardless of position or orientation; (2) chromatin-dependent enhancers that cannot be effectively detected using STARR-seq (see also Inoue et al. ^23^) and have strong signal for open chromatin and the activating histone mark H3K27ac; and (3) closed-chromatin enhancers whose detection requires STARR-seq, as these elements are not strongly enriched for chromatin marks associated with enhancer activity.

Consistent with few TFs determining the overall transcriptional landscape of a cell, the genomic STARR-seq peaks were enriched for relatively few motifs (Extended Data Fig. 2d). The motifs themselves were similar to known monomeric, dimeric and composite TF motifs determined using HT-SELEX^4^ and consecutive affinity-purification systematic evolution of ligands by exponential enrichment (CAP-SELEX)^24^ (Extended Data Fig. 2d). The motifs discovered from genomic and random enhancers were also largely similar (Extended Data Fig. 2d). The main difference was the enrichment of pioneer factor motifs such as GATA and SOX in genomic fragments (Extended Data Fig. 7c); these motifs may be specifically associated with classical genomic enhancers because of the ability of the corresponding TFs to displace nucleosomes and/or open higher-order chromatin. Many discovered genomic STARR-seq motifs also displayed strong DNA-binding activity in an ATI assay (Extended Data Fig. 2d), indicating that strong DNA binders are important for in vivo enhancer activity, potentially because they are capable of opening chromatin^17^. In summary, the sequence features of classical genomic enhancers are highly similar to those enriched from random sequence; these motifs define the classical enhancer activity of a cell. In addition to this activity, additional chromatin-dependent enhancers confer tissue specificity to genes; these elements are characterized by motifs for TFs that have lower transactivation activity, suggesting that these TFs act via chromatin to facilitate the activity of promoters and associated classical enhancers. Consistent with this view, the strongest cellular enhancers, superenhancers, typically consist of arrays of chromatin-dependent elements associated with a classical enhancer (Fig. 4b and Extended Data Fig. 7d).

### Sequence features of de novo promoters and enhancers

**Fig. 5:**
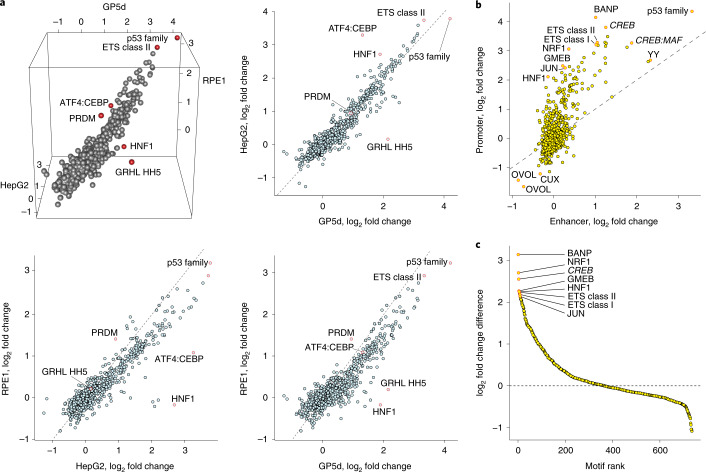
Comparison of sequence features of de novo enriched human promoters and enhancers. **a**, Plot showing the enrichment of TF motif matches in promoters selected from completely random sequences across three mammalian cell lines: GP5d colon cancer, HepG2 liver cancer and RPE1 retinal pigmented epithelial cells (dashed line marks identical activity; Pearson correlation values for log_2_ motif-match fold changes compared to input for GP5d versus HepG2 = 0.95, HepG2 versus RPE1 = 0.92 and GP5d versus RPE1 = 0.91). Dimeric motifs are indicated by orientation with respect to core consensus sequence as described in legend to Fig. 1c. **b**, Comparison between enrichment of motif matches at enhancers (*x* axis) versus promoters (*y* axis) in GP5d cells (active sequences selected from synthetic random promoter and enhancer sequences). The motifs marked with italic typeface are de novo motifs mined from the GP5d TSS-aligned sequences. **c**, Motifs that enrich specifically in the promoter position, as measured by a difference in log_2_ fold change. The motifs that are enriched the most are indicated by red circles and labeled. Motifs with negative difference in log_2_ fold change (below dotted line) are repressive and decrease promoter activity; no motif specifically enriches at enhancers (**b**).

To identify sequence determinants of human promoter activity, we assayed the activity of the binary STARR-seq library consisting of random sequences placed in the position of both the promoter and the enhancer (Fig. 1a, iv). For this analysis, we used two tumor cell lines (GP5d and HepG2; endodermal origin) and an untransformed cell line derived from retinal pigment epithelium (RPE1; ectodermal origin). Robust promoter activity was observed in all three cell lines from a subset of the random sequences, and motif mapping across replicate experiments in GP5d cells showed that motif activities were highly reproducible (Pearson *R* = 0.997; Extended Data Fig. 2a). As observed for the motifs at active enhancers, most motifs enriched at promoters were similar in all cell types (Fig. 5a). The motifs that displayed differential activity were linked to lineage determination (e.g., HNF1A) and specialized cell functions (ATF4:CEBP in HepG2 cells; Fig. 5a). Comparison of the active sequences in GP5d cells revealed that many sequence motifs were enriched in both the promoter and enhancer positions (Fig. 5b). However, some specificity in the enrichment was also observed. For example, although p53 and YY motifs were similarly enriched at promoters and enhancers, ETS (promoters vs. enhancers 9.5 versus 2.1 in linear scale) and recently discovered BANP^25^ motifs (17.7 versus 2.0) were preferentially, and NRF1 (8.4 versus 1.3) as well as HNF1 motifs (4.3 versus 0.9) almost exclusively enriched at promoters (Fig. 5b,c). No motif enriched only at enhancers, indicating that all motifs with enhancing activity can also act from a proximal position at the promoter (Fig. 5b). Of note, some negative effects were also observed (Fig. 5b), consistent with previously known repressive functions of the corresponding TFs (e.g., OVO-like transcriptional repressor 1 and cut-like homeobox). In summary, these results indicate that human promoters can be enriched from random sequences and that active promoter elements are highly similar among different cell types.

### A G-rich element that interacts with the TSS

**Fig. 6:**
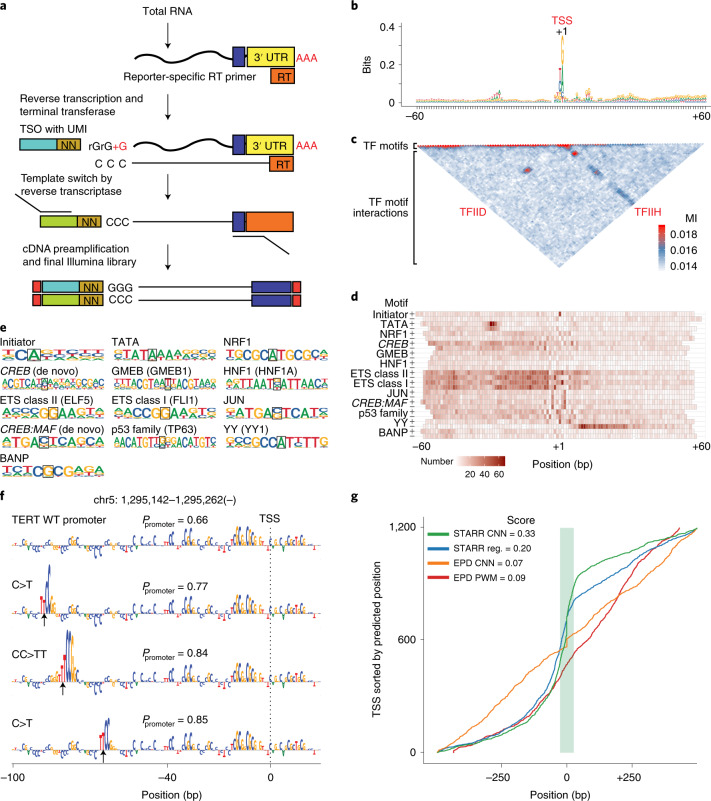
Analysis of positional specificity of sequence elements defining human promoters. **a**, Template-switch strategy for capturing the 5′ sequence of the transcripts to determine the TSS location within the promoters enriched from random sequences (reporter-specific primer (orange), template-switch oligo (TSO) containing a unique molecular identifier (UMI) (brown), sequencing adapters (turquoise/green, blue) and Illumina linkers (red)). The template-switch data are used in **b**–**d** (Supplementary Note and Supplementary Table 7). **b**, Sequence logo constructed from 17,235 active GP5d promoter sequences from the binary STARR-seq experiment aligned based on the measured position of their TSS (+1). **c**, Mutual information (MI) plot from the same set of active promoters used in **b**. Most mutual information is observed close to the diagonal (indicating the presence of TF motifs), but two longer-range interactions are observed between the TATA box and TSS and between the TSS and a G-rich element 3ʹ of it. **d**, Heatmap showing positional preferences for classical TSS-associated motifs (initiator, TATA; Methods), the most highly enriched motifs at promoters compared to enhancers (Fig. 5b) and generally highly enriched motifs (p53 family, YY and *CREB:MAF*). Heatmap color indicates the number of motif matches in one strand (the background probability of a match 5 × 10^−4^); italic typeface marks de novo motifs mined from the GP5d TSS-aligned sequences (also in panel **e**). **e**, Sequence logos of the motifs shown in the heatmap of panel **d**. The information content center column used to position the matches in the heatmap is highlighted. **f**, Predicted sequence determinants at the *TERT* promoter from DeepLIFT analysis (Methods) of the CNN (top) and the effect of three cancer-associated driver mutations^29^ (arrows) on its predicted promoter probability (*P*_promoter_). **g**, Cumulative distance between predicted and annotated TSS positions shown against a test set of GP5d genomic TSSs (~1,200 sequences) for CNN trained on human genomic TSS data (orange) and promoters enriched from random sequences (green), a PWM-based model (red) and a regression (reg.) model using positional match data (blue). Genomic TSS positions are aligned at 0; the score indicates the fraction of predicted TSS positions within ±25 bp from the annotated TSS (green area).

To evaluate the positioning of the different features relative to the TSS, we first determined the TSS position within the promoters derived from random sequences by recovering the 5′ end of the transcript using a template switch (Fig. 6a), yielding 85,217 unique TSS positions. Alignment of the recovered sequences with respect to the TSS positions (Methods) revealed a relatively high information content feature located at the TSS that corresponded to the classic initiator motif (Fig. 6b). In addition, a clear AT-rich region was observed at the canonical −30 position of the TATA box. However, we did not detect other TSS-proximal motifs that have previously been described (BRE, DPE, MTE, DCE, X-core promoter element and TCT^20,26^). The transcript side was characterized by a modest increase in G across a relatively wide region (+10 to +35); this feature is also observed in genomic promoters (Supplementary Fig. 3). To identify interactions between the features, we performed mutual information analysis (Methods). The strongest signal was for short-range interactions located 5ʹ to the TSS, excluding a region just upstream of the TATA box; this signal represents enrichment of individual TF motifs. Two mutually exclusive longer-range interactions were detected: one between the TATA box and the TSS and the other between the TSS and the G-rich downstream sequence (Fig. 6c). This pattern is consistent with the loading of the RNA polymerase II either ‘heel first’ (TFIID) or ‘toe first’ (TFIIH) with respect to the TSS.

Motif mapping revealed that many TF motifs were also specifically positioned and oriented relative to the TSS (Fig. 6d,e). The strongest positional signals were observed for the TATA box, initiator and YY (YY1). YY1 motifs were mainly enriched on the transcript side (the first C of the CCAT sequence occurring on the minus strand at position +12), oriented in a manner that the YY1 protein can position and orient the RNA polymerase II to direct transcription toward the YY motif (Fig. 6d and Houbaviy et al.^27^). In addition, many TF motifs preferentially enriched close to the TSS (Fig. 6d). On the 5ʹ side, the strongest enrichment occurs close to the TSS, slowly decreasing toward the TATA box; preferential enrichment upstream of the TATA box was also observed for some TFs (e.g., BANP^25^). On the 3ʹ side, the enrichment declines more sharply with very little motif enrichment observed beyond the +20 position from the TSS (Fig. 6d,e). In summary, these results highlight that some, but not all, TFs have positional dependency related to the TSS.

### Predicting transcriptional activity from sequence features

**Extended Data Fig. 8:**
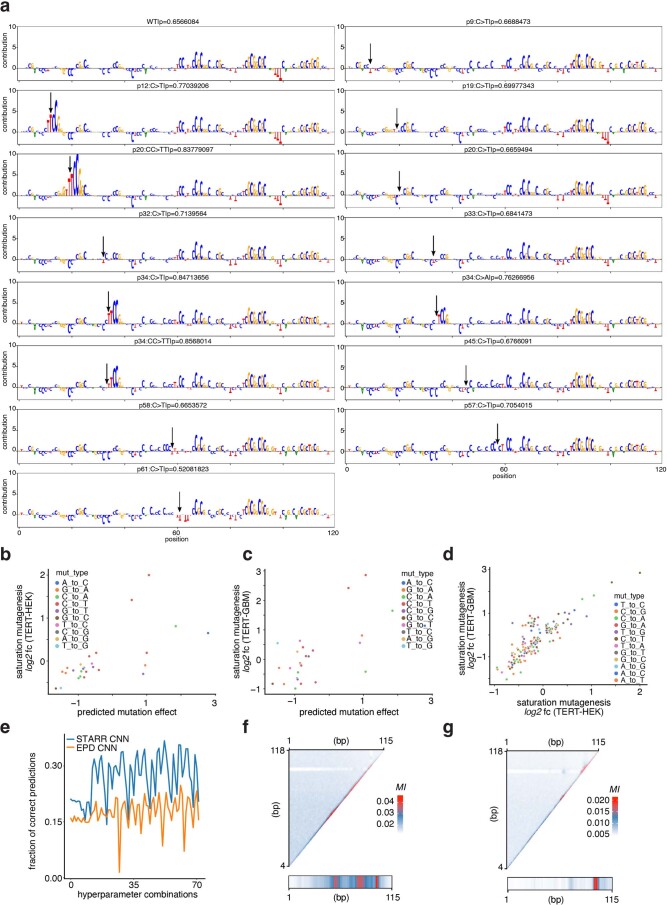
STARR-seq promoter CNN model for scoring *TERT* promoter mutants and analyzing promoter features. **a**, All recurring *TERT* promoter mutants from ref. ^29^ scored with the STARR-seq promoter CNN model and visualized with DeepLIFT^68^ (see Methods). The predicted promoter probability and location, and the type of the mutation are shown above each sequence (TSS at position 100). **b–d**, *TERT* promoter point mutations predicted by the CNN model trained using active promoters enriched from random sequences in GP5d cells (logarithm of odds ratio between the predicted promoter probability of the mutated vs. the wild-type sequence, see Methods) correlated to the measured effect of the same mutations in a saturation mutagenesis MPRA study (data for HEK293T and SF7996 cells from ref. ^28^): predicted effect vs. measurements in HEK293T (**b**; Spearman R = 0.737, two-sided *P* = 1.768×10^−5^) and vs. SF7996 cells (**c**; Spearman R = 0.604, two-sided *P* = 8.455×10^−4^). Cross-correlation between HEK293T and SF7996 cells (**d**; Spearman R = 0.785, two-sided *P* = 1.674×10^−36^). In each panel, only mutations with *P* < 0.05 in both of the correlated predictions/measurements are shown (see Methods for details). **e**, The CNN trained on promoter data from binary STARR-seq experiment (blue) outperforms the CNN trained on the Eukaryotic Promoter Database (EPD, orange) on all but one of the 72 hyperparameter combinations tested (paired Student’s t test, two-sided *P* = 9.68 × 10^−23^) in predicting the TSS position on unseen genomic test data. The fraction of predicted TSS positions within ±25 bp of the annotated TSS positions (y axis) vs. hyperparameter combinations (x axis; see Supplementary Table 8) is shown. **f, g**, Mutual information (MI)-based comparison of pairwise interactions learned by the CNN models trained on the STARR-seq active promoters (**f**) and the human genomic promoters (**g**). The triangle-shaped upper panels show the MI values between 3-mer distributions at each position of the models (see ref. ^65^). Below is a zoomed-in view of the diagonal of the MI matrix showing the positional enrichment of TF binding sites. For both models, random sequences with predicted promoter probability over 0.9 according to 10 best individual CNNs were used for the MI analysis (see Methods and Supplementary Note for details).

To determine how well transcription can be predicted based on the de novo promoter sequences, we trained a CNN model (Methods) to predict the TSS positions genome-wide. To test the CNN, we first used it to score wild-type and mutant forms of the TERT promoter^28,29^ (Methods); the model correctly predicted that known cancer-associated mutations^29^ increase the activity of this promoter (Fig. 6f and Extended Data Fig. 8a–d). We next used the CNN to predict the positions of active TSSs in GP5d cells using TSS annotation derived from the Eukaryotic Promoter Database (EPD)^30^, and the activity of the TSSs was determined using cap analysis of gene expression (CAGE; Methods). This analysis revealed that promoters enriched from random sequences were more predictive than the genomic sequences themselves; 33% of the positions of unseen genomic TSSs were accurately predicted by the CNN trained on the promoters enriched from random sequences, as opposed to 7% predicted by the CNN trained on the EPD promoters (Fig. 6g and Extended Data Fig. 8e). A mutual information–based analysis of interactions learned by the CNN classifiers (Methods) revealed that the classifiers trained on STARR-seq data learned a stronger position-specific signal than the classifiers trained on the EPD data, which relied more on information present at a relatively short region around the TSS (Extended Data Fig. 8f,g and Methods). These results highlight the power of unbiased interrogation of sequence space that is 100 times larger than that of the human genome.

### Enhancer–promoter interactions are additive

**Fig. 7:**
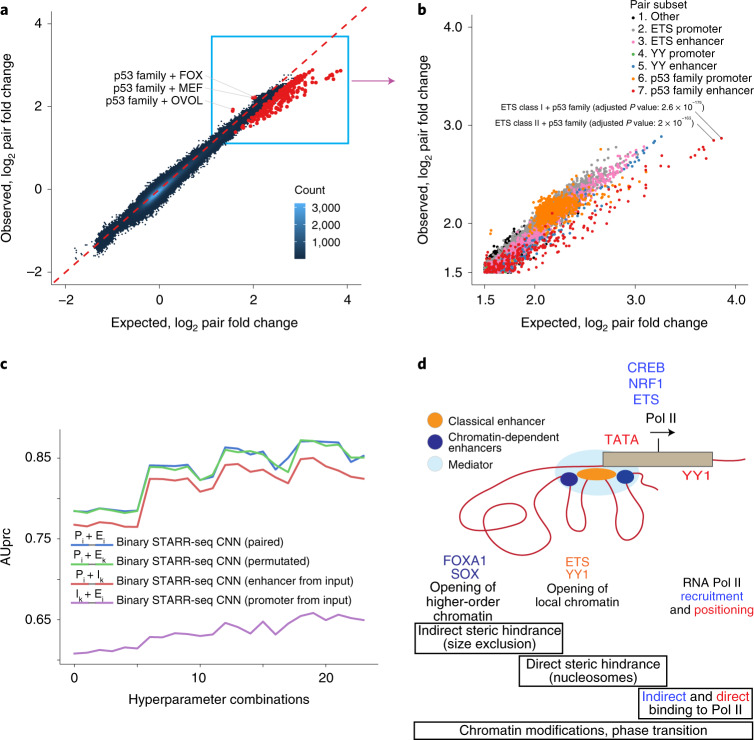
Enhancer–promoter interactions are additive and nonspecific in nature. **a**, Activity of promoter–enhancer pairs detected from the binary STARR-seq experiment; the observed log_2_ fold change of each pair compared to input DNA (*y* axis) against the expected change (*x* axis), assuming that the promoter and enhancer motifs act independently of each other (with a background probability of a motif match as 5 × 10^−5^; Methods). Significant interactions (multiple hypothesis-corrected *P* value <0.05; two-sided binomial test; Methods) are marked red, and all pairs having significant positive interaction are named (promoter motif + enhancer motif). Red dashed line shows the observed number exactly matching the expected one. **b**, Magnified upper right-hand corner of panel **a**. The pairs with the lowest *P* values are marked. **c**, AUprc for four CNN classifiers with identical architectures trained on different datasets from the GP5d binary STARR-seq experiment using 24 different hyperparameter combinations (*x* axis; Methods and Supplementary Note) to classify between active and inactive promoter–enhancer pairs. The training datasets used were the ‘paired’ set retaining the promoter–enhancer pairing, the ‘permutated’ set with the pairs shuffled and ‘enhancer from input’ and ‘promoter from input’ with the promoters and enhancers, respectively, paired with a randomly sampled inactive sequence from the input library. The classifiers trained on paired data (blue) outperform classifiers trained on enhancer (violet) or promoter (red) data, but not those trained with permutated data (green, paired Student’s *t* test two-sided *P* value = 0.134) (P_i_ = promoter from ith pair, E_i_ = enhancer from ith pair, E_k_ = enhancer from kth pair, I_k_ = input sequence from kth pair). **d**, TFs control transcription by directly or indirectly affecting chromatin structure (left), displacing nucleosomes and opening local chromatin (middle) and recruiting and positioning RNA polymerase II (Pol II) (right). Gene regulatory unit with classical (orange) and chromatin-dependent (dark blue) enhancers interacting with Mediator (light blue) and a promoter (brown) is shown. TFs with chromatin-dependent enhancer (FOXA and SOX), classical enhancing (YY1 and ETS), promoting (ETS, CREB and NRF1) and TSS-determining (TATA and YY1) activities are also indicated. The relative nonspecificity of interactions among TFs, classical enhancers and promoters suggests an important role of nonspecific interactions such as steric hindrance (size exclusion^47^ and nucleosome-mediated cooperativity^49^) in transcriptional regulation. The model is also consistent with other low-selectivity processes such as phase separation and recruitment in transcription^50^.

**Extended Data Fig. 9:**
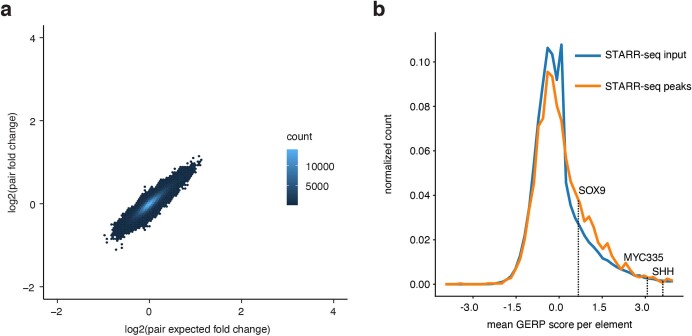
Enhancer–promoter interactions and conservation of mammalian enhancers in STARR-seq data. **a**, A control plot with comparison similar to that shown in Fig. 7a but performed using a set of PWMs that were reversed but not complemented. Note that the enrichment overall is much lower, and that the variance is similar to that observed in Fig. 7a, suggesting that most of the variance in Fig. 7a can be explained by random sampling and not biological effect. **b**, Conservation of GP5d genomic STARR-seq peaks (orange) and genomic STARR-seq input fragments (blue) measured with average GERP (genomic evolutionary rate profiling) scores. Higher GERP score means higher conservation. Three well-known mammalian enhancers are highlighted (see Methods for details). The average number of base pairs that are more conserved than the average coding base pair in the genomic STARR-seq peaks (170-bp region centered on the peak) was 51 for average peak and 119 for a set of known conserved and biologically important enhancers (MYC335, SOX9 and SHH); this clearly exceeds the ~7 bp conservation expected from the ~15-bit complexity of the TF motifs contained in the active elements derived from random sequence. These results suggest that regulatory elements *in vivo* are under more complex selection than elements selected for transcriptional activity in our assay.

The binary STARR-seq approach allows identification of interactions between promoters and enhancers. For this analysis, we counted single motif matches at the promoter and enhancer positions and all pairs of motif matches. When promoters and enhancers were analyzed separately, almost all pairs of TF motifs enriched independently of each other. Strikingly, even across promoters and enhancers, all motifs were independently enriched (Fig. 7a), suggesting that TFs bound to enhancers activate promoters, but in a very nonspecific manner. Some highly enriched TF–TF motif pairs, however, displayed weaker activity than that expected from a model that assumes additive action of the enhancer and promoter (Fig. 7b). In addition, three TF–TF motif pairs displayed stronger transcriptional activity than that expected from independent action of the individual TFs (Fig. 7a and Extended Data Fig. 9a); all three pairs combined a p53 family motif at the promoter with a repressive motif at the enhancer (Fig. 7a). These results are consistent with a model in which enhancer and promoter activities are integrated into total transcriptional activity; the observed saturation is consistent with a strong promoter not needing an enhancer and with a strong enhancer rendering weak and strong promoters equally active.

Unbiased machine learning analysis also supported a general mechanism of integration of promoter and enhancer activities (Fig. 7c). A CNN classifier using only promoter sequences outperformed a classifier using only enhancer sequences. As expected, combining the promoters with the correct enhancer sequences increased performance substantially. However, permutating the pairings between the promoters and enhancers resulted in similar performance (Fig. 7c), indicating that there was no predictive power in the specific pairing of individual promoters and enhancers. Taken together, our results indicate that the mechanisms that control transcription are very general and that the activities of almost all TFs can independently contribute to transcriptional activity.

## Discussion

Learning the rules by which DNA sequence determines where and when genes are expressed has proven surprisingly hard, despite the availability of full genome sequences of several mammals, extensive maps of genomic features^6,11,13^ and genome-scale data about TF protein expression levels and TF DNA binding in vitro^2,4,5^. Direct comparison of activities between TFs has remained difficult, and therefore, we generally lack parameters describing the relative strength of the different sequence features and their interactions—features that are critically important for prediction of transcriptional activity. To address this, we have here defined sequence determinants of human regulatory element activity in an unbiased manner, using an approach in which genomic, designed and random sequences are identified that display promoter or enhancer activity.

We found that the cellular gene regulatory system is relatively complex, consisting of several distinct kinds of elements. Motif grammar is relatively strong at the level of heterodimers but weaker at the level of spacing and orientation of specific TF motif combinations. In transcriptionally active sequences, precise TF arrangements such as those found in the interferon enhanceosome^31^ are rare, with most elements consisting of TFs acting together in a largely additive manner^18,20,32–35^. Our results are consistent with a recent report showing that independent actions of TFs can explain over 92% of the transcriptional activity measured from random yeast promoters^14^. The presence of a weak motif syntax is also consistent with known existence of spacing and orientation preferences of TFs in vitro^24^ and at human genomic enhancers^36^ and synthetic yeast promoters^37^.

Our results also show that different cell types have very similar TF activities and that the topology of the gene regulatory network is hierarchical, with few TFs displaying very strong transactivation activity. This is consistent with our previous work showing that relatively few TFs show strong DNA-binding activity in a cell and that many of the strong binders are common to various cell types^17^. Our findings contrast with the known tissue specificity of many putative enhancer elements in vivo^38^. Interestingly, the level of conservation of many endogenous promoters and enhancers appears to be higher^39^ than the elements selected in our assay (Extended Data Fig. 9b). The simplest explanation for these two facts is that enhancers in vivo evolve to be specific and that due to the similarity between cells, specificity is more difficult to achieve than activity. Specificity will naturally require specific TF combinations and also fine-tuning using motif number, spacing, orientation and affinity (e.g., Panne et al.^31^ and Crocker et al.^40^). Specificity is also required to silence strongly active elements in cell types in which their target protein is not needed due to the substantial fitness cost of protein expression^41^. Further analysis using main cell types representing all three germ layers is needed to determine whether and to what extent differentiated human cell types have retained the regulatory mechanisms that existed in their common unicellular ancestor. Moreover, the contribution of specific TFs to the transcriptional activity in the cell could be further dissected, for example, by testing mutated genomic fragments in MPRAs.

The original functional definition described enhancers as genetic elements that can activate a promoter from a distance, irrespective of their orientation relative to the TSS^8^. We find here that in addition to these elements, two other types of enhancing gene regulatory elements exist: chromatin-dependent enhancers and closed-chromatin enhancers (Fig. 7d). Chromatin-dependent enhancers are characterized by forkhead motifs, binding of Mediator and p300 protein and a strong signal for H3K27 acetylation. Unlike classical enhancers, chromatin-dependent enhancers do not transactivate a heterologous promoter strongly, most likely due to lack of binding of TFs with strong transactivator domains. Their presence is, however, strongly predictive of tissue-specific gene expression, suggesting that they act to increase gene expression via chromatin modification or structural changes in higher-order chromatin. Several chromatin-dependent enhancers also combine with a single classical enhancer to form superenhancers (see Fig. 4b), indicating that these elements may be required for driving high levels of gene expression from distal promoters. The third element type, closed-chromatin enhancers, are located in regions that show little or no signal for DNase I hypersensitivity or ATAC-seq. They are not silenced by CpG methylation. These elements appear to consist of only a single TF (e.g., p53; see also Peng et al.^42^) or a set of closely bound TFs that fit between or associate directly with well-ordered nucleosomes^43^. The prevalence of both the closed-chromatin enhancers and chromatin-dependent enhancers suggests that they may contribute substantially to regional control of gene expression^35^.

By using machine learning approaches, we show here that transcriptional activity in human cells can be predicted from sequence features (see also Avsec et al.^36^ and Agarwal and Shendure^44^). Interestingly, we found that the promoters enriched from completely random synthetic sequences in a single experimental step are even more predictive of transcriptional activity than the genomic sequences themselves. By analysis of de novo promoters enriched from random sequences, we discovered a G-rich element that interacts with the TSS, potentially positioning RNA polymerase II to the TSS independently of the TATA box. Overall, TF activities could be classified into three groups: TSS position–determining activity (e.g., TATA box and YY), short-range promoting activity (e.g., NRF1) and enhancing activity (many TFs). We did not detect a separate class of distal enhancing activity, suggesting that activities that would allow an enhancer to selectively act at a very long range are likely to be associated with chromatin-dependent enhancers and not classical enhancers^45,46^. The three classes of activities detected are not mutually exclusive, suggesting that TFs act at multiple levels and/or scales to regulate transcription (Fig. 7d). For example, YY1 acts as both an enhancing TF and a TSS-determining one, and FOXA motifs are present at both chromatin-dependent and classical enhancer elements. Our results thus indicate that TF motifs are the atomic units of gene expression and should be the ultimate basis of analysis and prediction of genomic elements controlling gene regulatory activity.

Our random promoter–enhancer design allowed unbiased discovery of features that facilitate interactions between classical enhancers and promoters at a relatively short range. No specific pair of motifs controlling such interactions was found. This, together with the fact that no specific TF that only acts from an enhancer was found, is consistent with a generic and indirect mechanism of action, where the activities of individual TFs bound to an enhancer are aggregated and their total activity then activates the promoter. Molecularly, these results are consistent with mediation of the effect by the least specific type of biochemical interaction, steric hindrance. The simplest mechanism for enhancer action would involve opening of higher-order and local chromatin in such a way that the steric hindrance that prevents large macromolecular complexes such as Mediator or RNA polymerase II from loading to DNA is decreased (Fig. 7d and Maeshima et al.^47^). However, in the highly evolved genomic context, more specific interactions can exist between chromatin-dependent enhancers and particular promoters, as reported in a few cases, such as multichromosome structures that control the expression of the repertoire of olfactory receptor genes or the complex regulatory landscape of HOX genes^48^.

In summary, we show here that direct experimentation to interrogate transcriptional activities of sequences that represent on aggregate >100 times larger sequence space than that of the human genome can be used to determine mechanisms of action of, and interaction between, gene regulatory elements. The experiments revealed unexpected simplicity of gene regulatory logic. The discovery of the relative simplicity of the interactions, together with the ability to measure transcriptional activities of all TFs in a cell, represents a major step toward achieving the ultimate aim of regulatory genomics: predicting gene expression from a sequence.

## Methods

### STARR-seq vector design

We designed a modified STARR-seq reporter construct pGL4.10-Sasaki-SS (a) based on an earlier published design^15^ in the pGL4.10 backbone (Promega, E6651). The sequence between *Sac*I and *Afe*I was replaced with a sequence containing CG-depleted chicken lens δ1-crystallin gene (Sasaki) promoter^51^, a synthetic intron (pIRESpuro3; Clontech, 631619), an ORF (fusion of Nanoluc-EmGFP), homology arms for library cloning with *Age*I and *Sal*I restriction enzyme (RE) sites flanking the *ccdB* gene, a small 52-bp DNA stuffer (a part of the neomycin resistance cassette) and a 20-bp sequence from the 3ʹ-Illumina adapter for optimally sized final library for Illumina sequencing and the SV40 late poly(A) signal from the pGL3 backbone (Promega, E1751).

To enable the analysis of CpG methylation on enhancer activity, we designed modified STARR-seq vectors in a CpG-free backbone with Lucia reporter gene (Invivogen, pcpgf-promlc) driven either by the EF1α promoter (b. pCpG-free-EF1α-SS) or the Sasaki promoter (c. pCpG-free-Sasaki-SS-v1) as above. To facilitate the cloning of the synthetic DNA library to the 3ʹ UTR of the reporter gene, the cloning cassette from the pGL4.10-Sasaki-SS vector (a) containing the homology arms with *Age*I and *Sal*I RE sites, the 52-bp DNA stuffer and the 20-bp sequence from the 3ʹ-Illumina adapter as above was introduced to the CpG-free vectors using the *Nhe*I site.

Standard Illumina adapters harbor CG dinucleotides, and to make our modified STARR-seq design completely CpG-free, we designed custom adapters for Illumina sequencing (oligos 3 and 4 in Supplementary Table 2). To accommodate the cloning of genomic DNA and random sequence inserts with flanking CpG-free custom adapters, the cloning cassette in CpG-free-Sasaki-SS-v1 was modified by removing the 3ʹ-Illumina adapter and the 52-bp stuffer. In addition, this vector was further improved by replacing the *Age*I and *Sal*I RE sites with the *Afl*II and *Pvu*II sites devoid of CG dinucleotides and introducing a DNA stuffer of 1.2 kb between the RE sites to the resulting pCpG-free-Sasaki-SS-v2 vector (d) to unambiguously detect and purify the linearized reporter backbone for downstream cloning.

For the binary STARR-seq approach in which random sequences were cloned as both promoters and enhancers, the pCpG-free-Sasaki-SS-v2 vector (d) was modified by replacing the Sasaki promoter with a custom CpG-free 5ʹ-adapter sequence and an *Age*I RE site and introducing a *Sal*I RE site and a custom CpG-free 3ʹ adapter immediately downstream of the ORF. Moreover, to optimize the random promoter and random enhancer library size for Illumina sequencing, the Lucia reporter gene was replaced by a small 11-amino-acid ORF from *Drosophila melanogaster* (Dm tal-1A) in the pCpG-free-promoter–enhancer-SS vector (e). The cloned random promoter–random enhancer input library is paired-end sequenced to map the promoter–enhancer pairs, and thus, the random enhancer sequences obtained after sequencing the reporter-specific RNA library can be used to identify the corresponding promoter sequence from the input library. In total, the constant sequence between promoter and enhancer elements is 872 bp in the pCpG-free-Sasaki-SS-v2 construct and 215 bp in the pCpG-free-promoter–enhancer-SS construct.

The new reporter vectors (a–e) were used in different experiments as follows (their complete sequences are provided in Supplementary Table 1): a, pGL4.10-Sasaki-SS (5,754 bp) was used for experiments with the synthetic motif library shown in Extended Data Fig. 1b ; b, pCpG-free-EF1α-SS (3,497 bp) was used for all experiments with the synthetic motif library; c, pCpG-free-Sasaki-SS-v1 (3,388 bp) intermediate plasmid was not used in the experiments; d, pCpG-free-Sasaki-SS-v2 (4,458 bp) was used for all experiments with genomic fragments and random enhancer (N170) sequences; and e, pCpG-free-promoter–enhancer-SS (2,551 bp) was used for all experiments with random promoter (N150)–random enhancer (N150) sequences.

### STARR-seq reporter library construction and cloning

STARR-seq reporter libraries were generated from rationally designed oligonucleotides harboring TF binding motifs, from fragmented human genomic DNA and from synthetic oligonucleotide with completely random DNA sequences as detailed in the Supplementary Methods. All the oligonucleotides that were used for cloning of the libraries were purchased from Integrated DNA Technologies, and their sequences are provided in Supplementary Table 2.

### CpG methylation of STARR-seq input DNA library

The genomic DNA library was methylated using *M.SssI* (New England Biolabs) for 4 h at 37 °C with the reaction volumes scaled for 62.5 µg plasmid DNA per reaction and inactivated for 20 min at 65 °C, followed by purification and ethanol precipitation of the methylated library.

### Cell lines and generation of *TP53*-null cell line by genome editing

The cell lines used in this study were the colon cancer cell line GP5d (Sigma, 95090715), the liver cancer cell line HepG2 (ATCC, HB-8065) and the retinal pigment epithelial cell line hTERT-RPE1 (ATCC, CRL-4000). The cells were maintained in their respective media (GP5d in DMEM, HepG2 in MEM and RPE1 in DMEM/F12) supplemented with 10% fetal bovine serum, 2 nM l-glutamine and 1% penicillin–streptomycin.

The *TP53*-null GP5d cell line was generated by CRISPR-Cas9 targeting of exon 4 of the *TP53* gene using Alt-R CRISPR-Cas9 from Integrated DNA Technologies. Briefly, annealed sgRNA duplex from crRNA (oligo 12; Supplementary Table 2) and tracrRNA with atto550 were used for ribonucleoprotein complex formation with Cas9-HiFi protein, and the ribonucleoprotein complex was transfected to GP5d cells using CRISPRMAX (Invitrogen). The next day, atto550^+^ cells were FACS sorted, and single-cell colonies were cultured to produce a clonal *TP53*-null cell line. The clonal cells lines were screened for TP53 depletion by western blotting, and clones were verified by Sanger sequencing using oligos 13 and 14 (Supplementary Table 2).

### Transfection and RNA isolation

In STARR-seq experiments, 1 µg of each input library DNA was transfected per million cells. For TF motif DNA libraries, a total of 50 and 35 million GP5d cells were transfected for the libraries in the pGL4.10-Sasaki-SS (a) and pCpG-free-EF1α-SS (b) vectors, respectively. Experiments were performed in two replicates with random enhancer libraries in GP5d and HepG2 cells and random promoter–enhancer libraries in GP5d cells (250 million cells per each replicate). Genomic STARR-seq experiments were performed in two replicates in HepG2 cells (170 million cells per replicate) and four different conditions in GP5d cells (wild-type and *TP53*-null GP5d cells using both methylated and nonmethylated input DNA libraries; 500 million cells per condition). For random promoter–enhancer libraries in HepG2 and RPE1 cells, a total of 400 and 480 million cells were transfected, respectively. Briefly, a day before transfection, 6.7–10 million cells were plated per 15-cm dish in their respective media without antibiotics. The next morning, plasmid DNA was mixed with transfection reagent optimized for each cell line (Transfex (ATCC) for GP5d, Transfectin (Bio-Rad) for HepG2 and FuGENE HD (Promega) for RPE1) at a 1:3 ratio in Opti-MEM medium (Gibco), incubated for 15 min at room temperature and added dropwise to the cells. The cells were incubated for 24 h in a 37 °C incubator with 5% CO_2_.

Cells were harvested and total RNA isolated 24 h after transfection using the RNeasy Maxi kit (Qiagen) with on-column DNase I digestion. The poly(A)^+^ RNA fraction was purified using the Dynabeads mRNA DIRECT Purification kit (Invitrogen) followed by DNase treatment using TurboDNase (Ambion) and purification using RNeasy Minelute kit (Qiagen) as previously described^15^.

### STARR-seq reporter library and input DNA library construction

The library preparation protocol was adapted from Arnold et al.^15^ essentially in all steps but with primers matching our modified STARR-seq vectors, and the exact protocol is described in Supplementary Methods.

### Template-switch library preparation

To generate a sequencing library using a template-switch strategy, a 40-µg aliquot of total RNA from the random promoter–random enhancer STARR-seq experiment in the GP5d cell line was used. See Supplementary Methods for the detailed protocol and section ‘Mapping TSS positions based on template switching’ for respective data analysis.

### ChIP-seq, RNA-seq, CAGE, DNA methylation and ATAC-seq

ChIP-seq was performed as previously described^52^ using the following antibodies (2 μg per reaction): H3K27ac, H3K9me3 and H3K27me3 (Diagenode, C15410196, C15410193 and C15410195, respectively); FOXA1 (Abcam, ab23738); p53, HNF4a, IRF3 and CTCF (Santa Cruz Biotechnology, sc-126x, sc-8987x, sc-33641x and sc-15914x, respectively); SMC1 (Bethyl Laboratories, 300-055A); and normal rabbit, mouse and goat IgG (Santa Cruz Biotechnology, sc‐2027, sc‐2025 and sc‐2028, respectively). To analyze the genomic occupancy of TP53, GP5d cells were treated with 350 µM 5-fluorouracil (Sigma) 24 h before harvesting the cells. To analyze the effect of STARR-seq plasmid transfection on cellular alarm signals by ChIP-seq, RNA-seq and ATAC-seq, HepG2 cells were collected 24 h after the following treatments: mock (DMSO), 350 µM 5-fluorouracil treatment and transfection of the genomic STARR-seq library using similar conditions as in the STARR-seq experiments described above. The details of conditions, protocols and analysis parameters are described in Supplementary Methods.

For RNA-seq, total RNA was isolated using RNeasy Mini kit (Qiagen) and RNA-seq libraries were generated using KAPA stranded mRNA-seq kit for Illumina (Roche). CAGE library was prepared from total RNA isolated from GP5d cells as previously described^53^ from 1 µg total RNA. The bisulfite sequencing data for DNA methylation in GP5d cells were obtained from Yin et al.^5^. The ATAC-seq libraries were prepared from 50,000 cells as previously described^54^ for GP5d and HepG2 cells. All samples were paired-end sequenced using Illumina platforms.

### ATI and TT-seq assay

The ATI assay in GP5d cells and processing of the data were done as previously described^17^. Transcribed enhancer regions defined using TT-seq data are based on Lidschreiber et al.^55^. The experiments were performed from GP5d cells in two biological replicates as previously described^56^. The details of the protocols and analyses are described in Supplementary Methods.

### Motif collection and library design for enhancer analysis

For testing activities of known TF motifs, a set of 3,226 HT-SELEX motifs were collected (refs. ^4,5,57^ and unpublished draft motifs). The motif collection and library design, measurement of enhancer activity of TF motif consensus sequences, and generation of activity PWM are described in detail in the Supplementary Methods. Moreover, previously described promoter motifs were used in TSS analyses, including TATA box, initiator, CCAAT-box and GC-box^58^; BRE, MTE and DPE^59^; and BANP^25^.

### Library complexity analysis

The complexity analysis of STARR-seq libraries generated from the TF motifs, genomic DNA and random enhancer libraries are described in Supplementary Methods; read counts are given in Supplementary Table 7.

### Preprocessing of random enhancer library sequences

First, 150-base-long STARR-seq RNA and input DNA paired-end reads were combined using the FLASH program^60^ (version 1.2.11; options —min-overlap = 130 —max-overlap = 134 −*x* 0.25 −z −*t* 4), and only combined sequences of length 170 were chosen. To avoid including PCR duplicates of the same sequence with few mismatches due to sequencing errors, the sequences were sorted four times based on 40-bp-long nonoverlapping subsequences from base 6 to 165, and only one sequence per identical subsequence at each sorting step was taken. This ensured that from sequences that had Hamming distance less than 4, only one was taken. Only one representative sequence from the similar sequences was used for downstream analysis, so each sequence is either present or absent in the sample. The sequences are sampled from a huge input DNA library, which prohibits precise determination of initial input frequencies of individual sequences. Thus, our analysis relies on finding common features of different selected sequences instead of their counts. The numbers of preprocessed sequences used in downstream analysis are shown in Supplementary Table 7.

### Genomic STARR-seq analysis and its features

The active enhancers were identified by calling the peaks from the STARR-seq–enriched RNA fragments against the plasmid input sample using MACS2 (ref. ^61^). The preprocessing of genomic STARR-seq data, peak calling, overlap with genomic features, de novo motif mining and conservation of genomic STARR-seq elements are described in detail in Supplementary Methods.

### Preprocessing of the random promoter–enhancer pairs

The STARR-seq enhancer sequences derived from RNA were mapped to corresponding promoter–enhancer pairs in the input DNA by exact matches of the first 20 bases of the 150-base-long enhancer sequences. Duplicate sequences were removed as described for random enhancers, except that three 40-bp-long subsequences from 16 to 135 were used, thus ensuring that only one of the sequences with Hamming distance less than 3 was chosen (Preprocessing of random enhancer library sequences). Then, promoter and enhancer sequences were filtered separately by removing (1) all adapter sequences that included some (partial) adapter sequence according to cutadapt version 1.9.1 (ref. ^62^), (2) sequences that mapped to plasmid backbone sequence using bowtie2 version 2.2.4 (ref. ^63^) and (3) outlier sequences in terms of nucleotide composition (count of any nucleotide more than three median absolute deviations higher than the median count) that removes, for example, those high-G-content sequences that are an Illumina artifact. After preprocessing, the correlation between observed dinucleotide frequencies and those expected based on mononucleotide frequencies was over 0.99 (GP5d random replicate 1 enhancers). Input DNA sequences were processed the same way. For promoter–enhancer pair analyses, the remaining promoter–enhancer pairs were collected, and pairs containing highly similar sequence as a promoter and an enhancer were removed. The numbers of sequences used in downstream analysis are shown in Supplementary Table 7.

### Mapping TSS positions based on template switching

First, the synthetic random sequences derived from spliced transcripts were identified using the constant sequence spanning the splice site after intron removal (cutadapt program); other sequences were not processed further. Next, the UMI sequence was removed from the 5ʹ end of each sequence, and the last 20 bp of its random part was used to recognize the corresponding promoter from the input DNA. To accurately recognize the first base of the transcript and thus the position of the TSS, it was assumed that the template-switch process had added at least three and at most four guanines to the 5ʹ end of the transcript. On this basis, only the RNA sequences starting with at least three Gs were used in the analysis. Each such sequence was aligned to the corresponding input DNA promoter sequence using an exact 20-bp match starting from the sixth base to allow for the extra Gs. Finally, the Gs added by the template switch were trimmed and discordant sequences removed according to the alignment. The frequency of the four Gs instead of three was estimated from the sequences that do not have G at the fourth position in the alignment to the input. For those that did have a G also in the input sequence, removing three or four Gs was decided randomly but so that the frequency of the fourth G matched the estimate. The two GP5d template-switch libraries were processed separately and then merged so that only one transcript was kept for each input DNA promoter sequence to prevent duplicate promoter sequences. The exact positions of the TSSs at the promoters were recorded, and the flanking sequences were used for further analysis of the positioning of different sequence features relative to TSSs. The numbers of sequences obtained are listed in Supplementary Table 7, as the number of flanking sequences fitting to the random region depends on the flank sizes. The comparison to human endogenous promoters was done using TSS positions from EPD^30^ (hsEPDnew 006).

### Matching of known motifs and analysis of motif spacing

The motif matching was done using MOODS version 1.9.3 (ref. ^64^), and fold changes were estimated using the function PsiLFC in R package lfc. The details of motif matching and motif spacing analysis in STARR-seq random enhancers are described in Supplementary Methods.

### Analysis of interactions between the promoter and enhancer

For RNA and randomly sampled input DNA promoter–enhancer pairs, the number of such pairs that one motif occurs in the promoter and a second one in the enhancer was counted for each motif pair (excluding heterodimers) and motif-match strand combination (++, +−, −+ and −−). The counts over the strand combinations were summed to get the total number of pairs, and the fold change of the number of pairs between input DNA and RNA was estimated using the function PsiLFC in R package lfc. If the promoter and enhancer occurrences are independent of each other, then the expected frequency of the pair of sites is the product of the individual frequencies. The expected log_2_ fold change assuming independent actions of the promoter and enhancer motif was thus calculated as the sum of their individual log_2_ fold changes. The same analysis was done using the reversed, but not complemented, control motifs.

To estimate the significance of the number of observed motif pairs, our null hypothesis was that the probability of observing a motif-match pair in a promoter–enhancer sequence pair was the same as estimated from the individual motif-match frequencies. The two-sided binomial tests done for 528,529 motif pairs resulted in a significant *P* value (multiple hypothesis-corrected *P* value < 0.05, Holm’s method) for 253 pairs.

### Motif-match positioning relative to TSS and STARR-seq vector

For analyzing motif positioning within active promoters derived from synthetic random sequences, motifs were matched to sequences flanking the TSSs identified from the template-switch data, and for each motif, only the highest-affinity match per sequence was considered. The number of matches for each motif was then counted separately at each position and strand. To get positional activity scores for position-specific regression analysis, motif matching was done for TSS flanking sequences from position −100 to +20 in relation to TSS and for a control set generated by sampling for each TSS sequence a subsequence of same length from the same position from an input DNA promoter (background probability of a match 5 × 10^−4^). The log_2_ fold changes of the motif match counts between TSS flanking set and control set (estimated with the lfc package) were then used as a positional activity score for each position and strand.

To study p53 motif-match positioning relative to the STARR-seq vector, the motif was matched (background probability of a match 10^−5^) to highly selected sequences chosen by taking only sequences observed at least twice in both GP5d enhancer replicate experiments. A histogram of match start positions was generated by counting only the highest-affinity match in each sequence. A smoothed density estimate was generated using a Gaussian kernel (R ggplot geom_density with adjust=0.5).

### Mutual information analysis

The mutual information analysis was done as previously described^65^ for the aligned STARR-seq reads (60 + 60 bp surrounding the TSS from the template-switch data); details are described in Supplementary Methods.

### Data preprocessing for machine learning analysis

The datasets used in each machine learning analysis and their division into training, test and validation sets are detailed in Supplementary Table 7. To enable sequences from genomic measurements (genomic STARR-seq and ATAC-seq) to be scored on the CNNs that were trained on the random enhancer STARR-seq data and vice versa, the length of the sequences fed to these models was standardized to 170 bp. Thus, additional preprocessing specific to machine learning analyses was done for the genomic STARR-seq and ATAC-seq data as detailed in the Supplementary Methods.

### Machine learning analysis

The details of machine learning analyses using logistic regression and CNNs, discussion about the optimal classification of random enhancer STARR-seq data, details of differential expression prediction, interpretation of CNN classifiers and validation of the predicted promoter mutation effects with external data are described in Supplementary Methods. Briefly, the logistic regression classifiers were implemented using the LogisticRegression function from scikit-learn (version 0.21.3) library^66^; the CNN models were built on Keras (https://keras.io/; version 2.2.4) using TensorFlow 1.14.0 backend^67^; DeepLIFT version 0.6.12.0 (ref. ^68^) was used to visualize the *TERT* promoter mutations and study the sequence features learned by the random enhancer STARR-seq CNN model along with TF-MoDISco version 0.5.14.1 (ref. ^69^).

### Promoter–enhancer interaction analysis using machine learning

The binary STARR-seq design allows looking for relatively short-range interactions between promoters and enhancers, and the details of machine learning analysis used for testing such interactions are detailed in the Supplementary Methods.

### TSS prediction

All the promoter models were trained on data where the TSS is 100 bp from the start of the training sequence. Thus, scoring any 120 bp sequence with these models gives a probability that the position 100 in these sequences is a TSS of a functional promoter sequence. Each possible TSS position within ±500 bp from the TSSs of the active GP5d promoters was analyzed by taking 100 bp upstream and 20 bp downstream from the candidate TSS and scoring these sequences with the promoter models. Active GP5d promoters were defined as those EPD promoters that overlapped with a GP5d CAGE peak. For each test set, active GP5d TSS and promoter model, the position obtaining the highest promoter probability from the corresponding model was taken as the predicted TSS position.

### Preprocessing of genomic promoters and CAGE analysis

Human promoter coordinates were obtained from the EPD version 006, hg19 (ref. ^30^), and their preprocessing, together with analysis of the CAGE data, is described in Supplementary Methods.

### Reporting Summary

Further information on research design is available in the Nature Research Reporting Summary linked to this article.

## Online content

Any methods, additional references, Nature Research reporting summaries, source data, extended data, supplementary information, acknowledgements, peer review information; details of author contributions and competing interests; and statements of data and code availability are available at 10.1038/s41588-021-01009-4.

## Supplementary information

Supplementary Information Supplementary Figures 1–4, Note, Methods and References.

Reporting Summary

Peer Review Information

Supplementary Table Supplementary Tables 1–12

## Data Availability

All sequence data generated in this study are available under GEO accession GSE180158. All pretrained machine learning models are available at Zenodo with accession 10.5281/zenodo.5101420. Training, test and validation datasets for the CNN models are available at Zenodo with accession 10.5281/zenodo.5101420. The genome browser session is available at the University of California, Santa Cruz (UCSC) portal with tracks for all genomic datasets generated in this study (https://genome.ucsc.edu/s/kivioja/Sahu_et_al_Human_regulatory_elements). ENCODE blacklisted genomic regions for hg19 (accession ENCSR636HFF) were downloaded from ENCODE, and RepeatMasker file for hg19 was downloaded using the UCSC table browser. The EPD^30^ for human TSSs can be found online (https://epd.epfl.ch/EPDnew_database.php). In addition, transcript annotations downloaded from Ensembl (GRCh37, release 101) were used. The saturation mutagenesis results of the *TERT* promoter^28^ can be found online (10.17605/OSF.IO/75B2M). GERP conservation scores for the hg19 reference genome can be found online (http://mendel.stanford.edu/SidowLab/downloads/gerp/). The following datasets were downloaded from the ENCODE portal: ATAC-seq (ENCSR042AWH, replicate 1), histone modification ChIP-seq experiments for H3K27ac (ENCSR000AMO), H3K27me3 (ENCSR000AOL), and H3K9me3 (ENCSR000ATD) and H3K4me1 (ENCFF424GUI) and ChIP-seq datasets for TP53 (ENCSR980EGJ), MED1 (ENCFF493UFO) and MED13 (ENCFF003HBS). ChIP-seq peak sets were also downloaded from GEO accession GSE104247. Superenhancers for HepG2 were downloaded from http://www.licpathway.net/sedb. Previously published RNA-seq data used in the study have been deposited to the European Genome-phenome Archive (accession https://ega-archive.org/studies/EGAS00001002966).

## Code availability

All essential custom code is available at Zenodo with accession 10.5281/zenodo.5159644.
