# Supplementary material for: Sequence determinants of human gene regulatory elements
Source: Nat Genet. 2022 Feb 21;54(3):283–94. doi: 10.1038/s41588-021-01009-4 (PMC8920891; doi:10.1038/s41588-021-01009-4)
Supplement: Supplementary file 1 — Supplementary Figures 1–4, Note, Methods and References. [file 41588_2021_1009_MOESM1_ESM.pdf]

nature portfolio

https://doi.org/10.1038/s41588-021-01009-4

Supplementary information

Sequence determinants of human gene regulatory elements

In the format provided by the authors and unedited

Supplementary information for

Sequence determinants of human gene regulatory elements

Biswajyoti Sahu[1,2], Tuomo Hartonen[1], Päivi Pihlajamaa[1], Bei Wei[3,4], Kashyap Dave[3], Fangjie Zhu[5], Eevi Kaasinen[1,3], Katja Lidschreiber[6,7], Michael Lidschreiber[6,7], Carsten O. Daub[7,8], Patrick Cramer[6,7], Teemu Kivioja[1] and Jussi Taipale[1,3,5]*

1. *Applied Tumor Genomics Research Program, Faculty of Medicine, University of Helsinki, Helsinki, Finland*
2. *Medicum, Faculty of Medicine, University of Helsinki, Helsinki, Finland.*
3. *Department of Medical Biochemistry and Biophysics, Karolinska Institutet, Stockholm, Sweden*
4. *Department of Genetics, Stanford University School of Medicine, Stanford, CA, USA*
5. *Department of Biochemistry, University of Cambridge, Cambridge, United Kingdom*
6. *Department of Molecular Biology, Max Planck Institute for Biophysical Chemistry, Göttingen, Germany*
7. *Department of Biosciences and Nutrition, Karolinska Institutet, Stockholm, Sweden*
8. *Science for Life Laboratory, Stockholm, Sweden*

** Corresponding author: Jussi Taipale (ajt208@cam.ac.uk)*

The supplementary information contains:

Supplementary Figures 1-4

Supplementary Note

Supplementary Methods

Supplementary References

Supplementary Tables 1-12 (in a separate Excel file)

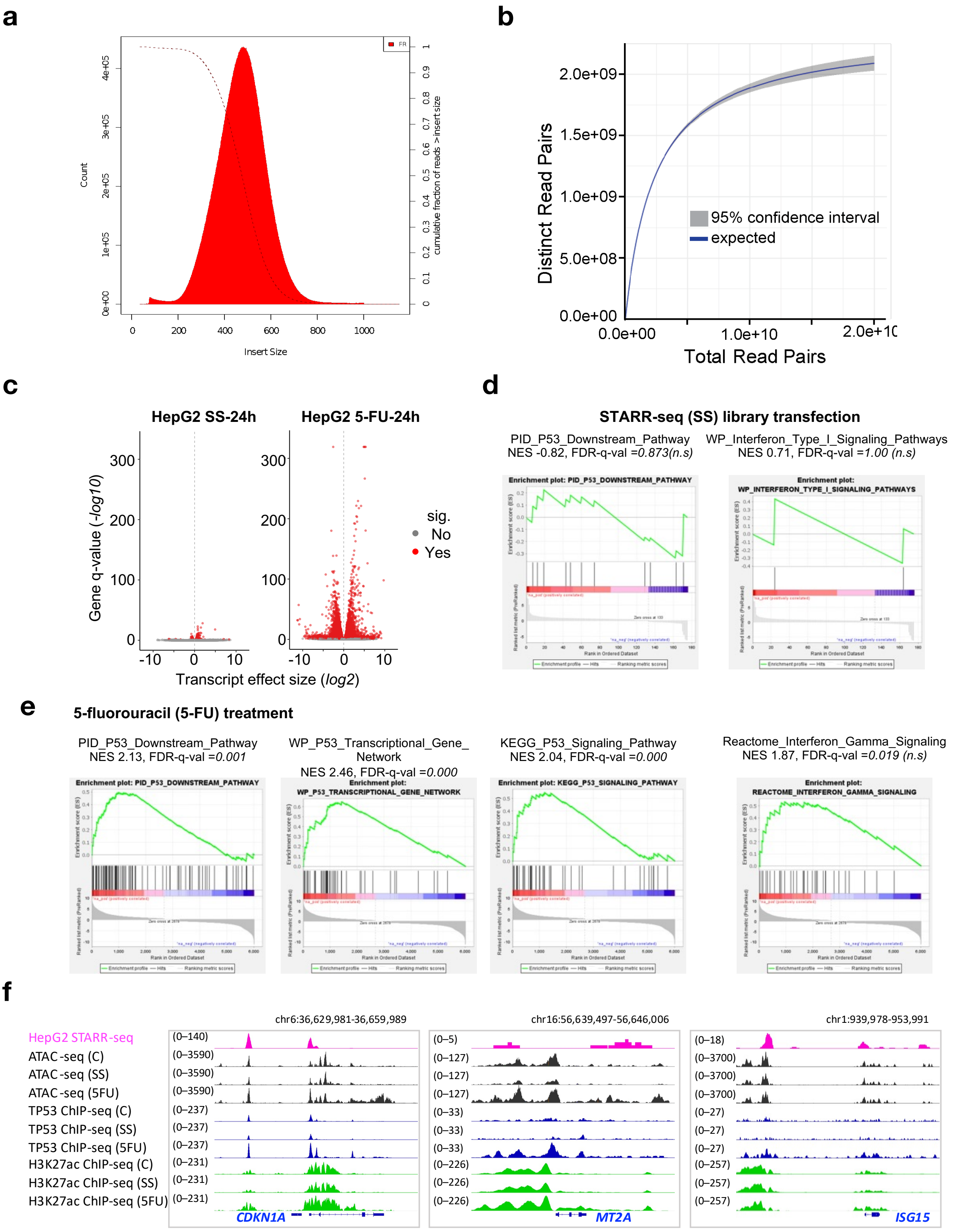

Supplementary Fig. 1. The complexity of the genomic DNA input STARR-seq library and analysis of cellular alarms in response to reporter library transfection.

a, Histogram showing the insert size distribution of input library comprising of human genomic DNA fragments.

b, Genomic STARR-seq input library complexity estimation. Blue line shows the expected number of distinct read pairs (y-axis) given a (hypothetical) number of sequenced read pairs (x-axis) estimated based on the copy numbers of the sequenced read pairs. The gray band shows

the confidence interval based on bootstrapping. Extrapolation of the sequenced random sample gives an estimate of over 2 billion distinct read pairs (fragments) in the library (see **Methods** for details).
c, Differential gene expression in response to STARR-seq library transfection and p53 induction. RNA-seq was performed from HepG2 cells 24 h after genomic STARR-seq library transfection (SS) or 5-fluorouracil (5-FU) treatment. Fountain plots show differentially expressed genes (q-value < 0.05; see **Methods**) from SS- and 5-FU samples compared to mock. The transcript effect size is the estimated effect size of the transcript with the smallest *P* value. Note that there are only 180 differentially expressed genes induced by the plasmid transfection (*left*), whereas 5-FU treatment results in 6273 differentially expressed gene (*right*).
d, e, Gene set enrichment analysis for p53 and interferon signaling pathways from differentially expressed genes in response to STARR-seq library transfection (**d**) and 5-FU treatment (**e**) in HepG2 cells. Note that there were no significantly enriched gene sets in the plasmid transfected cells; the two enrichment plots shown for reference in (**d**) have q-values of 0.873 and 1.00, respectively. However, robust and statistically significant activation of p53 targets as well as interferon gamma signaling pathway was observed in 5-FU-treated cells (**e**). The gene sets used in the analysis were obtained from the molecular signatures database (MSigDB).
f, Genome browser snapshots of three genomic loci, a p53 target gene (*CDKN1A*), and two target genes of the interferon pathway (*MT2A* and *ISG15*) showing enhancer activity from genomic STARR-seq data, chromatin accessibility from ATAC-seq, TP53 binding as well as H3K27ac from ChIP-seq data in HepG2 cells. Traces from cells transfected with STARR-seq plasmid, cells treated with 5-FU as well as control (mock) cells are shown. Note that plasmid transfection itself does not induce changes in TP53 binding to its target genes or changes in chromatin accessibility or H3K27ac at IRF target gene loci. Collectively, the results shown in **c-f** demonstrate that in HepG2 cells, the plasmid transfection does not elicit strong cellular alarm responses (see also ref. [1]).

a

De novo motif	TOMTOM	*p-value*	sites	*De novo* motif	TOMTOM	*p-value*	sites
	TP53	7.5e-6068	31351		FOXO3	8.4e-011	139162
	TP53	2.9e-242	1832		TP53	1.2e-010	471
	IRF3	1.8e-110	1049		IRF3	1.4e-008	142
	ELK1	4.9e-072	230761		GRHL	5.8e-007	995
	JDP2	4.6e-056	6212		TCF7L	3.2e-006	860
	TP53	2.1e-046	4018		FOXD3	5.6e-006	49359
	KLF1	6.8e-027	103174		TP73	2.9e-005	55
	ERG	5.2e-020	65121		HNF4	1.3e-004	25014
	IRF4	4.2e-015	168		IRF5	1.9e-004	35794
	NHLH1	1.1e-013	105668		SP8	9.6e-003	474
	HNF1	1.5e-011	882		HOXA13	1.3e-002	3657

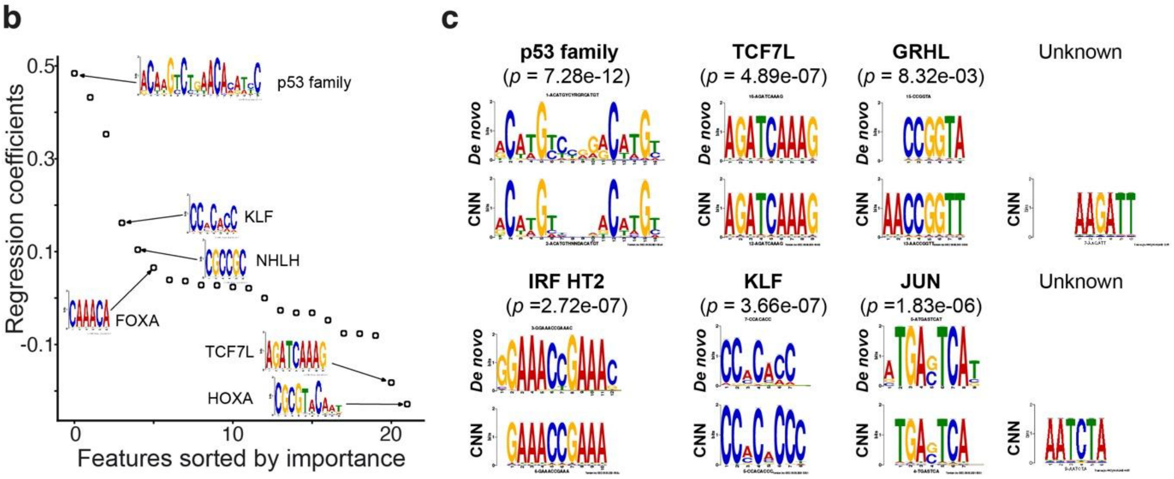

Supplementary Fig. 2. De novo motifs enriched from random enhancer STARR-seq data in GP5d cells and from sequences learned by the CNN.

a, *De novo* motifs enriched from random enhancer STARR-seq data analyzed using STREME program. The *P* values and the numbers of sequences matching the motif are shown as calculated by the STREME algorithm (no additional multiple hypotheses correction) along with the motif identity from TOMTOM (see **Methods** for details).

b, Regression coefficients (y-axis) for the 22 *de novo* motifs from random enhancer data (from panel **a**). The motif features with strongest regression coefficient values are highlighted. The regression model using the *de novo* motifs obtained a similar area-under precision recall curve (AUprc) score than the simple logistic regression using the 880 HT-SELEX derived motifs (for both, AUprc=0.55).

c, Comparison of the *de novo* motifs discovered from the top 0.5% highest-scoring random sequences according to the GP5d random enhancer CNN model (lower motif of each pair) and the best matching GP5d random enhancer STARR-seq *de novo* motif (top motif of each pair). If no significant match was found from the GP5d random enhancer STARR-seq *de novo* motifs (TOMTOM *P* value threshold 0.05), only the motif learned by the CNN model is shown. Bonferroni-corrected empirical *P* values reported by the TOMTOM program for the comparison between *de novo* motifs discovered from random enhancer STARR-seq and CNN-learned sequences are indicated for each pair shown in the figure.

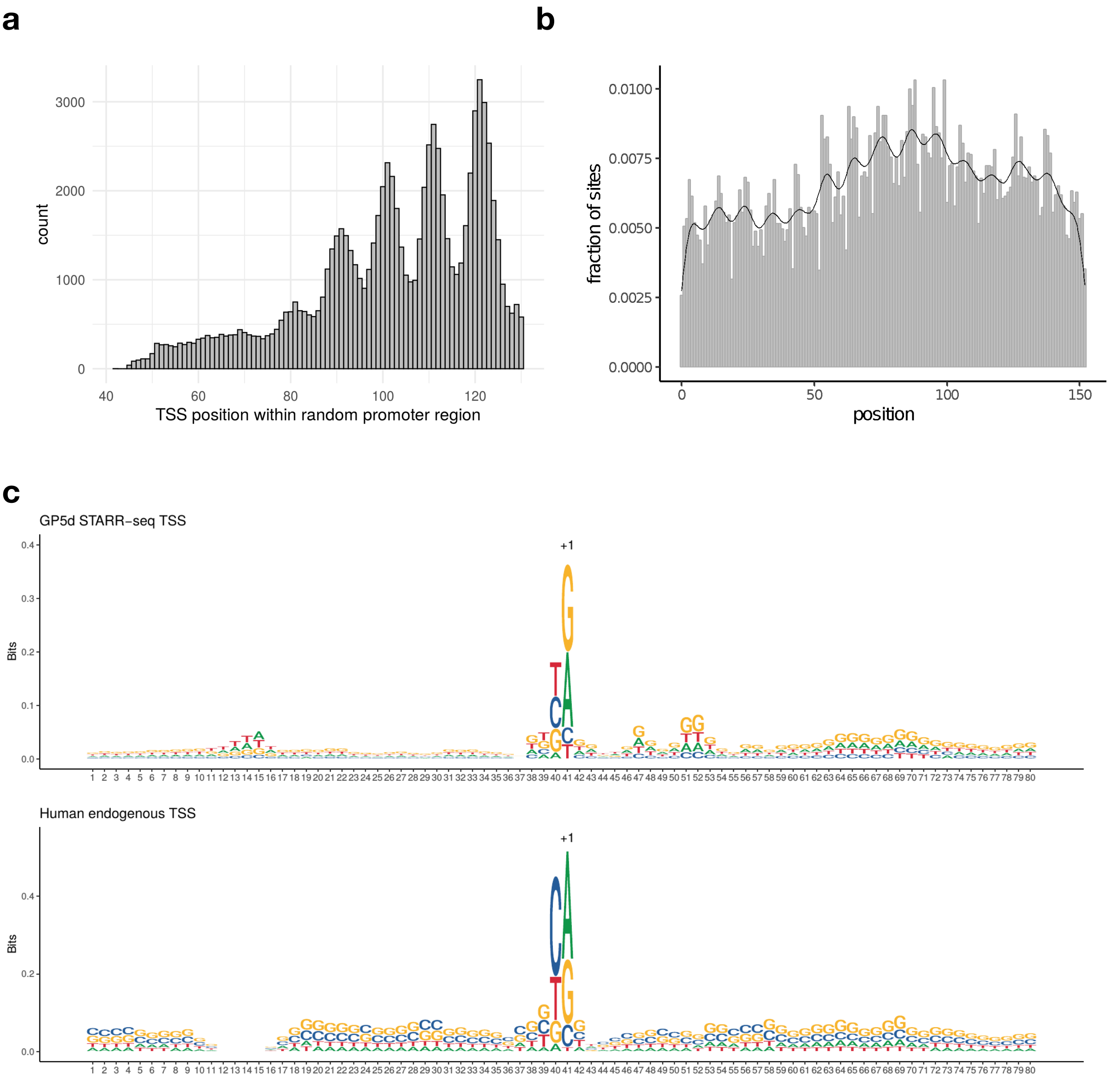

Supplementary Fig. 3. Features discovered from random promoter and random enhancer STARR-seq measurements.

a, Template switch reads used for positioning the TSS show a 10-bp periodicity relative to the STARR-seq vector.

b, Positioning of the p53 motif matches in the random enhancer. The fraction of p53 motif match sites starting at each position is shown. Black line shows the smoothened density estimate.

c, TSS enriched from random sequences (*top*) show similar features as human endogenous TSS (*bottom*). The G-rich element detected downstream of TSS from the random promoter STARR-seq data is also present in the human genome. However, to our knowledge it has not been recognized before, potentially because the feature is obscured by the general GC anisotropy (more guanines on the plus strand) both upstream and downstream of the TSSs which has been reported earlier (see ref. [2]) and may partly be caused by mutational mechanisms.

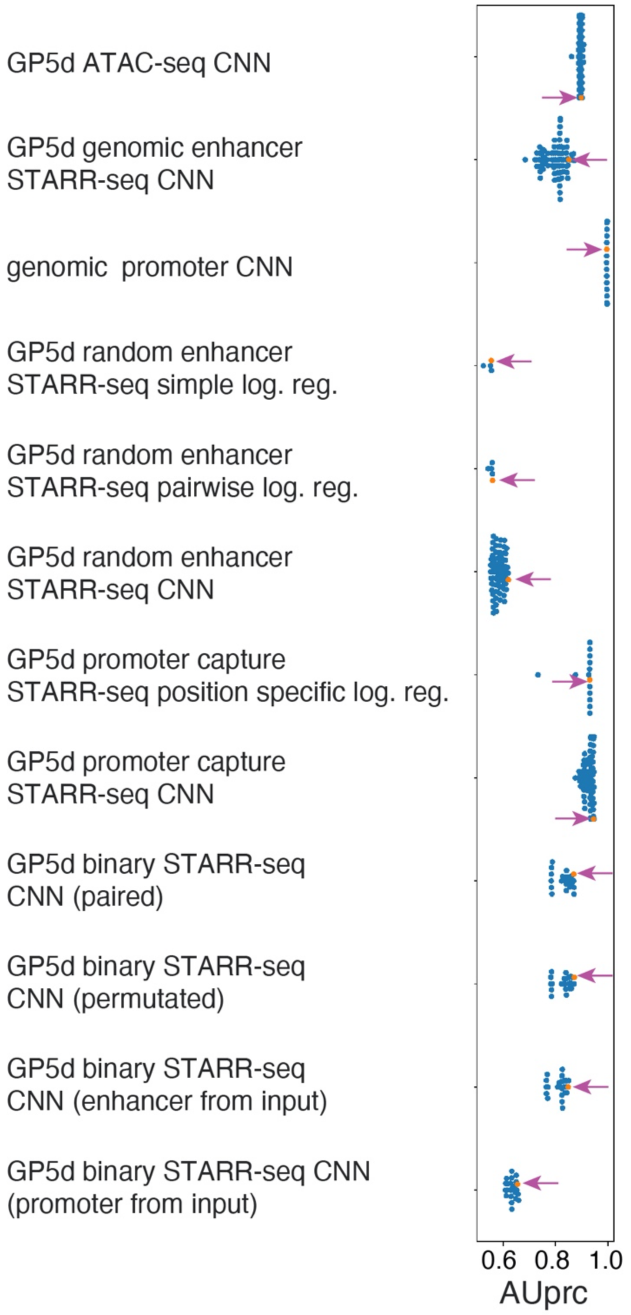

Supplementary Fig. 4. Testing machine-learning models for optimal hyperparameter combinations.

All tested hyperparameter combinations for each machine-learning classifier scored on unseen test data. Classes are balanced in each test set meaning that a classifier assigning samples with random labels with equal probabilities would obtain an AUprc score of 0.5. The final model for each hyperparameter combination was selected by maximizing binary accuracy on a separate validation dataset, unless otherwise stated in the **Methods**. Each dot in the swarm plot represents the test set AUprc for a final model for a certain set of hyperparameters. The exact hyperparameter combinations tested are listed in **Supplementary Table 8**. The model names on y-axis correspond to the given model names in **Supplementary Tables 8, 9**. The best model for each task (marked with an arrow) was selected based on validation data across all tested hyperparameter combinations for that task. The exact hyperparameters of the chosen models are listed in **Supplementary Table 9**.

Supplementary Note

Supplementary text to support the main conclusions.

1) **Few TFs display strong transcriptional activity in cells**

The main evidence for this finding is presented in **Fig. 1**, which provides details of the massively parallel reporter assay (MPRA) designs, experiments, and data analyses used in this study (**Fig. 1a, b**) and utilizes the results from STARR-seq experiments using the TF motif library (motif-STARR-seq; **Fig. 1c, d**) to analyze enhancer activity in human cells. In the STARR-seq experiments, the active enhancer elements can be identified by transfecting cells with a plasmid library in which the DNA fragments of interest are cloned downstream of a reporter gene, and by analyzing the reporter-specific RNA to identify self-transcribed active elements (**Fig. 1b**).

The rationale for designing the TF motif library for STARR-seq experiments was to systematically test the enhancer activity of known TF motifs in isolation (one copy) and in multiple copies (**Fig. 1a**, i). Thus, the motifs were placed in two motif-depleted sequence contexts in different orientations and spacings. The motifs used in the design were collected from previous HT-SELEX studies as described in **Supplementary Methods** (section **Motif collection**). HT-SELEX is an *in vitro* assay for determining the DNA binding specificities of individual TFs through systematic evolution of ligands by exponential enrichment, and a total of 1121 HT-SELEX-derived motifs were included in the TF motif library design in this study. In addition, a degenerate base N was added at every position of each motif one position at a time to generate in total 30,123 mutant consensus sequences. The motif library comprises a total of 92,918 individual sequence patterns including these 1121 TF consensus sequences and 30,123 mutant consensus sequences in two motif-depleted sequence contexts (to control for effect of flanking bases). Each pattern only contained one type of consensus sequence, present once, twice and/or three times. Multiple consensus sequences were arranged in different spacings and orientations relative to each other. In the figures, dimeric motifs are indicated by orientation with respect to core consensus sequence (GGAA for ETS, ACAA for SOX, AACCGG for GRHL and GAAA for IRF; HH head-to-head, HT head-to-tail, TT tail-to-tail, followed by gap length between the core sequences). **Supplementary Table 5** describes the naming of the motifs in each figure.

The main advantage of this approach is that it enables measuring of the functional enhancer activity of distinct *in vitro*-derived TF motifs *in vivo* in human cells. Based on the MPRA experiments, we identified the most active motifs in GP5d cells (**Fig. 1c**). The most active motifs displayed comparable activities when placed in different sequence contexts (**Extended Data Fig. 1a**), and their relative activities were consistent across experiments using two different CpG-free basal promoters, δ1-crystallin and EF1α promoters (**Extended Data Fig. 1b**). The mutated TF motif patterns enabled generation of activity-based position weight matrices (PWM) for the TFs (**Fig. 1d** and **Extended Data Fig. 1d**). So far, the PWMs have been generated either on the basis of the *in vitro* TF-binding specificities from the SELEX experiments or as enrichment of sequence features within ChIP-seq peaks for a particular TF. Compared to these previous approaches, our results provide a direct measurement of enhancer activity of each consensus motif and its mutated variations in human cells.

The main limitation of the motif-STARR-seq experiment stems from the facts that many TFs bind to highly similar motifs and the binding specificities for all individual TFs are not yet known. However, although the assay measures activities of consensus sequences and/or motifs, the TFs or groups of TFs that bind to the sequences can in most cases be inferred from the specific motifs and TF expression levels[3]. We used RNA-seq data to determine the expression levels of the TF families with strongest detected TF motif activity, as shown in

Extended Data Fig. 1c. Such combined analysis revealed that TFs that are expressed at a relatively high level in GP5d cells can bind to the strongly active motifs (average 102 transcripts per million (tpm) vs. 26 tpm for all genes and 20 tpm for all TFs from ref. [4]); however, the correlation between motif activity and expression of corresponding TFs was weak **(Fig. 1c**, **Extended Data Fig. 1a-c**). This is likely caused by the facts that: 1) different TFs have different binding energies, 2) different TFs have different transcriptional activator activities, 3) mRNA expression level does not fully determine protein expression level, and 4) mapping of motifs to TFs is not perfect. Since all expressed TFs that are capable of transactivating through similar sequences can contribute to the measured enhancer activity, assigning the measured enhancer activity to a specific TF is not always possible. To acknowledge this limitation in our study, in the figures the motifs have been named according to the TF class or subclass for the cases where HT-SELEX motifs for several TFs are highly similar. Same principle has been applied also when specificities of closely related TFs have not been measured yet but can reasonably be expected to be similar based on the sequence homology.

Another potential limitation of the MPRA approach could be a strong activation of cellular alarm responses due to experimental conditions, as described previously (see ref. [1]). Based on the motif-STARR-seq data, only three other motifs, representing interferon regulatory factor (IRF), grainy-head like (GRHL) and E26 transformation-specific (ETS) TFs had activity that was within 1% of the maximal activity observed for the p53 family motif (**Fig. 1c,** dotted line). Since the two most enriched motifs, p53 and IRF, are bound by TFs that respond to cellular alarm signals such as DNA damage (p53) and cytoplasmic DNA (IRF), we studied the extent of these alarm signals in our experimental system by performing control experiments in which the HepG2 cells were transfected with the genomic STARR-seq library or treated with 5-fluorouracil (5-FU) to induce p53. Cellular responses were analyzed by RNA-seq for gene expression, ATAC-seq for chromatin accessibility, and ChIP-seq for p53 binding as well as for active histone mark H3K27ac. Importantly, we found that plasmid library transfection itself caused very minor changes in the cellular transcriptome (**Supplementary Fig. 1c**) or p53 binding, chromatin accessibility and H3K27 acetylation at p53 and IRF target genes (**Supplementary Fig. 1f**). Gene set enrichment analysis showed that p53 target genes were strongly enriched among differentially expressed genes in 5-FU-treated cells, but not in cells transfected with the STARR-seq plasmid (**Supplementary Fig. 1d,e**). These results indicate that in HepG2 cells, the alarm response is mild.

Of note, in all the STARR-seq experiments in this manuscript, the cells were in an unstimulated state. Thus, the activity of some TFs that are active only upon stimulation (for example, hormone- and growth-factor dependent TFs, immune response-related TFs), is not detected under the experimental conditions used.

2) ***De novo*** **enhancers display weak TF spacing and orientation preferences**

Fig. 2 utilizes the results from STARR-seq experiments with TF motif library (**Fig. 2a,b**) and random enhancer library (**Fig. 2c-f**) to analyze enhancer activity in human cells. In addition, active TF identification (ATI) assay for measuring DNA binding activity of the TFs (**Fig. 2a**) in the same cell line in which the motif-STARR-seq was performed.

The rationale for comparing the motif-STARR-seq results to ATI results was to study how the enhancer activities of TF motifs correlate to the DNA binding activities of the respective TFs. ATI assay utilizes a library of random DNA sequences that are incubated with nuclear extract from the cell type of interest, and the oligonucleotides that are bound by the TFs are enriched using electrophoretic mobility shift assay[3]. Comparison of TF motif activity from motif-STARR-seq to TF binding activity from ATI assay revealed only weak correlation between the transcriptional activity and the biochemical activity (R=0.032; **Fig. 2a**), indicating

that the TF motifs responsible for the activities are largely distinct. Note also that some TFs can bind to two or more slightly different motifs that can have somewhat different activities (e.g. p53-family motif is bound by all p53 family members, p53, p63 and p73, whereas the p53-specific motif is only bound by p53. Although there are limitations in comparing the results from two different assays, such as different technical properties and sensitivity of each assay, as well as quality of the individual data sets, the weak correlation we observed also reflects the different aspects of TF function measured by these two assays; ATI assay enriches the DNA sequences that are most strongly bound by the TFs expressed in a particular cell type, whereas STARR-seq measures the outcome of enhancer activity as accumulation of specific transcripts in response to TF binding. Our results demonstrate that the TFs with strongest DNA binding activity in a cell are largely different from the strongest transcriptional activators. Moreover, the two strongest binders identified by the ATI assay in GP5d cells (NFI and NRF1) were also reported as strong binders across many human tissues in a previous ATI study[3], suggesting that strong binding does not necessarily lead to strong cell type-specific transcriptional activity.

Since the synthetic motif library contains sequences with different copy numbers for each motif, we were able to measure the effect of motif copy number on enhancer activity. For the majority of the TFs, we observed an additive effect as a function of the number of consensus sequences (**Extended Data Fig. 1e**, red horizontal lines), whereas for the strong activators, multiple copies of the motifs result in saturation (**Fig. 2b**). For each motif, fold-change ($\log_2$) compared to input is shown for one versus two sites. Of note, additive effect in the logarithmic space equals multiplication of fold-changes.

In addition to the TF motif library (**Fig. 1a**, i), we used a library comprising synthetic random 170 bp sequences (**Fig. 1a**, iii) cloned downstream of the reporter gene in the STARR-seq experiments. These two approaches complement each other: while TF motif library enables studying the enhancer activity of different variations of predesigned sequence patterns, the experiment using the random library is free from any *a priori* assumptions of what constitutes an active enhancer in human cells. Active motifs identified in the experiments using these two different library types were highly similar (**Fig. 1c**; **Extended Data Fig. 2b**), illustrating the robustness of the assay. Furthermore, comparison of replicate experiments for random enhancer libraries revealed high reproducibility across different $\log_2$ fold-change values (**Extended Data Fig. 2a**), demonstrating the high dynamic range of the assay. Note that while p53 and IRF are the strongest activators, also colon cancer-specific TF activity can be reliably measured from the random enhancer library. These results show that regulatory elements can readily be generated in an experiment by selecting sequences from a very high complexity library, although not all such elements would be optimized or as highly active as the most active elements in the genome. Furthermore, the active motifs enriched from the random enhancer library were also largely similar between the two different cell types, GP5d and HepG2 (**Fig. 2f**), and only few TFs were found to be specific for each cell type. These include for example GRHL in GP5d, TEAD and ATF4:CEBPB in HepG2, and TFs that correspond to the known deficiency of the HepG2 cells in interferon signaling (IRF3)[5].

The random enhancer library also enabled studying the effect of motif spacings within the active sequences. In general, the spacing and orientation preferences between TFs were weak, but we observed few specific motif pairs with significantly overrepresented spacing preferences (**Fig. 2c**). The sequence logos shown in **Fig. 2c** are the most enriched spacing and orientation for each pair according to P value; fold-change is its count compared to average of all others, and the adjusted P value is calculated by comparing it to all others (one-sided Fisher's exact test) and correcting for the total number of orientations and spacings tested for the pair (see **Supplementary Methods**; **Supplementary Table 5**). Since the number of detected motif pairs with spacing preferences was small, we wanted to estimate whether the statistical power of our assay was sufficient for detecting specific spacings and orientations. We conducted a

power analysis that simulates different effect sizes and motif match thresholds with the same data size as was used in the motif spacing analysis shown in **Fig. 2c**. The analysis showed that the assay has sufficient power to detect moderate effects (fold-change ≥ 3 if 1% of the pairs had a specific interaction) with high probability (> 0.77) using the same motif match threshold as in the figure (10^{-4}) which is consistent with the interactions shown. Moreover, even with a more stringent threshold (6×10^{-5}) that corresponds approximately to a motif with information content of 15 bits typical for TFs, the assay should be able to detect fold-changes ≥ 5 (probability > 0.92), but only p53/p63 motif interactions were detected with this threshold. Thus, although it is difficult to rule out that some specific interactions could have been missed, the power analysis indicates that if strong specific interactions existed between individually enriched motifs in the assay, we would have detected them with very high likelihood.

In addition, we analyzed the motif spacing from mean of mutual information between 3-mer distributions plotted as a function of distance separating the 3-mers in GP5d random enhancers (**Extended Data Fig. 2e**) and binary STARR-seq enhancers (**Extended Data Fig. 2f**) from the library in which both the promoters and the enhancers comprise synthetic random sequences (**Fig. 1a**, iv). We observed that pairwise dependency between 3-mer distributions measured with mutual information varies with a period of approximately 10 bp and decays as a function of distance separating the 3-mers, indicating that most of the dependencies in the enhancers are short-ranged (<50 bp).

In addition to motif matching, we analyzed the random enhancer STARR-seq sequences using different machine-learning models in order to understand what features are needed to separate sequences acting as active enhancers from inactive sequences (**Fig. 2d, e**). Data from the random STARR-seq experiments is well suited for machine-learning analyses for three main reasons: 1) In the STARR-seq experiment, the transcriptional machinery of the cell acts as a filter that lets through sequences that can drive transcription. Thus, the experiment can be naturally modeled as binary classification where we aim at finding the features that separate the active sequences from the inactive ones. 2) Background model of the random STARR-seq experiments is much simpler than that of the genome; for example, synthetic random sequences lack the background from mutational processes not directly related to transcription. 3) Number of distinct training samples available is high, *e.g.* ~9.2 million sequences for the GP5d random enhancer STARR-seq (see **Supplementary Table 7**).

We started with simple logistic regression that uses a set of 880 previously published TF binding motifs as features (**Fig. 2d**). This model is capable of explaining the activities of the STARR-seq enhancers with a linear combination of effects of known individual TF binding motifs (see section **Logistic regression classification** in **Supplementary Methods** for details). This model performed similarly in classification to a model that was trained using the 22 *de novo* motifs discovered from the random enhancer STARR-seq data (see **Supplementary Fig. 2a,b**), indicating that the L1-regularization and the used training scheme were capable of finding the correct active features. We then created a pairwise logistic regression model (see **Supplementary Methods**), that uses in addition to the 880 individual TF motifs terms that account for cooperative pairwise binding of each TF with itself and the top 20 most important TFs (according to the regression coefficient absolute values from the simple logistic regression) with all other TFs from the set of 880 binding motifs (in total 19,150 features). All possible pairings could not be tested due to computer memory requirements, but we reasoned that it would be very unlikely for individually inactive motifs to have very important cooperative interactions. Nevertheless, we also trained convolutional neural network (CNN) models similar to recent successful approaches, used for example in refs [6,7].

One strength of the CNN approach is that it learns the important predictive features from the data allowing, in principle, learning any kind of sequence features. In our design of the random enhancer STARR-seq CNN, the first layer filter size was optimized between 5 and

9 (see **Supplementary Table 7** and section **Convolutional neural network classification** in **Supplementary Methods** for details), and the number of filters in the first layer of the CNN models was capped at 256, which with the minimum dropout of 0.3 allows the models to learn approximately 180 different 5 or 9 bp long filters representing binding motifs. The subsequent dilated convolutional layers (up to 7 layers in total) can then learn to combine these filters into longer motif representations and interactions between them. The limited number of strongly active TF binding motifs detected using motif matching (**Fig. 2b**), *de novo* motif mining (**Extended Data Fig. 2d**; **Supplementary Fig. 2a**) or logistic regression **(Fig. 2d**) analyses strongly suggest that this limitation of the CNN model size is reasonable. The CNN classifier performed better in classifying the GP5d random enhancer data than the logistic regression or gapped k-mer SVM models (see section **Note on optimal classification of random enhancer STARR-seq data** in **Supplementary Methods** for more discussion).

A clear benefit of the logistic regression approach is that the importance of each designed feature is straightforward to read from the regression coefficient values (**Fig. 2d**). With CNN models, interpreting the features learned by the model and their importance towards the classification is not as straightforward, although considerable progress has recently been achieved (e.g. refs [6,7,8]). We used a barrage of different interpretation strategies to inspect the features learned by the random enhancer CNN model (**Fig. 2e**; **Supplementary Fig. 2c**; **Extended Data Fig. 3c**; **Extended Data Fig. 4).** First, we observed that if we create "artificial enhancers" by embedding one or two instances of known binding motifs into inactive input library sequences (**Fig. 2e**), the enhancer probabilities predicted by the random enhancer STARR-seq CNN agree well with the results from the motif matching (**Fig. 2b**) and logistic regression (**Fig. 2d**) analyses. This shows that the CNN has learned similar effects for the known TF binding motifs than the logistic regression model. The core sequences of the motif features used by the logistic regression model were typically also learned by the CNN, but the weaker flanking bases of the motifs were more variable (**Extended Data Fig. 3c**). Other types of sequence features, or interactions between features (**Fig. 2e**) might also exist that are important in classification but not captured by the logistic regression model (see **Supplementary Methods**). Moreover, *de novo* motifs from unseen *in silico*-generated random sequences classified as active enhancers by the CNN model are highly similar to *de novo* motifs from the random enhancer STARR-seq (**Supplementary Fig. 2c**). Of note, CNN also identifies similar features than the enrichment analysis shown in **Extended Data Fig. 2d**.

Re-creating the important binding motifs from the logistic regression model using the per-base importance learned by the CNN (**Extended Data Fig. 3c**, see section **Interpretation of convolutional neural network classifiers** in **Supplementary Methods**) revealed that most of the motifs learned by the CNN were different from the HT-SELEX derived motifs at the less defined positions (*e.g.* ELF5, SP1), for some motifs, only part of the HT-SELEX motif was used by the CNN (*e.g.* TCF7, TP63), and some motifs seemed not to have been learned at all (*e.g.* NFAT5, SMAD3). TF-MoDISco[9] analysis further supported this by recovering only part of the motifs used by the logistic regression classifier (**Extended Data Fig. 4**). The machine-learning classifiers are regularized and trained so that they aim at finding simple features that generalize well to new datasets. The logistic regression models were regularized using L1 norm and the CNN models with dropout layers. Thus, it is not surprising that the less information-rich parts of TF binding motifs are not necessarily deemed important for the classification task. This does not necessarily mean that they are not important for biology and care must be taken when interpreting the features used by machine-learning models, since the partial binding motif might be as good for classification as the full-length motif according to the used validation data, so it is not necessary to learn the more complex full-length motif.

3) Methylation status affects enhancer activity both positively and negatively

Analysis of the role of CpG methylation on enhancer activity is shown in **Fig. 3**. In addition, **Fig. 3** shows the RNA-sequencing data that was used for analyzing cell type-specific expression of TFs (**Fig. 3a**). Genomic STARR-seq was used for studying the effect of CpG methylation on enhancer activity in GP5d cells (**Fig. 3b-d**). Furthermore, previously published bisulfite sequencing data from GP5d cells (from ref. [10]) was used for detecting genome-wide CpG methylation at the endogenous genomic loci (**Fig. 3b**) and ATAC-seq was used for mapping the accessible chromatin regions in the genome so that open and closed chromatin regions could be correlated with genomic STARR-seq activity in GP5d and HepG2 cells (**Fig. 3b, e**). Genomic STARR-seq experiments were also performed in *TP53*-null GP5d cells (**Fig. 3b**).

To enable analysis of the effect of DNA methylation on transcriptional activity, we developed a MPRA vector that is devoid of CG dinucleotides: in designs i to iii, the Lucia reporter gene is driven by CG depleted minimal δ1-crystallin and EF1α promoters, whereas in design iv, the promoter is replaced by 150 bp random DNA sequences (see **Fig. 1a, b**; **Methods** and **Supplementary Tables 1, 2**). Since the vector backbone is devoid of any CG nucleotides, *in vitro* methylation of the plasmid library only methylates the DNA fragments of interest. The enhancer activity of methylated and unmethylated genomic libraries can then be analyzed in parallel transfections. In the genomic STARR-seq experiments using methylated and unmethylated libraries, we did not observe a very strong overall effect from DNA methylation on the landscape of enhancer activity (**Fig. 3b**). However, the activities of individual enhancers were affected in both directions; we also found differential enrichment for specific TF motifs containing CG dinucleotides that were consistent with the reported binding specificities for methyl-plus and methyl-minus TFs (**Fig. 3d**; see ref. [10]). These results demonstrate that differences in TF activity in the presence and absence of DNA methylation can be detected using a genomic STARR-seq assay.

4) Genomic analysis reveals three types of transcriptionally active enhancers

Genomic enhancer activities were compared with chromatin features in **Fig. 4**, which shows the results from STARR-seq experiments with genomic DNA library in two cell lines, GP5d and HepG2, as well as analysis of chromatin accessibility using ATAC-seq and TF binding and epigenetic features using ChIP-seq in the same cell lines (**Fig. 4a, b**). This analysis revealed six types of gene regulatory elements (**Fig. 4c**).

The rationale for using the genomic DNA library was to study the enhancer activity of sequence features in their native genomic sequence context. In contrast to rationally designed and randomly synthesized DNA fragments used in the STARR-seq experiments in **Fig. 1** and **Fig. 2**, the genomic DNA library comprises ~500 bp fragments of the human genome cloned downstream of the reporter gene (**Fig. 1**, ii). The active enhancers were identified by calling the peaks from the STARR-seq-enriched RNA fragments against the plasmid input sample using MACS2[11] (see **Methods**). The high complexity of the library (2.09×10^9 distinct clones; **Supplementary Fig. 1a,b**), replicate concordance and excellent signal-to-noise ratio allowed detection of enhancer activity at ~1.5 bp resolution (**Extended Data Fig. 5a**, **b**; see **Methods**). The genome browser snapshot in **Extended Data Fig. 5b** shows a near base-pair resolution at the STARR-seq peaks, indicating that active fragments drive gene expression and are converted to cDNA, recovered and sequenced at a high probability, speaking against any potential bottleneck-effects or undersampling. Also note that cell type-specific STARR-seq signals agree with tissue-specific TF binding (highlighted with pink boxes in **Extended Data Fig. 5b**): TP53 binds at intronic enhancer in both GP5d and HepG2 cells, but stronger STARR-seq signal in HepG2 cells is observed at the enhancer upstream of the *BBC3* gene that co-localizes with specific HNF4A binding in HepG2. Since the active enhancers identified by the genomic

STARR-seq experiments can be mapped to their original loci in the human genome, the data can be compared with other functional genomic data sets. Here, we have used ATAC-seq data sets to identify open, transposase-accessible regions in the chromatin, and ChIP-seq to map epigenetic features associated with enhancer activity (such as H3K27ac) and repressed chromatin (such as H3K9me3 and H3K27me3). In addition, ChIP-seq data for CTCF is used for identifying structural chromatin elements such as boundaries of regulatory units. Of note, there is much higher overlap between the transcriptional activator TFs p53 and FOXA1 ChIP-seq and STARR-seq than between the architectural chromatin protein CTCF and STARR-seq (**Extended Data Fig. 5c**).

The strength of the approach utilized in **Fig. 4** is in combining different, complementary data sets to classify human gene regulatory elements. Traditionally, open chromatin sites have been considered to contain active gene regulatory elements, and ATAC-seq can identify both active promoters and enhancers. However, chromatin openness is an indirect measure for activity, and the functional consequence of the elements identified using ATAC-seq needs to be validated using other methods. Genomic STARR-seq, on the other hand, measures the functional enhancer activity of genomic sequences in an unbiased manner in an episomal system. We performed predictive modeling using Lasso regression to test whether these datasets can predict differences in gene expression. This analysis clearly shows that ATAC-seq and STARR-seq both independently predict differential gene expression, and thus we utilized these data sets to classify human gene regulatory elements. This analysis revealed three different types of active enhancers: closed chromatin enhancers (STARR-seq only), classical enhancers (both signals) and chromatin-dependent enhancers (ATAC-seq only).

We define six classes of gene regulatory elements based on the integrative analysis of different functional genomics data sets (**Fig. 4a-c**). There are previous reports that have classified enhancers for example as “active”, “primed”, and “poised” (see, *e.g.* ref. [12]). This classification did not consider the functional enhancer activity measured by a reporter assay, which gives an additional layer of information that is used for classification in this manuscript (**Fig. 4c**). However, the different classifications are partly overlapping. Here, we define cryptic enhancers as STARR-seq+, ATAC-seq- sites that show enrichment for H3K9me3 and/or H3K27me3 repressive chromatin marks. Thus, these resemble previously reported class of “poised” enhancers that are characterized by H3K27me3[12]. As shown in **Fig. 4a, c**, both classical and chromatin-dependent enhancers are characterized by a strong H3K27ac signal. At closed chromatin enhancers the H3K27ac mark is weaker, whereas TF binding is clearly observed to these genomic loci and enhancer activity is detected using STARR-seq (for example STARR-seq signal overlapping with p53 binding at the intron of the *RRM2B* gene in both GP5d and HepG2 cells; **Fig. 4a**). STARR-seq peaks in closed chromatin represent two element classes: closed chromatin enhancers (no repressive histone marks) and cryptic enhancers (overlap with repressive histone marks such as H3K9me3; see **Fig. 4a**). Overlap analysis revealed that cryptic enhancers do not show overlap with the H3K4me1 mark that is considered to mark the “primed” enhancers, but around one fourth of the closed chromatin enhancers are enriched for H3K4me1 (**Extended Data Fig. 5d**). However, since primed enhancers are considered to be located in open chromatin (see ref. [12]), closed enhancers do not fully represent the features of “primed” enhancers, but rather represent a distinct category of enhancers.

STARR-seq experiments are limited by the complexity of the input library. In our case, the genomic library complexity is extremely high with estimated 2.09×10^9 distinct fragments covering the human genome (3.2×10^9 bp) with 1.53 bp resolution on average. This provides unprecedented power to detect active enhancer elements in the human genome. A potential risk associated with high library complexity would be undersampling of the input library. However, we designed the experiments carefully to avoid any bottleneck-effects and did not observe any

evidence of undersampling. This is based on analysis of peaks in the genomic library, which would have low resolution if many clones were lost during the experiment and only some would be recovered despite all elements being active. By contrast, we see near base-pair resolution at the peaks, indicating that active fragments drive gene expression and are converted to cDNA, recovered, and sequenced at a high probability (see genome browser snapshots in **Fig. 4a, b**; **Extended Data Fig. 5b**).

As shown in **Fig 2f**, p53 family motif is the strongest activator in both GP5d and HepG2 cells, having the largest fold-change of motif match count over input in each cell line. Similarly, IRF motifs also show strong activity in GP5d cells. We used the genomic STARR-seq data to determine what fraction of all detected elements are regulated by p53 by performing overlap analysis between genomic STARR-seq peaks and the ChIP-seq peaks for TP53. This analysis revealed that p53 is not responsible for most of the enhancer activity detected using STARR-seq. Specifically, 16% and 4.9% of the genomic STARR-seq peaks overlap with a p53 ChIP-seq peak in GP5d and HepG2 cells, respectively (**Extended Data Fig. 5c,d**). Also, only a small fraction of the STARR-seq peaks harbor a p53 family or IRF3 motif (**Extended Data Fig. 5c,d**). These results demonstrate that although p53 family and IRF were the most enriched motifs, the large majority of active enhancers did not contain these elements despite these motifs being the strongest activator in the cells, suggesting that the gene regulatory system of the cell is not dominated by these cellular alarms.

5) Cell type-specific gene expression is driven by few lineage-specific transcription factors; there are promoter-specific sequence features, but no motif enriches specifically at enhancers

Fig. 5 utilizes a binary STARR-seq design in which both promoter and enhancer elements comprise of random synthetic sequences. This design enables measuring the sequence features that contribute to enhancer and promoter activities separately, and also detection of promoter-enhancer interactions. In **Fig. 5a,** motifs enriched at random promoters in three different cell lines were analyzed, and in **Fig. 5b, c** we studied the similarities and differences between motifs enriched at active promoters and enhancers.

The rationale for using DNA libraries comprising synthetic random sequences is to study the determinants of active regulatory elements in an unbiased manner, independently of their sequence context. In the experiments, random sequences that contain sequence elements that can contribute to promoter or enhancer activity in a given cell are enriched without being limited by pre-designed parameters. Ultra-complex random libraries also enable studying far larger sequence space than the entire human genome. While the human genome is too short to encode all possible sequence combinations for TF binding elements, the random library can accommodate a substantially larger number of sequence combinations with potential regulatory activity.

The size of the ultra-complex DNA library prohibits precise determination of initial input frequencies of individual sequences. Thus, our analysis relies on finding common features of different selected sequences instead of their counts. In fact, analyzing enriched features of distinct otherwise random sequences is a very powerful way to distinguish the selected features from the rest of the sequence; it also prevents high copy-number or repeat sequences from biasing motif-mining or motif-spacing analyses. For example, to find enriched TF motifs we counted the motif matches separately in input DNA library and STARR-seq RNA library taking into account the mononucleotide frequencies in each and thus for example differing GC-contents did not affect the enrichment analyses (see **Methods** for details). This approach is not limited to or biased towards assumed motifs, also allowing *de novo* motif analysis or any other sequence feature enrichment analysis.

One of the limitations of many MPRA approaches is that they are less sensitive for detecting repressive effects than enhancing effects due to the minimal promoter typically used in the constructs. We could, however, detect some repressive motifs such as OVOL and CUX (**Fig. 5b**). These results demonstrate that also repressive features can be analyzed using our approach, although with lower sensitivity.

6) TFs can have enhancing, promoting and/or TSS-defining activities

Fig. 6 shows the data generated from a binary STARR-seq experiment in which both promoter and enhancer elements consist of random synthetic sequences. Here, the focus is on the detailed analysis of the positional specificity of sequence motifs at active promoters. Template switch strategy was used for determining the exact position of the TSS within the random promoter sequence (**Fig. 6a**). This enables studying the enrichment and precise position of the sequence features upstream and downstream of the TSS. Cartoon in **Fig. 6a** shows the design of the template-switch chemistry used to capture the 5' sequence of the transcribed RNA using a template switch oligo (TSO) to determine the precise location of the transcription start site (TSS) within the random DNA sequences cloned at the place of the promoter. The sequencing libraries are generated by reverse-transcribing the reporter-specific RNA to cDNA using a reporter-specific primer (orange) and a TSO containing a unique molecular identifier (UMI; brown) and a 5' sequencing adapter (turquoise/green), and by PCR-amplifying the cDNA utilizing primers that bind to the adapters to introduce the Illumina linkers (red) to the final library (3' adapter sequence marked with dark blue is present in the reporter construct).

One of the strengths of our approach in analyzing random sequences is that the enrichment of sequence features can be analyzed independently of their sequence context. Analysis of the sequence features within the random promoters revealed positional preferences for several features, including AT-rich sequence (TATA box) upstream of the TSS and a novel G-rich sequence downstream of the TSS; these elements showed mutually exclusive interaction with the TSS (**Fig. 6b, c**). Many TF motifs preferentially enriched close to the TSS; on average, $\log_2$ fold-change vs. input values for the 50 most enriched TF motifs were 53% higher in the region from -51 to TSS compared to region -100 to -50 **(Fig. 6d**). After identifying the novel G-rich sequence from the random promoters, we then could also detect it from endogenous genomic sequences downstream of TSS (**Supplementary Fig. 3c**); to our knowledge it has not been recognized before, potentially because the feature might be obscured by the general GC anisotropy (more guanines on the plus strand) both upstream and downstream of the TSSs[2]. We also observed that the reads obtained from the template switch analysis preferentially mapped to positions that displayed a 10 bp periodicity relative to the STARR-seq vector (**Supplementary Fig. 3a**). The pattern was consistent with the loading of the RNA polymerase II pre-initiation complex onto the random sequence, as the periodic pattern weakened when the ~100 bp region occupied by the pol II complex included plasmid-derived constant sequences. Similar, but very weak periodicity was also observed in p53 motif positioning at the enhancer, suggesting that plasmid supercoiling, or some sequence feature in the vector makes one side of the DNA more accessible (**Supplementary Fig. 3b**).

We used a convolutional neural network classifier (CNN) trained on the random synthetic STARR-seq promoters to score known variants at the *TERT* promoter[13,14,15,16] to assess how well a model trained on synthetic STARR-seq sequences has learned to predict the effects of mutations in human regulatory sequences. As shown in **Fig. 6f**, CNN predictor correctly identifies cancer-associated mutations in the *TERT* promoter[16]. Of note, the predictor identifies the ETS motifs that are generated by the driver mutations and the predicted promoter probabilities ($P_{promoter}$) are higher for the mutant promoters. The model predicted a higher promoter probability for all but one of the mutant promoters (p61:C>T) compared to the *TERT*

wild type promoter, predicting that 14 out of 15 known *TERT* promoter activating variants[15] increase gene expression (**Extended Data Fig. 8a**). The model predictions also correlated well with previously published saturation mutagenesis MPRA experiments of the *TERT* promoter (**Extended Data Fig. 8b-d**), even when the CNN was trained for classification, not for regression. Training of a conventional regression model is not possible using the STARR-seq data as the size of the ultra-complex DNA library prohibits precise determination of initial input frequencies of individual sequences. However, the saturation mutagenesis experiments were carried out in different cell lines than GP5d for which our model was trained, but the correlation between the predicted and the measured effects of the variants was still close to the correlation observed between two experimental MPRA datasets using different cell lines.

Next, we used the CNN trained on the STARR-seq promoter sequences to predict the positions of active transcription start sites in the GP5d cells, as measured using CAGE (see **Methods**). The predictions were compared against a position-specific logistic regression model (see section **Position-specific logistic regression classification** in **Supplementary Methods**) of the STARR-seq promoters as well as against PWM and CNN models trained on the experimentally validated human genomic promoters downloaded from the Eukaryotic Promoter Database. For each model, we predicted the TSS probability by scoring each 120 bp long sequence within 500 bp from each of the active GP5d TSSs in the unseen test set. For each model and each test set TSS, the position giving the highest TSS probability was chosen as the predicted TSS position, meaning that for each model, one final TSS position prediction was made per each test set TSS. The rationale for this experiment was to see how well the models have learned features that determine the position of the TSS. As shown in **Fig. 6g**, CNN trained on random promoter data outperforms PWM-based models, regression models, and CNN trained on genomic promoter data in predicting active TSS positions in GP5d cells. The TSS probability of each position within ±500 bp from the known TSS position was evaluated for each model, and the most likely TSS position was used as the predicted TSS. The score shown in **Fig. 6g** indicates the fraction of predicted TSS positions falling within ±25 bp from the annotated TSS positions in the genome for each model separately.

Models trained on the STARR-seq promoters predict the TSS position in the genome more accurately than similar models trained on the genome itself, indicating that the training data with a better controlled background and more samples can allow learning more accurate models of gene regulatory elements (see also **Extended Data Fig. 8e**). Moreover, the STARR-seq CNN model outperformed the STARR-seq position specific logistic regression that uses as features the position-specific enrichment of the known TF binding motifs and promoter-specific features (such as TATA-box and Initiator, see **Supplementary Table 12**) in the training data, both in TSS position prediction (**Fig. 6g**) and slightly in classification (**Supplementary Fig. 4**). This indicates that similarly to the random enhancer CNN, the promoter capture CNN has also improved the motifs for better classification.

Mutual information (MI) analysis shown in **Extended Data Fig. 8f,g** visualizes the pairwise dependencies between position-specific 3-mer distributions learned by the STARR-seq promoter CNN and the genomic promoter CNN models, respectively (see section **Interpretation of convolutional neural network classifiers** in **Supplementary Methods** for details). These figures were generated from sequences that each of the models predict to represent highly active promoters, and thus they visualize the interaction patterns that each of the models looks for in sequences they deem as highly active promoters. The genomic promoter CNN mostly uses information right at the TSS (position 100 in **Extended Data Fig. 8f,g**), while the STARR-seq promoter CNN has learned a pattern that includes two other MI peaks before the TSS which is likely helping in discriminating between real active TSSs and other sequences in the genome.

The number of 5 bp long filters in the first layer of the CNN models was capped at 256, which with the minimum dropout of 0.3 allows the models to learn approximately 180 different 5 bp long filters representing binding motifs. These 5 bp long motifs can then be combined together to form representations of longer motifs (either continuous or gapped) by the subsequent layers of the network. In light of the motif matching analyses that show strong activity for only a limited number of motifs (**Fig. 5b, Fig. 6d**), and the good classification performance of the promoter CNN models (**Supplementary Fig. 4**), this size limitation of the CNN models is reasonable.

7) Enhancer-promoter interactions are additive and non-specific in nature

Fig. 7 utilizes the data generated from a binary STARR-seq experiment in which both promoter and enhancer elements comprise of random synthetic sequences. Here, the focus is on analyzing the interactions between promoters and enhancers in an unbiased manner.

We set out to test if a deep learning model is able to learn information that helps in classifying sequence pairs to active and inactive promoter-enhancer pairs from the specific pairing of the active promoter and enhancer sequences in the binary STARR-seq experiment. As machine-learning model performance is dependent on the model architecture and the hyperparameters, we planned an *in-silico* experiment where exactly the same kind of a convolutional neural network (CNN) model was trained on differently permutated datasets from the binary STARR-seq experiment. The CNN model consisted of two convolutional input bodies, one of which reads in the promoter sequence and the other the enhancer sequence. The number of dilated convolutional layers per body was optimized during training as described in the **Methods**. Before the prediction verdict, the information from these separate convolutional bodies was integrated with a fully connected layer to enable learning interactions between features in the promoter and the enhancer parts of the binary STARR-seq data.

In this *in silico* experiment, the original pairing from the binary STARR-seq experiment should contain information about any specific interactions between the specific pairing of promoter and enhancer sequences observed to be active in the STARR-seq experiment (denoted as "paired" in **Fig. 7c**). We then permutated the pairing of active promoters and enhancers so that each active promoter was still paired with an active enhancer, but with a different enhancer than in the original data (denoted as "permutated" in **Fig. 7c**). This permutation removes information about specific interactions in the promoter and enhancer pairs. Permutating the pairings between the promoters and enhancers resulted in similar performance (paired Student's t-test two-sided P value =0.134 for comparison of AUprc from "permuted" CNN to "paired" CNN across the tested hyperparameters; **Fig. 7c**), indicating the absence of specific interactions between promoters and enhancers in our binary STARR-seq experiment.

In the "enhancer from input" and "promoter from input" training sets, the promoters and enhancers, respectively, were paired with a randomly sampled inactive sequence from the input library. In the "enhancer from input" model, the classification is purely based on the promoter features and in the "promoter from input" purely on enhancer features. We observed that both the promoter and the enhancer sequences contain information that is helpful in the classification task. When active promoter sequences were paired with inactive sequences from the input library (denoted as "enhancer from input" in **Fig. 7c**), the classification performance of the CNN model dropped significantly. An even clearer drop in classification performance was observed when active enhancers were paired with inactive sequences from the input library (denoted as "promoter from input" in **Fig. 7c**).

Supplementary Methods

STARR-seq reporter library construction and cloning

TF motif input DNA library

A pool of 92,918 oligos with a length of 79 nucleotides (nt) was designed with a 49-nt variable region and two 15-nt flanking regions with constant sequences for the library cloning (see Motif library design for more details), and synthesized by CustomArray Inc. The oligo pool was prepared for cloning in a two-step protocol using Phusion DNA polymerase (Thermo Fisher) and Oligos 1 and 2. Frist, 2.5 pmol of the oligo pool was double-stranded using 100 pmol of Oligo 2 in two parallel reactions [98 C for 3 minutes (min), followed by 5 cycles of 98 C for 10 seconds (s), 55 C for 15 s, 72 C for 15 s, and the final extension at 72 C for 2 min]. The two reactions were then split into ten reactions and after adding 10 pmol of the Oligo 1 to the reactions the PCR was performed for ten additional cycles using the same conditions. Ten PCR reactions were pooled and the 127-bp product was gel-purified. The pGL4.10-Sasaki-SS (a) and pCpG-free- EF1α-SS (b) vectors were linearized by digestion with AgeI and SalI for 3 h at 37 C and gel-purified. For each backbone, the In-Fusion cloning (Clonetech) was performed in 20 reactions using 200 ng of linearized vector and 50 ng of double-stranded oligo pool according to the manufacturer's instructions. Five In-Fusion reactions were pooled and purified using MinElute PCR purification columns (Qiagen) and eluted in 12.5 ul nuclease-free water per column. The bacterial transformation was done by electroporation using Gene Pulser Xcell (Biorad) in 20 parallel reactions with 2.5 ul of purified eluate and 20 ul of E. cloni 10G SUPREME (Lucigen) or Transformax EC100D pir-116 (Lucigen) electrocompetent cells for the pGL4.10-Sasaki-SS (a) and pCpG-free- EF1α-SS (b) vectors, respectively, using the manufacturer's recommendations. To each electroporation cuvette, 1 ml of recovery media (Lucigen) was added, and the cells were incubated for 1 h at 37 C and 250 rpm. All cultures were then pooled and added to 5L of LB media with 100 ug/ml ampicillin (pGL4.10-Sasaki-SS; a) or 25 ug/ml zeocin (pCpG-free- EF1α-SS; b) and grown overnight at 37 C and 250 rpm until the O.D. reached 1.0. The bacterial cells were harvested by centrifugation and plasmid DNA was isolated using EndoFree Plasmid Giga kit (Qiagen).

Genomic DNA input library

Genomic DNA was isolated from GP5d colon cancer cells using DNeasy Blood and Tissue kit (Qiagen) and treated with RNAseA (Thermo Fisher) followed by purification. The genomic DNA was fragmented to an average size of ~500 bp using Covaris S220 according to the manufacturer’s recommendations. The fragmented genomic DNA (400 ng per reaction in ten reactions) was end-repaired, dA-tailed, and ligated to custom CpG-free annealed adapters (Oligo 3 - Custom CpG-free P7 adapter and Oligo 4 - Custom CpG-free P5 adapter; annealed according to the standard Illumina protocol). All adapter-ligated gDNA was purified and amplified for 10 cycles in 20 PCR reactions using KAPA HiFi master mix (Roche) and Oligos 5 and 6 which add the homology flanks for the NEB HiFi DNA assembly. The PCR product was pooled and purified using 0.8x volume of AMPure XP beads (Beckman Coulter) using the manufacturer's instructions followed by MinElute column purification. The pCpG-free-Sasaki-SS-v2 vector (d) was linearized using AflII and PvuII for 3h at 37 C and gel-purified. The PCR fragments were recombined to the vector in 50 parallel NEBuilder HiFi DNA assembly reactions (NEB) according to the manufacturer's instructions using 150 ng of linearized vector and 50 ng of custom CpG-free adapter-ligated genomic DNA. The reaction products were pooled (five reactions per column) and purified using MinElute columns (Qiagen) and eluted in water. The bacterial transformation was done in 50 reactions using 2.5 ul of purified eluate and 20 ul of Transformax EC100D pir-116 (Lucigen) by electroporation (Gene Pulser Xcell, Biorad) using the manufacturer's recommendations. To each electroporation cuvette, 1 ml of

recovery media (Lucigen) was added, and the cells were incubated for 1 h at 37 C and 250 rpm. The 50 cultures were then pooled and added to 6L of LB media with 25 ug/ml zeocin and grown overnight at 37 C and 250 rpm until O.D. reached 1.0. The bacterial cells were harvested by centrifugation and plasmid DNA was isolated using EndoFree Plasmid Giga kit (Qiagen).

Random enhancer oligonucleotide DNA input library

The random enhancer library was constructed from a 200-nt single-stranded Ultramer oligonucleotide (Oligo 7) harboring a 170-nt random sequence (170N) flanked by 15-nt constant sequences for library cloning. Double-stranded library was produced from 334 ng (5 pmol containing ~3×10^{12} molecules) of template oligo by employing a similar PCR strategy as described above for the TF motif library using Phusion DNA polymerase (Thermo Fisher) and Oligos 8 and 9 that introduce custom CpG-free sequencing adapters and flanking sequences homologous to the pCpG-free-Sasaki-SS-v2 vector (d). The NEB HiFi assembly between the random enhancer PCR product and the AflII-PvuII fragment from the pCpG-free-Sasaki-SS-v2, as well as electro-transformation and plasmid DNA isolation were performed as described above for the genomic DNA library.

Random promoter and random enhancer oligonucleotide DNA input library

The random promoter-random enhancer library was constructed by using two 190-nt single-stranded Ultramer oligonucleotides (Oligos 10 and 11) with 150-nt random sequences (150N). Each oligo harbors two 20-nt constant sequences that facilitate the library cloning to the pCpG-free-promoter-enhancer-SS vector (e). First, the constant sequences at the 3' ends of the oligos anneal to the pCpG-free-promoter-enhancer-SS vector in a PCR reaction with Phusion DNA Polymerase (Thermo Fisher), amplifying the region between AgeI and SalI sites from the backbone. Then, the PCR product constituting the random promoter-random enhancer library (of size 555 bp; including 150-nt random sequences, the synthetic intron, and the small Dm tal-1A ORF) was cloned into the pCpG-free-promoter-enhancer-SS vector (e) linearized using AgeI-SalI using the constant sequences introduced by the 5'ends of the oligos. The NEB HiFi assembly, electro-transformation, and plasmid DNA isolation were performed as described above for the genomic DNA library.

STARR-seq reporter library and input DNA library construction

First strand cDNA synthesis was done with 2.5-5 ug of polyA(+) RNA and with Superscript III (Invitrogen, #18080-044) using a reporter-RNA specific primer (Oligo 15) in 10-20 reactions depending on polyA(+) RNA yield. This was followed by RNase A treatment for 1 h at 37˚C and purification using MinElute PCR purification columns (Qiagen). cDNA amplification was performed with reporter-specific nested cDNA primers (Oligos 16 and 17 for libraries in vectors d and e, Oligos 16 and 18 for libraries in vector b, and Oligos 19 and 18 for libraries in vector a) using KAPA HiFi PCR Master mix (Roche) in the same number of reactions as done for the reverse transcription (98 C for 2 min, followed by 15 cycles of 98 C for 15 s, 65 C for 30 s and 72 C for 30-70 s). The PCR products were purified using 0.9X AMPure XP beads as per manufacturer's instruction followed by elution in nuclease-free water. The final PCR reactions to produce Illumina-compatible sequencing libraries were prepared from the entire amplified cDNA using KAPA HiFi PCR Master mix (Roche) at 98 C for 2 min, followed by 8-10 cycles of 98 C for 15 s, 65 C for 30 s and 72 C for 30 s. The primers used for different libraries are as follows: TF motif libraries in pGL4.10-Sasaki-SS (a) and pCpG-free-EF1α-SS (b) vectors were amplified using standard Illumina Universal and index primers (NEB #E7335S) and sequenced using standard Illumina chemistry. Genomic DNA and random enhancer libraries in pCpG-free-Sasaki-SS-v2 vector (d) were amplified using custom CpG-free primers (Oligos 20 and 21) and random promoter-random enhancer libraries in pCpG-free-

promoter-enhancer-SS vector (e) using Oligos 22 and 21. All custom CpG-free libraries were sequenced using custom read 1 and read 2 primers (Oligos 23 and 24) and custom i7 index read primer (Oligo 25). For preparing sequencing libraries from the input DNA, plasmid DNA from each library design was amplified in ten parallel reactions (10 ng DNA per reaction) using Phusion DNA Polymerase (Thermo Fisher) as above. For TF motif libraries in pGL4.10-Sasaki-SS (a) and pCpG-free- EF1α-SS (b) vectors, standard Illumina primers (NEB #E7335S) and sequencing chemistry were used, and for all the libraries in pCpG-free-Sasaki-SS-v2 (d) and pCpG-free-promoter-enhancer-SS (e) vectors, Oligos 20 and 21 were used for PCR amplification and Oligos 23-25 for sequencing. All libraries were sequenced either single-end or paired-end as per Illumina's standard instructions and protocols on suitable Illumina platforms like MiSeq, NextSeq500, HiSeq4000 and NovaSeq.

Template switch library preparation

Briefly, TurboDNase-treated RNA was incubated at 72 C for 3 min with a custom biotinylated STARR-seq specific RT-primer (Oligo 26) and dNTPs in two reactions having 25 ng of RNA per reaction, followed by immediate chill on ice. The first strand synthesis was performed using 100 units of Superscript IV RT (Invitrogen) in 1x SS-IV RT buffer containing 10 units of RNase OUT (Invitrogen), 5 mM DTT, 6 mM $MgCl_2$ (Sigma), 1M Betaine (Sigma) and 1 uM custom template switch oligo (Oligo 27) compatible with the custom CpG-free Illumina sequencing by incubating the reactions at 50 C for 15 min followed by 80 C for 10 min. cDNA was purified using AMPure XP beads (Beckman Coulter) and one cDNA reaction product was split into two for PCR amplification using KAPA HiFi master mix (Roche) together with USER enzyme (NEB) and Oligos 28 and 17 for enrichment of STARR-seq reporter-specific template (98 C for 3 min, followed by 15 cycles of 98 C for 20 s, 67 C for 15 s, 72 C for 6 min and final extension at 72 C for 5 min). The PCR product was purified using AMPure XP beads (Beckman Coulter) followed by a second PCR for a total of 5 cycles for Illumina library preparation using custom CpG-free Oligos 20 and 21. The final library was purified and sequenced using Oligos 23-25 on NextSeq and NovaSeq platforms.

Chromatin immunoprecipitation (ChIP-seq) and gene expression analysis (RNA-seq)

For ChIP-seq, fresh formaldehyde-crosslinked chromatin from the cells was used to immunoprecipitate DNA using Dynal-bead coupled antibodies, followed by standard ChIP-seq library preparation for Illumina sequencing. The libraries were single-read sequenced on HiSeq4000 and NovaSeq6000. The sequencing reads were aligned to the human genome (hg19) using bowtie2[17] (version 2.2.4) and peak calling (narrow peaks for TF ChIP-seq and activating histone marks and broad peaks for repressive histone modifications) was performed using MACS2[11] (version 2.1.1) using default parameters. Super-enhancers for GP5d cells were calculated using SMC1 and H3K27ac ChIP-seq data using the ROSE pipeline[18] (version 0.1). The peak files were filtered for the ENCODE blacklisted region (accession ENCSR636HFF) before further downstream analysis.

For gene expression profiling, total RNA was isolated using RNeasy Mini kit (Qiagen) with on-column DNase I treatment from the following conditions in three biological replicates: HepG2 cells with mock treatment (DMSO), with 350 μM 5-FU treatment, and after transfection of the genomic STARR-seq library using similar conditions as in the STARR-seq experiments described above at 24 and 48 h time points; GP5d cells 24 h after genomic STARR-seq library transfection. RNA-sequencing libraries were generated from 1 μg of total RNA samples using KAPA stranded mRNA-seq kit for Illumina (Roche) as per manufacturer's instruction and single-read sequenced on NovaSeq 6000 (Illumina). These data sets were used for analyzing the effect of plasmid transfection on cellular alarms by comparing gene expression between different treatments in HepG2 cells at 24 h time point (since the same time

point was used in the STARR-seq experiments), and for mapping the differential expression of TFs between HepG2 and GP5d cells. The transcript level counts were estimated with kallisto[19] version 0.46.1 in the strand-specific mode (option --rf-stranded) using Ensembl transcript annotation (Ensembl GRCh37 release-101 cDNA file). Differential expression analysis was performed with sleuth[20] version 0.30.0. Each treatment at different time points was treated as a separate condition, and the conditions were put into two models, one for the HepG2 treatments and another for the cell line comparison. Then differential expression was estimated by comparing individual conditions to a control condition (mock control when testing different HepG2 conditions and HepG2 expression when comparing two cell types) using the wald test. For gene set enrichment analysis (GSEA), gene lists for p53 and interferon signaling pathways were obtained from the Molecular Signatures Database (MSigDG v7.4, release c2.cp.v7.4.symbols.gmt) and preranked analysis using GSEA (version 4.1.0) was ran on the list of differentially expressed genes (preranked according to $\log_2$ fold-change from STARR-seq transfection vs. mock and 5-FU vs. mock) against these signatures. For comparing TF expression levels to motif activities in STARR-seq TF motif library in GP5d cells, the transcript abundances (transcripts per million; tpm) for three replicate samples were taken from RNA-seq data from ref. [21]. The expression of each TF in GP5d cells was summarized by taking the mean expression over the replicates for its most highly expressed transcript.

Chromatin accessibility (ATAC-seq)

The ATAC-seq libraries were prepared from 50,000 cells as previously described[22] for GP5d cell line and for HepG2 cells collected 24 h after the following treatments: mock (DMSO), 350 μM 5-FU treatment, and transfection of the genomic STARR-seq library using similar conditions as in the STARR-seq experiments described above. The cells were washed in ice-cold PBS and resuspended in 50 ul of lysis buffer and incubated for 10 min on ice. The pellet was lysed and transposed with Tn5 transposase in 2X tagmentation buffer (Illumina kit) and incubated for 30 min at 37 C. The reaction was purified using a MinElute purification kit and eluted in nuclease-free water. The samples were amplified for 5-8 cycles as determined by qPCR for Illumina sequencing using Nextera library preparation kit (Illumina) and samples were paired-end sequenced on HiSeq4000. The samples were demultiplexed and paired-end fastq files were processed using an in-house pipeline comprising of TrimGalore (https://www.bioinformatics.babraham.ac.uk/projects/trim_galore/), BWA aligner[23], Picard (http://broadinstitute.github.io/picard/) and broad-peak calling by MACS2[11]. The peak files were filtered for the ENCODE blacklisted regions as described earlier. In the genome browser snapshots, the traces from BAM coverage files are shown.

Active transcription factor identification (ATI) assay

The assay was performed *in vitro* by mixing 5 μl nuclear protein extracted from GP5d cells (2ug/ul), 5 μl 140 bp double stranded DNA (dsDNA) oligos[3] containing 40 bp random sequence in the middle (10 pmol), and 5 μl 3 × protein binding buffer (420 mM KCl, 15 mM NaCl, 3 mM K_2HPO_4, 6 mM $MgSO_4$, 300 μM EGTA and 9 μM $ZnSO_4$, 60 mM HEPES, pH = 7.5) and incubating for 30 min at room temperature. The poly-dIdC was supplemented in the reaction (5 ng/ μl final concentration) to decrease non-specific binding. Electrophoretic mobility shift assay (EMSA) was then conducted using commercial DNA Retardation Gel (Invitrogen, #EC63652BOX) in 0.5 × TBE buffer (1 mM EDTA in 45 mM Tris-borate, pH 8.0) at 106 V voltage for 70 min. The gel above the 300 bp DNA marker was collected, eluted in 300 μl Tris buffer (10 mM Tris-Cl, pH 8.0) and incubated at 65 °C for 3 h. The eluted DNA was amplified with Phusion polymerases (Thermo Scientific, #F530L); 4 pmol of each primer were used for the amplification. Before the final step of amplification, the same amount of primers was added to convert the remaining single stranded DNA (ssDNA) to dsDNA. The

amplified DNA library was incubated again with an aliquot of the same protein extract as above and the whole process was repeated for three more times. The PCR products from different cycles of ATI were purified and sequenced by Illumina Hiseq4000. The preprocessing of the data was done as described earlier in ref. [3]. The matching of known motifs was performed in the same way as for all other experiments (described below in **Matching of known motifs**).

Transient transcriptome sequencing (TT-seq)

Transcribed enhancer regions defined using TT-seq data are based on ref. [24]. Briefly, TT-seq from GP5d colon cancer cells was performed in two biological replicates as described[25]. TT-seq libraries were sequenced to a depth of ~120 million uniquely mapped paired-end reads and data analysis for identification of genomic intervals corresponding to continuous uninterrupted transcription (defined as transcription unit, TU) was performed using GenoSTAN[26]. TUs were classified into two groups: those overlapping with annotated protein-coding genes were defined as mRNAs and remaining as non-coding RNAs (ncRNAs). Putative enhancer RNAs (eRNAs) were further subclassified using histone modification patterns by defining a large set of putative enhancer regions from the publicly available datasets covering the non-coding regulatory genome[27,28]. eRNAs were further defined from the pool of ncRNAs on the basis of three criteria; first, origin should overlap within TSS ±500 bp (enhancer region), second, it should be outside of TSS ± 1 kbp (promoter region), and third, transcription is bidirectional as measured with TT-seq. This annotation led to identification of 6774 enhancer regions in GP5d colon cancer cells. The annotated enhancers were filtered for blacklisted regions as described before and also for chromosomes other than 1-22 and X.

Motif collection

For testing activities of known TF motifs, a set of 3226 HT-SELEX motifs were collected (refs [10,29,30] and unpublished draft motifs). A more compact set of motifs representing different binding specificities was generated by first constructing a dominating set (880 PWMs) covering motifs from the above sources using the same method and motif distance threshold as in ref. [29]. Then, in order to retain information of TF binding differences between methylated and non-methylated DNA ligand[10], for each methyl (or non-methyl) motif in the dominating set, the closest non-methyl (methyl) motif of the same TF was added to the representative set (respectively) if it was not yet in the set. This resulted in a representative set of 1121 HT-SELEX motifs (**Supplementary Table 3)**. In cases where HT-SELEX motifs for several TFs are highly similar, the motifs have been named in figures according to TF class or subclass, in order to highlight that we do not know which of the TFs that have similar motifs binds to the motif in the cells. Same principle has been applied also when specificities of closely related TFs have not been measured and thus can reasonably be expected to be similar. **Supplementary Table 5** shows the naming for the motifs in each figure. A control set of reversed but not complemented motifs was generated by reversing the column order of each motif matrix. Additionally, the following promoter core motifs were collected for TSS analyses from literature: TATA box, Initiator, CCAAT-box, GC-box from ref. [31], BRE, MTE, DPE from ref. [32], and BANP from ref. [33].

Motif library design

The synthetic oligo library design contained the 1121 HT-SELEX motifs in various sequence patterns. A pattern is defined as the combination and number of the motif consensus sequences, their relative orientation and spacings, and positions of degenerate N bases. Each of 727 monomeric and homodimeric motifs was included in the following patterns: consensus and its reverse complement (if palindromic added twice), reversed but not complemented control, each position of consensus at a time replaced with a degenerate base N, and two and

three copies of the consensus sequence in different orientations and spacings (three copies only for the motifs shorter than fifteen bases). Putting a degenerate base N at every position (in total 10,041 positions) generated in total 30,123 mutant consensus sequences.

In the patterns containing two and three copies of the consensus, the most defined position of the motif (position with maximum probability for any base in any position) was replaced with N. The two copy patterns included three relative orientations of the consensus C and its reverse complement R (CC, CR, and RC) and three copy patterns included four relative orientations (CCC, CCR, CRC, RCC). In the case of two copies, each orientation was included with all gap lengths from zero to six bases between the two copies. In the case of three copies the gap length was varied from zero to four bases (oligo length permitting) but the same gap length was used for both gaps in one pattern. Also, the consensus and reverse complement of the 394 heterodimeric motifs[34] was included. A subset of 245 heterodimeric motifs were also cut into two half-sites and the half-sites were added in the same patterns as two copies of a monomeric motif.

Each of the 43,251 motif patterns was embedded in two different sequence contexts. The contexts were chosen from two human genomic loci (context 1, chr10:77103489-77103535 and context 2, chr8:21525556-21525602, in hg19 coordinates) that do not contain high affinity sites of known motifs. Finally, 2-6 bases long random sequence (UMI) was put to the 5' end of the sequence to create an approximately uniform base distribution for sequencing. Both context sequences were also included alone with five different UMI lengths. Thus, in total 86,512 sequences were used for testing TF activity. The sequences and their embedded motif patterns are given in **Supplementary Table 4**. The remaining 6,406 oligos from the total 92,918 sequence patterns were composed of 3,576 SELEX nucleosome bound and unbound sequences, 1,182 draft HT-SELEX models for RNA binding proteins[35] and 1,648 for tiling genomic regions corresponding to enhancer regions for MYC and CCND2 (**Supplementary Table 4)**.

Enhancer activity of TF motif consensus sequences

For each TF consensus pattern, the reads containing the pattern were counted separately for the two sequence context and counts less than five in RNA and input DNA together were discarded from further analysis. The fold-change between RNA and input DNA was estimated using the function PsiLFC in R package lfc version 0.2.1[36] for each pattern in each context. To summarize the activity of one, two, or three copies of the motif, the median fold-change of all the patterns in both contexts containing the given number of the consensus sequences was used. For an individual consensus sequence, this included the patterns containing it or its reverse complement in both contexts. In the case of two or three copies, all consensus spacings and orientations were summarized together. The average fold-changes over all motifs in one, two, three copies were only calculated from those that could be detected with all copy numbers. If several sites acted without synergy, we assumed $\log_2$ fold-changes to grow linearly as a function of the number of sites. The motifs representing heterodimers were excluded from the analysis.

Analysis allowing base substitutions and generation of activity position weight matrix

For each pattern in a sequence context, the number of matching reads was counted both for the consensus sequence and its variations containing the designed base substitutions. The patterns detected at least 100 times in each context in RNA and input DNA together were considered. The $\log_2$ fold-change of a motif pattern in a context was estimated from the RNA and input DNA counts using the function PsiLFC in R package lfc. The activity PWMs were generated using the counts of the consensus sequence and all its single base substitutions. For each position of the consensus, the number of times each base was observed at that position in

the sequences otherwise matching the consensus was counted both in RNA and input DNA. Then such matrices were found that selecting from the input DNA sequences with them would generate the observed nucleotide distributions in STARR-seq RNA. In more detail, for each column independently a multinomial distribution was sought such that

$$s a_A b_A = o_A$$
$$s a_C b_C = o_C$$
$$s a_G b_G = o_G$$
$$s a_T b_T = o_T$$
$$a_A + a_C + a_G + a_T = 1$$

where

- a_N is the probability of the nucleotide N in the multinomial distribution
- b_N is the count of the nucleotide N in the input DNA
- o_N is the observed count of the nucleotide N in STAR-seq RNA
- s is a size factor to account for the different sequencing depths

Motif library complexity

Based on the sequencing of the pCpG-free-EF1α-SS input library, the motif library was estimated to contain approximately 26.9×10^6 distinct sequences when taking into account different UMIs (corresponding in total 1.3×10^9 bp), read counts are given in **Supplementary Table 7.**

Genomic library complexity analysis

Genomic STARR-seq input DNA library complexity was estimated using the preseq program[37] (version 2.0.0) lc_extrap tool that estimates how many distinct fragments would be observed based on reads in an initial sample if a given number of reads was sequenced. The mapped paired-end reads (bam) were given as input and the expected yield of 2.09×10^9 distinct fragments (assuming 2×10^{10} reads were sequenced) was used as the complexity estimate (10^{12} bp). Thus, the library was estimated to cover the human genome (3.2×10^9 bp) with 1.53 bp resolution on average. The resolution of STARR-seq output fragments in highly active genomic regions was broadly consistent with the high input resolution, taking into account that the fragment activity depends on which part of the regulatory element it covers.

Random library complexity and information analysis

The complexity of the random enhancer STARR-seq input DNA library was estimated by first creating a robust set of sequences originating from the same clones and their counts. This was done by clustering a randomly sampled set of input sequences using starcode[38] (version 1.3) so that first reads with edit distance four or less were connected and then reads in the same connected component were put to one cluster. The sizes of the sequence clusters were then given as input to preseq resulting in estimated 2.4×10^9 distinct sequences (corresponding in total 4.1×10^{11} bp). The same approach gave an estimate of 0.9×10^9 promoter sequences (1.4×10^{11} bp) and 1.2×10^9 enhancer sequences (1.8×10^{11} bp) in the binary STARR-seq input library. To confirm that these extremely complex libraries are effectively transfected to the cells, we estimated the number of plasmid copies per cell based on the comparison of read coverage between plasmid and genomic DNA from a control ChIP-seq experiment using a non-specific IgG antibody. This analysis revealed over 2500 plasmid copies per cell and based on this we estimate that each distinct random enhancer sequence was transfected to cells over 500 times on average.

To compare to the genomic conservation, we assumed that a TF motif typically has ~15 bits of information content. As it can be placed in ~320 positions in a 170 bp long random enhancer, one TF binding site contributes approximately $15 - \log_2(320) = 6.7$ bits of

information (see ref. [39]). Thus, a site would correspond to approximately seven conserved bases (> 1 bit of information).

Genomic STARR-seq analysis

First, demultiplexed Illumina STARR-seq RNA and input DNA paired-end reads (trimmed to common length 2 × 37 bp) were aligned to the human genome (hg19) using bowtie2[17] (version 2.2.4, option --maxins 1000). Before peak calling the mapped read pairs were deduplicated using picard (version 2.9.0) and paired-end reads that had mapping quality <20 or mapped in a discordant orientation were discarded using samtools version 0.1.19. MACS2[11] version 2.1.1 was used to call peaks in paired-end mode (-f BAMPE) so that the fragment endpoints were inferred from alignment results. Input DNA was used as control in peak calling and the called peaks were filtered for the ENCODE blacklisted regions (accession ENCSR636HFF) before subsequent downstream analysis. For masking repeats, a RepeatMasker file from UCSC table browser (for hg19) was used.

All STARR-seq RNA fragments from each cell line were used in peak calling (**Supplementary Table 7**) unless otherwise stated. Peaks were also called separately from two replicates in HepG2 cell line with 3295 peaks overlapping out of 6414 and 7376 peaks. IDR software[40] (version 2.0.3) was then used together with combined sample peaks to call high confidence peaks (2186 peaks with IDR < 0.1). In GP5d cells, genomic STARR-seq analysis was performed in two sublines (p53 wt and null) under two conditions (methylated or not), but due to the very large size of the experiments, replicates were not included for each condition. To enable calling reproducible peaks from the GP5d data, we utilized an internal control approach wherein two sets of peaks are built from a single replicate by splitting the fragments to two sets based on their mapping to either even or odd positions of the genome. These "*in silico*" replicates were then used for IDR analysis resulting in 1970 and 3250 high confidence *in silico* peaks (IDR < 0.1) in HepG2 and GP5d wt cells, respectively. Comparison of the high confidence peaks from biological and *in silico* replicates in HepG2 cells revealed that this IDR method yields similar peak-calls (~90% specificity if biological replicate analysis is considered ground truth; see **Extended Data Fig. 5a**). Together, these analyses show the high reproducibility of the strong genomic STARR-seq peaks.

Genomic feature overlap analysis

Overlaps between the GP5d genomic STARR-seq and other genomic features were calculated from peaks called with MACS2[11], with the exception of the TT-seq enhancers for which the estimated enhancer regions were used. Only chromosomes 1-22 and X were used in the analysis. All overlaps between the peaks were calculated using Bedtools[41] (version 2.25.0). The motifs used in calculating the matches to STARR-seq peaks are listed in **Supplementary Table 5**. For comparison of STARR-seq peak overlap with ATAC-seq, H3K27ac ChIP-seq, and TT-seq enhancers (**Extended Data Fig. 5c,** left), Fisher's exact test two-tailed P values were calculated using "bedtools fisher"[41] after filtering out the positions in the ENCODE blacklist (peaks hitting these regions would not be considered). Note that in most cases the overlaps are much larger than expected purely at random leading to P value s that are too small to represent as floating-point numbers. These cases are marked as $P<2.2251\text{x}10^{-308}$.

Genomic STARR-seq in HepG2 cells was compared with data downloaded from the ENCODE project: ATAC-seq (ENCSR042AWH, replicate 1), histone modification ChIP-seq experiments for H3K27ac (ENCSR000AMO), H3K27me3 (ENCSR000AOL), and H3K9me3 (ENCSR000ATD), and H3K4me1 (ENCFF424GUI), as well as ChIP-seq data sets for TP53 (ENCSR980EGJ), MED1 (ENCFF493UFO), and MED13 (ENCFF003HBS). The IDR-thresholded peaks and bigWig signal files showing fold-change over control generated from all replicates were used except for ATAC-seq and TP53 ChIP-seq. The reads for ATAC-seq

replicate 1 were reanalyzed in hg19 coordinates in the same way as GP5d ATAC-seq data resulting in 60,500 peaks. For TP53 ChIP-seq, the ENCODE peaks were lifted over from GRCh38 to hg19 coordinates and the reads for replicate 1 were remapped to hg19 and MACS2 was used to generate a normalized coverage file. For the rest of the TFs and other chromatin-associated proteins, the ChIP-seq peaks were taken from ref. [42] (GEO accession GSE104247), and the corresponding bigWig files were downloaded from the ENCODE portal. Super-enhancers for HepG2 are from http://www.licpathway.net/sedb. The overlaps between different features were calculated using bedtools (chromosomes 1-22, X and Y). The Euler (**Fig. 4a**; R package eulerr) and bar (**Extended Data Fig. 6a;** R package UpSetR) diagrams show other features overlapping the top quartile of all ATAC-seq and STARR-seq peaks according to maximum fragment coverage. When calculating overlaps between chromatin-associated proteins in open regions with or without STARR-seq signal (**Extended Data Fig. 6a**), only STARR-seq peaks with IDR < 0.1 calculated from two STARR-seq replicates were used. The PolII-associated proteins POLR2AphosphoS2, PAF1, POLR2A, ZC3H4, TBP, POLR2AphosphoS5, and SSRP1 were excluded from this overlap analysis resulting in 202 proteins. In the overlap analysis, all Ensembl TSS (GRCh37, release 101) extended 1 kb to both directions were used.

***De novo* motif mining**

De novo motif mining for the peaks called from ATAC-seq, ChIP-seq and genomic STARR-seq were performed using HOMER[43] (version 4.10.3). Sequences used for motif mining of different enhancer classes in HepG2 cells were based on the intersections as in Euler/UpSet diagrams in **Fig. 4a; Extended Data Fig. 6a**, with the overlaps calculated against top quartile of the ATAC or STARR-seq peaks, respectively. In addition, the sequences overlapping TSS-regions (from Ensembl GRCh37, release 101, extended 1 kb to both directions) were excluded, resulting in 1,524 closed chromatin enhancers, 971 classical enhancers, and 3,797 chromatin-dependent enhancers that were used in the analysis. For the sequences enriched from the random enhancer and random promoter-random enhancer STARR-seq experiments and from the ATI assay, *de novo* motif mining was done using the "Autoseed" program[30] as described earlier[3,30]. From the random enhancer STARR-seq in GP5d cells, the sequences were cut to 40 bp long non-overlapping subsequences starting from position 6 and ending at position 165, and the subsequences containing N were removed (see **Supplementary Table 7** for the numbers of analyzed subsequences). "Autoseed" program[30] was also used to mine *de novo* motifs enriched at specific positions in relation to TSS using TSS-aligned sequences from GP5d cells (see section "Mapping TSS positions based on template switching") and input DNA sequences sampled from the same positions (**Supplementary Table 7**). The resulting seven *de novo* motifs were mined from the subsequences spanning the following positions in relation to TSS: two initiator motif variants from position one at TSS (only forward strand), CREB motif and two CREBMAF heterodimer variants from -30 to 30, and TATA box promoter from -30 to 23. These motifs were also included to motif enrichment comparison between promoter and enhancer sequences.

In addition to the "Autoseed" analysis, we performed *de novo* motif discovery using STREME[44] program (version 5.3.3) from the common 371,390 enhancer sequences that were observed in both replicates from random enhancer STARR-seq in GP5d cells compared to randomly sampled input sequences. Due to the large size of the data set, only one seed per each motif discovery round was optimized using the iterative refinement algorithm and half of the whole data was used as the hold-out set to accurately estimate P values also for rare but highly enriched motifs (options --hofract 0.5 --nref 0 --minw 6 --maxw 20). The discovered significant motifs (P value < 0.05) were compared to known TF motifs using TOMTOM[45] web-service (version 5.3.3) with default options.

Conservation of genomic STARR-seq elements

Conservation of genomic STARR-seq peaks and input fragments was analyzed by calculating their average GERP scores[46] using precomputed base-wise GERP scores (hg19) from ref. [47] (http://mendel.stanford.edu/SidowLab/downloads/gerp/). In the analysis, only chromosomes 1-22 were included, and the elements overlapping ENCODE blacklisted regions (ENCFF419RSJ) and UCSC RepeatMasker[48] "Repeats" track were discarded. A base pair was deemed conserved if its GERP score was higher than the average GERP score (~2.2) of the coding sequence[47]. Additionally, the GERP scores were calculated for three known enhancers as detailed below.

- GP5d genomic STARR-seq peaks, mean GERP score = 0.14. The average number of conserved base pairs in 170 bp surrounding the STARR-seq peak summits was ~50.8 (~169.4 for whole peaks, corresponding to ~42.6 when the average width of a STARR-seq peak, ~675.5 bp, is scaled to 170 bp).
- 100,000 randomly sampled genomic STARR-seq input fragments, mean GERP score = 0.01.
- The MYC335 enhancer (chr8: 128413174-128414429)[49], mean GERP score = 3.07, number of conserved base pairs = 921.
- The SHH enhancer (chr7: 156583796-156584568)[50], mean GERP score = 3.66, number of conserved base pairs = 642.
- The Sox9 enhancer (chr17: 69480826-69481362)[51] with respective coordinates for human SOX9 enhancer (hg19) obtained using UCSC genome LiftOver tool (https://genome.ucsc.edu/cgi-bin/hgLiftOver), mean GERP score = 0.70, number of conserved base pairs = 230.

Matching of known motifs

The motifs were matched to sequences using MOODS[52] (version 1.9.3). The matching was done separately for each strand using strand- and sample-specific nucleotide frequencies so that the background probability of a match was 10^{-6} unless otherwise stated. When calculating motif matches for logistic regression PWM features, affinity threshold of 2 was used instead of a background probability threshold. Motif matches that resulted in occupancy probabilities smaller than 0.01 were discarded (see Logistic regression classification for details).

To determine the activity of an individual motif, the total number of its matches in the sequences was counted so that overlapping matches in different strands were counted only once. Then fold-changes between the motif match counts in RNA and a randomly sampled subset of input DNA were estimated using the function PsiLFC in R package lfc. This approach together with sample-specific motif matching effectively eliminated the effect of differing mononucleotide frequencies between samples. For random enhancers in GP5d cells the Pearson correlation between the percentage and the number of G and C in motif consensus and its enrichment for the most active 10% of motifs were -0.15 and -0.05, respectively, and for all motifs 0.17 and 0.15, respectively. When comparing two different RNA samples (for example two replicates), two different random samples of input DNA were used to avoid overestimating similarities by using identical input counts. To estimate the effect of the number of binding sites in the same sequence, the number of sequences having exactly two non-overlapping motif matches was counted, and the fold-change was compared to the fold-change of those having exactly one match. If the site occurrences are independent of each other, the expected frequency of several sites is the product of the individual frequencies. Thus, the expected $\log_2$ fold-change assuming independent actions of several motif occurrences was calculated as the sum of their individual $\log_2$ fold-changes.

Analysis of motif spacing

For the motif spacing analysis in STARR-seq random enhancers, the most enriched 10% of the motifs in GP5d cell line were chosen (73 motifs as heterodimer motifs excluded, $\log_2$ fold-change of the chosen motifs was over 0.19 when matching done with P value cut-off 10^{-6}). For the chosen motifs, the matching was done with a relaxed P value cut-off 10^{-4} against pooled sequences from both GP5d replicates and from each enhancer sequence only the strongest match for each motif was taken (so pairs of the same were not considered). For each motif pair, all overlapping motif matches were removed and for the remaining motif match pairs the distances between motif match information content centers were calculated and pairs having distance over 30 bp were discarded (the example cases shown in **Fig. 2c** were re-analyzed also with the maximum distance 50 bp shown). For each of 5,256 pairs (including the same motif pair in both orders), each configuration (orientation of the motifs relative to each other and distance between the information content centers) was separately tested for enrichment using one-sided Fisher's exact test to see if there was an association between the two classifications i) the particular spacing and orientation vs. all others and ii) motif matches in STARR-seq RNA vs. input DNA. The individual P values were corrected for the number of configurations tested for the pair using Holm's method. For 46 pairs the false discovery rate of the best configuration was < 0.1 (Benjamini Hochberg method used to correct for the total number of tested motif pairs). The p53-family motif match spacing with another match of itself was analyzed in the same way except that several matches of the motif in a sequence were considered.

The statistical power to detect specific motif spacings and orientations was analyzed by generating the same number of simulated pairs as there were tested pairs and then for each simulated pair the same number of configurations (n) as there were on average per tested pair. The input counts of the different configurations were then generated from the multinomial distribution so that each configuration had a success probability $1/n$ and that the total count matched the average DNA input count of a tested pair given the motif match threshold. Then for each simulated true positive pair with the fold-change f, the STARR-seq RNA counts were generated from the multinomial distribution so that one configuration had a success probability f times the probability of the other configurations (null hypothesis false). For the rest of the simulated pairs (null hypothesis true), the RNA counts were as for the input using probability $1/n$ for all configurations (null hypothesis true). In both cases the total count was set to the average STARR-seq count of a tested pair. Statistical testing was done for the generated counts as for the tested cases. Finally, for each fold-change, the proportion of the simulated true positive pairs for which the null hypothesis was rejected with a false discovery rate <0.1 was reported as the power of the test.

Mutual information (MI) analysis

The binding events on the aligned STARR-seq reads (60+60 bp surrounding TSS from the template switch data) were analyzed by calculating the mutual information (MI) between 3-mer distributions at two non-overlapping positions of the aligned sequences. MI can be used to capture binding events, because if the binding contacts two continuous or spaced positions (3-bp wide) on the sequences at the same time, correlations will be observed for the 3-mer distributions at the two positions. Subsequently, the biased joint distribution will be detected as an increased MI between the positions. For two non-overlapping positions (pos1, pos2), the MI between them was estimated as described in ref. [53]. The calculation uses the observed frequencies of a 3-mer pair (3+3-mer), and of its constituent 3-mers at both positions:

$$MI\ (pos1, pos2) = \Sigma\ P(3\text{+}3\text{-mer})\log_2\frac{P(3+3-mer)}{Ppos1(3-mer)Ppos2(3-mer)}$$

where *P(3+3-mer)* is the observed probability of the 3-mer pair (i.e. gapped or ungapped 6 mer). P_{pos1}*(3-mer)* and P_{pos2}*(3-mer)*, respectively, are the marginal probabilities of the constitutive 3-mers at position 1 and 2. The sum is calculated over all possible 3-mer pairs using 17,235 TSS sequences. When calculating the positional 3-mer and the pairwise 3+3-mer distributions, a pseudocount of 10 was added to each k-mer. Pseudocount was used to alleviate the effects of estimating MI from a finite sample of sequences. Using pseudocount means assuming that when the observed sample count of a k-mer approaches zero, the estimated probability of observing this k-mer approaches a non-zero constant (instead of zero without a pseudocount). Reasonable changes in magnitude of pseudocount only affect the scaling of values of MI, not the position or shape of dependencies highlighted. Mean mutual information between 3-mer distributions separated by specific distances was computed by taking the mean of each diagonal of the mutual information matrix computed as described above.

Data preprocessing for machine-learning analysis

The datasets used in each machine-learning analysis and their division into training, test and validation sets are detailed in **Supplementary Table 7**. To enable sequences from genomic measurements (genomic STARR-seq and ATAC-seq) to be scored on the CNNs that were trained on the random enhancer STARR-seq data and vice versa, the length of the sequences fed to these models was standardized to 170 bp. Thus, additional preprocessing specific to machine-learning analyses was done for the genomic STARR-seq and ATAC-seq data.

First, an extended blacklist file was created to remove possibly problematic genomic regions that might cause the machine-learning models to learn biases instead of real signals. In addition to the standard ENCODE blacklist (ENCFF419RSJ), this extended blacklist contains all positions ±1Mb from centromeres, all positions with Ns in the hg19 reference genome and non-uniquely mapping regions that were defined as follows: All unique 55-mers present in the hg19 reference genome were fetched and aligned back to hg19 reference with bwa aln[23] algorithm (version 0.7.15-r1142-dirty). Then each position that was not covered by reads mapped with MAPQ>20, was added to the extended blacklist. This extended blacklist covers around 12% of the hg19 reference genome.

GP5d genomic enhancer fragments were created by fetching the sequence (hg19) that maps between the paired end reads. The signal set (class 1) sequences were created by taking the 170 bp closest to the peak summit from each genomic STARR-seq fragment that overlaps with a GP5d genomic enhancer STARR-seq peak. Balanced control sets of 170 bp sequences (class 0) were sampled from the genomic STARR-seq input requiring that the reads map and do not overlap with regions covered by the extended blacklist or with GP5d genomic enhancer STARR-seq peaks. To ensure that the classifier does not learn any features possibly correlating with different input library coverage between the class 1 and class 0 sequences, the class 0 sequences were sampled in such a way that their input library coverage histogram matched the input library coverage histogram of the class 1 sequences. Input library coverage of each class 1 and class 0 sequence was calculated by counting how many input library fragments each of them overlaps. Then 36 evenly sized bins were created so that the first bin included coverages between 0 and 9 and the last between 350 and 359 (354 was maximum coverage for class 1 sequences). Then class 0 sequences were sampled so that their count in each bin equaled the count of class 1 sequences in that bin for each set (training, test, validation) separately. Sequences from random STARR-seq experiments were not mapped to any reference sequence at any point, so this precaution is not relevant with random STARR-seq experiments.

The GP5d ATAC-seq single-end short reads were extended to the average fragment size of the library (300 bp) by adding 300-readlen to 3'-end of each read (where readlen is the length of each read). For the class 1 signal set, extended fragments that overlap with any ATAC-seq peak were selected and the 170 bp sequence closest to the overlapping peak summit

was retrieved from the fragment. Exact duplicate sequences were discarded. A balanced negative set (class 0) was created by sampling random 170 bp sequences from the genome and not allowing them to overlap with ATAC-seq peaks or regions covered by the extended blacklist.

Logistic regression classification

The logistic regression classifiers were implemented using the LogisticRegression function from scikit-learn (version 0.21.3) library[54]. All logistic regression models were regularized with L1 norm (LASSO). Using L1 norm as regularization is important for the interpretability of the model coefficients, as the set of PWMs used as features of the model (**Supplementary Table 10**) contains several matrices that can be very similar to each other. Thus, a non-regularized model, or a model regularized with L2 norm (ridge), could split effects into coefficients of similar PWMs. Using the L1 norm that penalizes solutions with a higher number of non-zero coefficients enables finding the best performing model with the lowest number of individual PWMs contributing to the model. Regularization with the L1 norm is known to perform well with correlated features given that the regularization strength is properly adjusted[55]. The optimal regularization strength was chosen based on the area under precision-recall curve on the validation data (see **Supplementary Tables 8, 9** for the tested and final values, respectively). Classification performance on unseen test data is shown for each trained hyperparameter combination in **Supplementary Fig. 4**. Otherwise the regression was run on default parameters. First, a logistic regression classifier was trained using only features that count matches of individual PWMs (880 features, **Supplementary Table 10**). After this, a more complex classifier was fit with additional features counting all self-pairs (A_i+A_i, i runs over all the 880 PWMs), and all pairs of the top 20 strongest individual features (20 features with largest absolute value of the regression coefficient) from the simpler model (**Supplementary Table 11**) with all other PWMs (S_j+A_i, i runs over all the 880 PWMs, j runs over the top 20 PWMs from the simple model of 880 features). Thus, this more complex model is expected to cover all meaningful general pairwise interactions between the binding motifs of HT-SELEX PWMs. We also trained simple logistic regression models using the set of 22 *de novo* binding motifs discovered with STREME[44] (see **Supplementary Fig. 2a**) as features.

For each feature (corresponding to a PWM, X), the probability that a given read is occupied by a given TF or TF-pair is calculated by using an approach derived from ref. [56], where:

$$P_{X,read} \approx 1 - \prod_{i=1}^{N_{sites}} (1 + [X]/K_{d,X,i})^{-1},$$

where N_{sites} is the number of motif matches for PWM X in the read, $[X]$ is the free concentration of the TF corresponding to the PWM X and $K_{d,X,i} = exp(-\Delta G_{X,i}/RT)$ is the equilibrium dissociation constant of the binding site i of the TF corresponding to the PWM X. In ref. [56], the free concentration of each TF was set to equal the K_d of the consensus sequence. However, for some TFs with a long PWM, exact matches to the consensus sequence are rare, and setting the scoring as described above will result in the occupancy scores 0 for many functional binding sites, reducing the variance of the scores of the variables corresponding to these TFs. To overcome this, we used a normalization approach based on the fact that TFs generally have ~10000-300000 binding sites in the human genome and defined the free concentration of each protein to correspond to the K_d of the strength of its 10000th strongest binding site in the human genome. Thus,

$$P_{X,read} = 1 - \prod_{i=1}^{N_{sites}} (1 + exp(S_{X,i} - S_{X,10000}))^{-1}$$

where PWM match score of the 10000th strongest match of the PWM X is $S_{X,10000}$ and the PWM match score of the ith site in a read is $S_{X,i}$, This normalization was calculated separately for each PWM and strand. The probability for a pair of TFs (X+Y) to occupy a sequence[56] is

$$P_{XY,read} \approx 1 - \prod_{i=1}^{N_{sites}} \left(\prod_{j=1}^{M_{sites}} (1 + exp(S_{X,i} + S_{Y,j} - S_{X,10000}))^{-1} \right).$$

Position-specific logistic regression classification

The positional activity scores of the 880 dominating set PWMs and the 7 core promoter PWMs (see **Supplementary Table 12**) were used to train a position-specific logistic regression classifier on the promoter capture STARR-seq data. In contrast to the simple logistic regression described above, the PWM match scores were weighted using the positional activity scores of the corresponding PWM. Thus, instead of occupancy probabilities, scores

$$A_{X,read} = \sum_{i} a_{X,i} \cdot S_{X,i}$$

were calculated for each sequence and PWM feature X, where $a_{X,i}$ is the positional activity score of PWM X at position i and i runs over all matches of PWM X in a sequence. Regression coefficients were learned separately for both strands for each PWM. Training of the position-specific regression model was done similarly to the training of other logistic regression models. Final model was selected based on area under precision-recall curve on separate validation data. Classification performance on unseen test data is shown for each trained hyperparameter combination in **Supplementary Fig. 4**.

Convolutional neural network classification

The CNN classifiers used the raw fasta sequences as input to learn the features during the training process. The random enhancer CNN and the genomic enhancer CNN were trained using also the reverse complement sequences of the training data, all other models were trained using one orientation only. The CNN models consist of convolutional modules with a 1D convolutional layer followed by batch normalization, ReLu activation and a dropout layer. The number of convolutional modules, the number of convolutional filters per layer, and the dropout rate are hyperparameters that were optimized based on the validation data. The convolutional modules used dilated convolution[57] so that the dilation rate of the ith layer is i^2, which allows learning interactions with fewer parameters than fully connected (dense) layers. After the convolutional modules, the final layer is a dense layer of two nodes with sigmoid activation. This CNN architecture was selected by testing it against architectures with varying sized dense layers after the convolutional layers. Adding the dense layers did not improve the performance of the models. The models were built on Keras (https://keras.io/; version 2.2.4) using TensorFlow 1.14.0 backend[58].

Models were trained using the Adam optimizer with default parameter values. Training was stopped once binary accuracy on validation data did not improve within 200 epochs or when the total training time on a single Nvidia Volta V100 GPU exceeded 72 hours (an exception being the "double input" CNN models trained to classify the "binary STARR-seq" data (see below for more details) where training was continued up until 144 hours if needed). Model parameters were initialized using the He uniform variance scaling initializer[59]. All models, except the double input CNN, were trained on batch size 128 as changing the batch

size had a negligible effect on classification performance, and only had one convolutional body and no dense layers (except the final output layer). Batch size for the double input CNN models was determined over grid search between 32 and 64. The other hyperparameters of the CNN models were determined by grid search over the values shown in **Supplementary Table 8**, and the hyperparameters of the final selected models are shown in **Supplementary Table 9**. Classification performance on unseen test data is shown for each trained hyperparameter combination for each trained CNN model in **Supplementary Fig. 4.**

Note on optimal classification of random enhancer STARR-seq data

As seen from **Supplementary Fig. 4**, classifying between random enhancer STARR-seq signal and input sequences is more difficult than for other datasets studied here. We believe that this limited classification accuracy is mainly a feature of the experimental design. Transcription of one molecule of DNA is an inherently probabilistic single-molecule process, and therefore some transcription occurs at random. Some sequences are thus recovered due to this "transcriptional noise", limiting the maximum classification accuracy. To determine whether a better classification is possible, the replicate experiment of the GP5d random enhancer STARR-seq was utilized to create an unseen test set that contains only those original test set sequences that were also observed in the replicate experiment (2.8% of sequences were observed in both replicates). A balanced number of input library sequences were used as class 0 sequences. Using the final GP5d random enhancer STARR-seq CNN model on this high-confidence test set resulted in ~4% increase in AUprc (**Extended Data Fig. 3b**). The mean(AUprc) ± std from eight CNN models with different hyperparameters was $AUprc_{all}$=0.614±0.004 for the test set with all sequences and $AUprc_{common}$=0.641±0.003 for the common sequences between the replicates.

We also further removed the sequences observed more than once in the input library from the 371,390 common STARR-seq sequences between the GP5d random enhancer STARR-seq replicates to evaluate whether possible uneven sequence representation in the input library could affect the classification. For each common STARR-seq library sequence between replicates, the number of matches in the input library was counted by requiring the first 60 bases of the common sequence to match exactly to the first 60 bases of a read 1 sequence in the input library. There were only 6,213 (~0.02%) common sequences between replicates that were observed more than once in the input library (total 999,854,466 input sequences). Removing these 6,213 sequences had a negligible effect on the classification (**Extended Data Fig. 3b**).

As an external evaluation we trained the previously published gapped k-mer SVM classifier[60,61] on the high-confidence GP5d random enhancer STARR-seq data where the signal set (class 1) sequences were observed in both of the replicates. A balanced set of class 0 sequences were sampled randomly from the input library. We used the default k-mer length (11) and the default decay strength for the center weighted gkm kernel, and tested both "gapped k-mer", and "gapped k-mer + center weighted" kernels. We optimized the regularization strength over values (1, 0.1, 0.01, 0.001) with the validation data (by calculating area under precision-recall curve). The best hyperparameter combination was obtained by using gapped k-mer kernel and regularization strength=0.1, producing AUprc=0.6207 on the validation data. With the unseen test set (high-confidence sequences) this model obtained AUprc=0.6086. With the full test set from GP5d random enhancer STARR-seq replicate 1 the gapped k-mer SVM obtained AUprc=0.57, which is slightly better than the pairwise logistic regression (0.56) but worse than the CNN (0.62). The gapped k-mer SVM training with the full GP5d random enhancer STARR-seq training set did not complete within approximately three weeks of running time on our computational cluster with 16 parallel threads (gapped k-mer SVM maximum), so performance of a model trained with the full data set could not be evaluated.

Differential expression prediction

A lasso regression model was used to study the extent to which differential gene expression between GP5d and HepG2 cell lines could be predicted using STARR-seq and ATAC-seq derived features. Logarithmic fold-change between GP5d and HepG2 expression values (tpm) was used as the target variable for regression (see section "Chromatin immunoprecipitation (ChIP-seq) and gene expression analysis (RNA-seq and CAGE)" above). The STARR-seq and ATAC-seq peaks were divided into 12 explanatory features based on the cell line in which the peaks were observed and the information about whether the ATAC-seq peaks were promoter proximal (less than 1kB distance to any gene in the target gene set) or not as follows: Common.ATAC.enh.noSTARR; Common.ATAC.enh.yesSTARR; Common.ATAC.prom; Common.STARR.noATAC; GP5d.ATAC.enh.noSTARR; GP5d.ATAC.enh.yesSTARR; GP5d.ATAC.prom; GP5d.STARR.noATAC; HepG2.ATAC.enh.noSTARR; HepG2.ATAC.enh.yesSTARR; HepG2.ATAC.prom; HepG2.STARR.noATAC. Naming "Common.STARR.noATAC" means STARR-seq peaks that were observed in both cell lines and that did not overlap with an ATAC-seq peak, and "HepG2.ATAC.enh.yesSTARR" means HepG2-specific ATAC-seq peaks that are not within 1 kb from target gene TSSs and do overlap with STARR-seq peaks.

For each feature, the logarithmic fold-change at peak summit reported by MACS2 (LFC) was used as the strength of the peak. Peak summit position was used as the position of the feature (ATAC-seq peak summits were used for all other features except the ones that had no overlap with ATAC-seq). Peak score S, meaning the effect of a peak to a TSS that is d bp away from the peak summit for each feature was calculated assuming it decays like:

$$S(peak) = LFC \cdot exp(-c \cdot d / d_{max}),$$

where LFC is the logarithmic fold-change at peak summit, c is a scaling parameter and d_{max} is the maximum distance of a peak from the TSS for its effect to be included in the model. This peak score S was used to quantify the effect of each feature in the regression model.

The target genes were split into training (8815 genes), validation (1449 genes) and test (2321 genes) sets according to chromosomes they are in (training: chr1, chr3, chr5, chr7, chr9, chr11, chr13, chr14, chr15, chr16, chr17, chr18, chr19, chr20, chr21, chr22, chrX; validation: chr4, chr6, chr8; test: chr2, chr10, chr11). A lasso regression model was trained using the LassoCV method in scikit-learn[54] library (version 0.24.1) where the regularization strength (L1 norm) was determined using 5-fold cross-validation during training (n_alphas=100, no intercept term). On top of this, the model hyperparameters c and d_{max} were optimized using the validation data set with a grid search over the values shown in **Supplementary Table 8**. Features were standardized by removing the mean and scaling to unit variance before model training. The best model had the following hyperparameters: d_{max}=100,000 bp; c=4.3; regularization strength α=0.013203848400344683. This means that the effect of a peak decays to ½ at approximately 16,120 bp from the peak summit. This model obtained R^2 = 0.1227036350306634. Regression coefficients of this best performing model are shown in **Supplementary Table 6.**

Interpretation of convolutional neural network classifiers

To test which HT-SELEX PWMs the convolutional neural network had learned, the trained CNN was used as an "oracle" as in ref. [62] by embedding 100 sequences drawn randomly from each PWM to random enhancer STARR-seq input library sequences (**Supplementary Table 10)**. Each such sequence was embedded at a random position to one of 100 different randomly chosen input sequences (same input sequences used as background for each PWM) and the average enhancer probability over the 100 sequences was calculated for each PWM. When embedding a single PWM per input sequence, first, the position for the embedding was

drawn from random uniform distribution. Next, the embedded sequence was drawn by random from the corresponding PWM. When embedding a motif pair, first the positions of the embedded sequences were drawn at random not allowing overlap between the embedded sequences. Then, both of the embedded sequences were drawn independently from the corresponding PWMs. Thus, both the positions and the distance between the embedded sequences are random. The expected enhancer probability for a sequence with two embedded PWMs given that there are no interactions between them is $p_2 = 1 - (1 - p_1)^2$, where p_i is the enhancer probability of a sequence with i PWMs embedded, representing the cumulative probability for geometric distribution with two trials.

As complementary approaches, we also used *de novo* motif mining as well as DeepLIFT[8] (version 0.6.12.0) and TF-MoDISco[9] (version 0.5.14.1) analyses to study the sequence features learned by the random enhancer STARR-seq CNN model. For *de novo* motif mining, we created a set of 10 million 170 bp long DNA sequences that were sampled from random uniform nucleotide distribution. These sequences were then scored with the CNN model, and the top 0.5% of the sequences (50,000) obtaining highest predicted enhancer probabilities were selected for *de novo* motif mining analysis with STREME[44] (version 5.3.3) using the same parameters as used for mining motifs from random STARR-seq sequences.

DeepLIFT[8] software shows the importance of each position in a sequence for the final prediction verdict. In our binary classification tasks, positive values correspond to sites that move the prediction verdict towards the active class of sequences and negative values to sites that move the verdict towards the inactive class. We used DeepLIFT to test if the CNN model has learned similar motifs than the simple logistic regression by constructing "CNN activity contribution weight matrices" (CACWM) with the "N-sweep" algorithm using a strategy similar to what was used for generation of activity position weight matrices from the motif library. First, the 20 most important PWM features were selected from the simple logistic regression model (**Supplementary Table 11**) and their consensus sequences obtained. Then, for each position in each of these PWMs, the four possible variants of the sequence were created, and each variant sequence was embedded separately into two different positions in ten randomly sampled background sequences from the random enhancer input library. For each of these 80 sequences per position and per PWM, DeepLIFT contributions were computed against ten dinucleotide-shuffled versions of the sequence itself. The resulting DeepLIFT contribution at position i of the variant was then added to position i of the CACWM for the nucleotide n that is at position i in the variant sequence. Finally, each position of the CACWM was normalized by dividing with the number of different sequences used per variant (20).

To complement the *de novo* motif discovery, we also ran TF-MoDISco[9] analysis on the same set of top 0.5% highest-scoring sequences according to the random enhancer STARR-seq CNN. First, DeepLIFT contributions were computed for each of the sequences using ten dinucleotide-shuffled references. DeepLIFT hypothetical importance scores were used with TF-Modisco, which was run on default parameters except sliding window size was set to 30, flank size was set to 5, and final minimum cluster size to 60. Motifs discovered by TF-MoDISco were named according to a scan against known motifs with TOMTOM[45] software (version 5.4.1) with default parameter values. The HNF4 motif in **Extended Data Fig. 4** is named based on manual curation even though that corresponding TF-MoDISco pattern did not have significant (E-value threshold 0.05) matching motifs according to the TOMTOM analysis.

The *TERT* promoter sequences[13,14,16] were visualized with the DeepLIFT software[8]. The activation values from the wild type and mutated promoter sequences were compared against 15 randomly selected background sequences from the random promoter STARR-seq input and their average signals were visualized. Predicted promoter probabilities of the *TERT* promoters were obtained by scoring the sequences with the CNN trained on promoter capture STARR-seq data.

To visualize pairwise position-specific interactions learned by the CNN classifiers trained on STARR-seq and EPD promoters, respectively, the high-confidence promoter sequences were visualized with the same mutual information (MI) analysis pipeline[53] as described above. Here, we generated 10 million sequences of length 120 bp from random uniform nucleotide background and scored each of them with the 10 best (according to binary accuracy on the validation data) STARR-seq promoter capture CNN models and 10 best EPD promoter models. Those sequences obtaining a promoter probability of 0.9 or higher according to each of the 10 models were selected for MI analysis. This resulted in 51,131 sequences for STARR-seq promoter capture CNN and 395,663 sequences for EPD promoter CNN. These numbers together with the MI-plot themselves in **Extended Data Fig. 8f,g** indicate that the STARR-seq data allowed learning models with stricter positional dependencies.

Validation of the predicted promoter mutation effects with external data

To validate the effect of mutations predicted by the CNN model trained on promoter capture STARR-seq data, the model predictions were correlated with a saturation mutagenesis study of the same promoter[15] (see **Extended Data Fig. 8b-d**). The statistical significance of the mutation effects predicted by the CNN model was estimated as follows. First, the predicted promoter probabilities from the CNN model were transformed into log odds scores logit(p)=log(p/(1-p)), and the predicted effect of each mutation was calculated as the logarithm of odds ratio between the predicted promoter probability of the mutated sequence and the wild type sequence, namely

$$\textit{predicted mutation effect} = ME = logit(p_{mutated} - p_{WT}).$$

Then, an empirical one-sided *P* value was calculated for each predicted *TERT* promoter mutation effect by comparing if the predicted effect in *TERT* promoter is bigger than the predicted effect on shuffled promoter sequences at the same position and for the same type of mutation. This was done by creating 10,000 shuffled sequences (dinucleotide frequencies preserved) of the *TERT* wild type promoter. For each of these, all possible SNPs were introduced, and the predicted mutation effects were calculated for each of them as the logarithm of odds ratio against the predicted promoter probability of the wild type (shuffled) promoter sequence. For each position and mutation type, the empirical *P* value was calculated as the fraction of the predicted mutation effects on the shuffled sequences that were more extreme than the predicted effect on the wild type *TERT* promoter:

$$p - value = \frac{1}{N_{pos,mut}} \sum_i \delta_i(ME > ME_i),$$

for cases where the predicted promoter probability increases due to the mutation and

$$p - value = \frac{1}{N_{pos,mut}} \sum_i \delta_i(ME < ME_i),$$

for cases where the predicted promoter probability decreases due to the mutation. $N_{pos,mut}$ is the total number of shuffled sequences where mutation *mut* was observed at position *pos* and the summation index *i* runs over all such sequences, $\delta_i(ME<ME_i)$ is 1 if $ME<M_i$ and otherwise 0. All mutations that were non-significant according to either the empirical *P* value or the *P* value from the saturation mutagenesis experiment from ref. [15] were filtered out (threshold *P* value <0.05). The Spearman correlations of the model predictions and the saturation mutagenesis logarithmic fold-changes were computed using the "stats.spearmanr" function from SciPy (version 1.1.0, two-sided *P* value), and are shown in **Extended Data Fig. 8b-d**. The saturation mutagenesis data sets used here are TERT-HEK (HEK293T cells) and TERT-GBM (primary glioblastoma SF7996 cells) from ref. [15].

Promoter-enhancer interaction analysis using machine learning

The "binary STARR-seq" design allows looking for relatively short-range interactions between promoters and enhancers. To test for any such interactions, we trained several models with a double input CNN architecture, where the promoter and the enhancer parts of the sequence are read in with separate input convolutional bodies (a convolutional body consists of multiple layers of convolutional modules with dilated convolutions) and later connected with a fully connected layer to learn interactions between the outputs of the two convolutional bodies. Depth of the convolutional bodies was optimized with separate validation data along with other hyperparameters detailed in **Supplementary Table 8**. The rationale of this design is that the convolutional modules with dilated convolutions are capable of learning interactions within the promoters and within the enhancers, while the subsequent fully connected layer then integrates information between the promoters and the enhancers.

The search for interactions between promoters and enhancers was implemented by shuffling the training data of the models and keeping the model architecture constant, We trained double input CNN models with four different types of training data: i) The paired readout from the binary STARR-seq experiment with all possible information about interactions between promoters and enhancers intact, referred to as "binary STARR-seq CNN (paired)" in **Fig. 7c**. ii) Permutated training data, where the pairing between the promoter and the enhancer sequences is broken so that any specific interactions between the promoters and enhancers are killed, but all promoters and enhancers come from promoter+enhancer pairs that are active, referred to as "binary STARR-seq CNN (permutated)" in **Fig. 7c**. iii) Training data where only the promoter comes from active promoter+enhancer pairs and the enhancer sequences are sampled randomly from the inactive input pool of enhancer sequences, referred to as "binary STARR-seq CNN (enhancer from input)" in **Fig. 7c**. iv) Training data where only the enhancer comes from active promoter+enhancer pairs and the promoter sequences are sampled randomly from the inactive input pool of promoter sequences, referred to as "binary STARR-seq CNN (promoter from input)" in **Fig. 7c.**

Preprocessing of genomic promoters

Human promoter coordinates were obtained from the eukaryotic promoter database[63] (EPD, version 006, hg19) and sequences 100 bp upstream and 20 bp downstream of the TSS were fetched. Promoters overlapping with the extended blacklist described earlier or residing outside of chromosomes 1-22 or X were discarded. The division to training, test and validation sets for machine-learning is detailed in **Supplementary Table 7**. The genomic promoter control set (class 0) was generated by randomly drawing a balanced number of 120 bp sequences (according to the same training, test and validation split) that do not overlap with EPD promoters or regions in the extended blacklist described above. For cancer-associated mutation analysis, the genomic sequence 100 bp upstream and 20 bp downstream of the TSS for *TERT* transcript ENST00000310581.5 was downloaded from Ensembl GRCh37 release 99 Biomart. The top three mutation hotspots in the sequence as well as the recurring mutations (mutations observed in more than one patient) were obtained from ref. [16].

CAGE analysis

The 5' ends of the GP5d CAGE reads contain a 3-bp barcode (ATC) followed by a 6 bp constant sequence (CAGCAG). In addition to removing these, the next 2 bp that were mostly Gs, according to a FastQC quality control report (version 0.11.2; https://www.bioinformatics.babraham.ac.uk/projects/fastqc/), were discarded. The reads were aligned to a combined Phi X 174 + hg19 reference genomes using bwa aln[64] (version 0.7.10-r789) with parameters '-t 8 -B 11 -q 30'. Only the reads mapping to hg19 with MAPQ 30 or higher were extracted and duplicates were removed with samtools rmdup (version 1.3.1). The

strand specific 5'-end coverages of the mapped reads were computed with bedtools[41] genomecov (version 2.27.1). The active promoters were discovered from the mapped CAGE reads by clustering the 5'-ends of the reads with paraclu software[65](version 9). In total, paraclu called 7365 clusters (peaks) from the GP5d CAGE data fulfilling the following criteria: 1) Cluster is supported by more than 9 unique reads. 2) Cluster cannot be longer than 200 bp. 3) Remove clusters where the maximum 5' end density per base divided by the baseline density is less than 2. 4) Remove any cluster that is contained within a larger cluster. The active GP5d promoters, used as the test set in predicting TSS position, were defined as those EPD test set (see **Supplementary Table 7**) promoters that overlap with a GP5d CAGE peak. Thus, the TSS positions come from EPD.

Supplementary References

1 Muerdter, F. et al. Resolving systematic errors in widely used enhancer activity assays in human cells. *Nat Methods* **15**, 141-149 (2018).

2 Carninci, P. et al. Genome-wide analysis of mammalian promoter architecture and evolution. *Nat Genet* **38**, 626-635 (2006).

3 Wei, B. et al. A protein activity assay to measure global transcription factor activity reveals determinants of chromatin accessibility. *Nat Biotechnol* **36**, 521-529 (2018).

4 Lambert, S. A. et al. The Human Transcription Factors. *Cell* **172**, 650-665 (2018).

5 Li, K., Chen, Z., Kato, N., Gale, M., Jr. & Lemon, S. M. Distinct poly(I-C) and virus-activated signaling pathways leading to interferon-beta production in hepatocytes. *J Biol Chem* **280**, 16739-16747 (2005).

6 Alipanahi, B., Delong, A., Weirauch, M. T. & Frey, B. J. Predicting the sequence specificities of DNA- and RNA-binding proteins by deep learning. *Nat Biotechnol* **33**, 831-838 (2015).

7 Avsec, Z. et al. The Kipoi repository accelerates community exchange and reuse of predictive models for genomics. *Nat Biotechnol* **37**, 592-600 (2019).

8 Shrikumar, A., Greenside, P. & Kundaje, A. Learning important features through propagating activation differences. . *Proceedings of the 34th International Conference on Machine Learning* **70**, 3145–3153 (2017).

9 Shrikumar, A. et al. Technical Note on Transcription Factor Motif Discovery from Importance Scores (TF-MoDISco) version 0.5.6.5. *arXiv* **preprint**, doi: arXiv:1811.00416v00415 (2020).

10 Yin, Y. et al. Impact of cytosine methylation on DNA binding specificities of human transcription factors. *Science* **356** (2017).

11 Zhang, Y. et al. Model-based analysis of ChIP-Seq (MACS). *Genome Biol* **9**, R137 (2008).

12 Heinz, S., Romanoski, C. E., Benner, C. & Glass, C. K. The selection and function of cell type-specific enhancers. *Nat Rev Mol Cell Biol* **16**, 144-154 (2015).

13 Horn, S. et al. TERT promoter mutations in familial and sporadic melanoma. *Science* **339**, 959-961 (2013).

14 Huang, F. W. et al. Highly recurrent TERT promoter mutations in human melanoma. *Science* **339**, 957-959 (2013).

15 Kircher, M. et al. Saturation mutagenesis of twenty disease-associated regulatory elements at single base-pair resolution. *Nat Commun* **10**, 3583 (2019).

16 Zehir, A. et al. Mutational landscape of metastatic cancer revealed from prospective clinical sequencing of 10,000 patients. *Nat Med* **23**, 703-713 (2017).

17 Langmead, B. & Salzberg, S. L. Fast gapped-read alignment with Bowtie 2. *Nat Methods* **9**, 357-359 (2012).

18 Whyte, W. A. et al. Master transcription factors and mediator establish super-enhancers at key cell identity genes. *Cell* **153**, 307-319 (2013).

19 Bray, N. L., Pimentel, H., Melsted, P. & Pachter, L. Near-optimal probabilistic RNA-seq quantification. *Nat Biotechnol* **34**, 525-527 (2016).

20 Pimentel, H., Bray, N. L., Puente, S., Melsted, P. & Pachter, L. Differential analysis of RNA-seq incorporating quantification uncertainty. *Nat Methods* **14**, 687-690 (2017).

21 Palin, K. et al. Contribution of allelic imbalance to colorectal cancer. *Nat Commun* **9**, 3664 (2018).

22 Buenrostro, J. D., Wu, B., Chang, H. Y. & Greenleaf, W. J. ATAC-seq: A Method for Assaying Chromatin Accessibility Genome-Wide. *Curr Protoc Mol Biol* **109**, 21.29.21-21.29.29 (2015).

23 Li, H. & Durbin, R. Fast and accurate long-read alignment with Burrows-Wheeler transform. *Bioinformatics* **26**, 589-595 (2010).
24 Lidschreiber, K. et al. Transcriptionally active enhancers in human cancer cells. *Mol Syst Biol* **17**, e9873 (2021).
25 Schwalb, B. et al. TT-seq maps the human transient transcriptome. *Science* **352**, 1225-1228 (2016).
26 Zacher, B. et al. Accurate Promoter and Enhancer Identification in 127 ENCODE and Roadmap Epigenomics Cell Types and Tissues by GenoSTAN. *PLoS One* **12**, e0169249 (2017).
27 ENCODE Project Consortium. An integrated encyclopedia of DNA elements in the human genome. *Nature* **489**, 57-74 (2012).
28 Roadmap Epigenomics Consortium. Integrative analysis of 111 reference human epigenomes. *Nature* **518**, 317-330 (2015).
29 Jolma, A. et al. DNA-binding specificities of human transcription factors. *Cell* **152**, 327-339 (2013).
30 Nitta, K. R. et al. Conservation of transcription factor binding specificities across 600 million years of bilateria evolution. *Elife* **4**, e04837 (2015).
31 Bucher, P. Weight matrix descriptions of four eukaryotic RNA polymerase II promoter elements derived from 502 unrelated promoter sequences. *J Mol Biol* **212**, 563-578 (1990).
32 Jin, V. X., Singer, G. A., Agosto-Perez, F. J., Liyanarachchi, S. & Davuluri, R. V. Genome-wide analysis of core promoter elements from conserved human and mouse orthologous pairs. *BMC Bioinformatics* **7**, 114 (2006).
33 Grand, R. S. et al. BANP opens chromatin and activates CpG-island-regulated genes. *Nature* (2021).
34 Jolma, A. et al. DNA-dependent formation of transcription factor pairs alters their binding specificity. *Nature* **527**, 384-388 (2015).
35 Jolma, A. et al. Binding specificities of human RNA-binding proteins toward structured and linear RNA sequences. *Genome Res* **30**, 962-973 (2020).
36 Erhard, F. Estimating pseudocounts and fold changes for digital expression measurements. *Bioinformatics* **34**, 4054-4063 (2018).
37 Daley, T. & Smith, A. D. Predicting the molecular complexity of sequencing libraries. *Nat Methods* **10**, 325-327 (2013).
38 Zorita, E., Cusco, P. & Filion, G. J. Starcode: sequence clustering based on all-pairs search. *Bioinformatics* **31**, 1913-1919 (2015).
39 Wunderlich, Z. & Mirny, L. A. Different gene regulation strategies revealed by analysis of binding motifs. *Trends Genet* **25**, 434-440 (2009).
40 Li, Q., Brown, J. B., Huang, H. & Bickel, P. J. Measuring reproducibility of high-throughput experiments. *Ann Appl Stat* **5**, 1752-1779 (2011).
41 Quinlan, A. R. & Hall, I. M. BEDTools: a flexible suite of utilities for comparing genomic features. *Bioinformatics* **26**, 841-842 (2010).
42 Partridge, E. C. et al. Occupancy maps of 208 chromatin-associated proteins in one human cell type. *Nature* **583**, 720-728 (2020).
43 Heinz, S. et al. Simple combinations of lineage-determining transcription factors prime cis-regulatory elements required for macrophage and B cell identities. *Mol Cell* **38**, 576-589 (2010).
44 Bailey, T. L. STREME: Accurate and versatile sequence motif discovery. *Bioinformatics* (2021).
45 Gupta, S., Stamatoyannopoulos, J. A., Bailey, T. L. & Noble, W. S. Quantifying similarity between motifs. *Genome Biol* **8**, R24 (2007).

46 Cooper, G. M. et al. Distribution and intensity of constraint in mammalian genomic sequence. *Genome Res* **15**, 901-913 (2005).

47 Davydov, E. V. et al. Identifying a high fraction of the human genome to be under selective constraint using GERP++. *PLoS Comput Biol* **6**, e1001025 (2010).

48 Karolchik, D. et al. The UCSC Table Browser data retrieval tool. *Nucleic Acids Res* **32**, D493-496 (2004).

49 Tuupanen, S. et al. Characterization of the colorectal cancer-associated enhancer MYC-335 at 8q24: the role of rs67491583. *Cancer Genet* **205**, 25-33 (2012).

50 Lettice, L. A. et al. A long-range Shh enhancer regulates expression in the developing limb and fin and is associated with preaxial polydactyly. *Hum Mol Genet* **12**, 1725-1735 (2003).

51 Gonen, N. et al. Sex reversal following deletion of a single distal enhancer of Sox9. *Science* **360**, 1469-1473 (2018).

52 Korhonen, J., Martinmaki, P., Pizzi, C., Rastas, P. & Ukkonen, E. MOODS: fast search for position weight matrix matches in DNA sequences. *Bioinformatics* **25**, 3181-3182 (2009).

53 Hartonen, T., Kivioja, T. & Taipale, J. PlotMI: visualization of pairwise interactions and positional preferences learned by a deep learning model from sequence data. *bioRxiv* **preprint**, doi: https://doi.org/10.1101/2021.1103.1114.435285 (2021).

54 Pedregosa, F. et al. Scikit-learn: Machine Learning in Python. *J Mach Learn Res* **12**, 2825-2830 (2011).

55 Hebiri, M. & Lederer, J. How Correlations Influence Lasso Prediction. *IEEE Transactions on Information Theory* **59**, 1846-1854 (2013).

56 Granek, J. A. & Clarke, N. D. Explicit equilibrium modeling of transcription-factor binding and gene regulation. *Genome Biol* **6**, R87 (2005).

57 Yu, F. & Koltun, V. Multi-scale context aggregation by dilated convolutions. *arXiv* **preprint**, doi: arXiv:1511.07122 (2015).

58 Abadi, M. et al. TensorFlow: Large-scale machine learning on heterogeneous systems. *arXiv* **preprint**, doi: arXiv:1603.04467 (2016).

59 He, K., Zhang, Z., Ren, S. & Sun, J. Delving deep into rectifiers: Surpassing human-level performance on imagenet classification. *Proceedings of the IEEE international conference on computer vision.*, doi: 10.1109/ICCV.2015.1123 (2015).

60 Ghandi, M., Lee, D., Mohammad-Noori, M. & Beer, M. A. Enhanced regulatory sequence prediction using gapped k-mer features. *PLoS Comput Biol* **10**, e1003711 (2014).

61 Lee, D. LS-GKM: a new gkm-SVM for large-scale datasets. *Bioinformatics* **32**, 2196-2198 (2016).

62 Avsec, Z. et al. Base-resolution models of transcription-factor binding reveal soft motif syntax. *Nat Genet* **53**, 354-366 (2021).

63 Dreos, R., Ambrosini, G., Groux, R., Cavin Perier, R. & Bucher, P. The eukaryotic promoter database in its 30th year: focus on non-vertebrate organisms. *Nucleic Acids Res* **45**, D51-D55 (2017).

64 Li, H. & Durbin, R. Fast and accurate short read alignment with Burrows-Wheeler transform. *Bioinformatics* **25**, 1754-1760 (2009).

65 Frith, M. C. et al. A code for transcription initiation in mammalian genomes. *Genome Res* **18**, 1-12 (2008).
