# Supplementary material for: Sequence determinants of human gene regulatory elements
Source: Nat Genet. 2022 Feb 21;54(3):283–94. doi: 10.1038/s41588-021-01009-4 (PMC8920891; doi:10.1038/s41588-021-01009-4)
Supplement: Supplementary file 2 — Reporting Summary [file 41588_2021_1009_MOESM2_ESM.pdf]

nature research

Corresponding author(s): Jussi Taipale

Last updated by author(s): Nov 17, 2021

Reporting Summary

Nature Research wishes to improve the reproducibility of the work that we publish. This form provides structure for consistency and transparency in reporting. For further information on Nature Research policies, see our Editorial Policies and the Editorial Policy Checklist.

Statistics

For all statistical analyses, confirm that the following items are present in the figure legend, table legend, main text, or Methods section.

n/a	Confirmed	
☐	☒	The exact sample size (*n*) for each experimental group/condition, given as a discrete number and unit of measurement
☐	☒	A statement on whether measurements were taken from distinct samples or whether the same sample was measured repeatedly
☐	☒	The statistical test(s) used AND whether they are one- or two-sided<br>*Only common tests should be described solely by name; describe more complex techniques in the Methods section.*
☐	☒	A description of all covariates tested
☐	☒	A description of any assumptions or corrections, such as tests of normality and adjustment for multiple comparisons
☐	☒	A full description of the statistical parameters including central tendency (e.g. means) or other basic estimates (e.g. regression coefficient) AND variation (e.g. standard deviation) or associated estimates of uncertainty (e.g. confidence intervals)
☐	☒	For null hypothesis testing, the test statistic (e.g. *F*, *t*, *r*) with confidence intervals, effect sizes, degrees of freedom and *P* value noted<br>*Give P values as exact values whenever suitable.*
☒	☐	For Bayesian analysis, information on the choice of priors and Markov chain Monte Carlo settings
☒	☐	For hierarchical and complex designs, identification of the appropriate level for tests and full reporting of outcomes
☐	☒	Estimates of effect sizes (e.g. Cohen's *d*, Pearson's *r*), indicating how they were calculated

Our web collection on statistics for biologists contains articles on many of the points above.

Software and code

Policy information about availability of computer code

Data collection	No specialized software was used for data acquisition.
Data analysis	The following previously described software were used in the data analysis and are described and cited in the Methods: ChIP-seq reads were aligned to hg19 genome with bowtie2 software (version 2.2.4). All peak calling was done with MACS2 (version 2.1.1) software. Bedtools (versions 2.25.0 and 2.29.2 when using -wao option) was used for genome arithmetics. Super-enhancers were calculated using the ROSE pipeline (version 0.1). ATAC-seq fastq files were processed using an in-house pipeline comprising of following pieces of software: TrimGalore (version 0.4.3), BWA aligner (version 0.7.15), Picard (version 2.9.2) and broad-peak calling by MACS2 (version 2.1.1). Fold change estimates for count data were calculated using the PsiLFC function in R package lfc (version 0.2.1). Random enhancer STARR-seq paired end reads were combined using FLASH program version 1.2.11. For library complexity estimation, random STARR-seq library sequences were first clustered using Starcode program version 1.3. Both genomic and random STARR-seq library complexities were estimated using Preseq program version 2.0.0. Genomic STARR-seq reads were aligned to hg19 genome using bowtie2 (version 2.2.4) and peaks called with MACS2 (version 2.1.1) and IDR (version 2.0.3) programs. De novo motif mining for ATAC-seq and ChIP-seq peaks and functional enhancers was performed with HOMER software (version 4.10.3). From the sequences enriched by the STARR-seq experiments from random enhancer, random promoter random enhancer data and ATI assay and specific positions in the TSS-aligned random promoter STARR-seq sequences the de novo motif mining was done using the Autoseed program (as in Nitta et al, Elife 4, e04837, 2015). Additional de novo motif mining from random enhancer STARR-seq and random sequences scored by the random enhancer STARR-seq CNN model was conducted using STREME program (version 5.3.3). STARR-seq derived motifs were compared to known TF motifs using TOMTOM (versions 5.0.5 and 5.3.3 used for motif and random library, respectively). Human genome (hg19) coordinates of the SOX9 enhancer were calculated with the USCS genome LiftOver tool from mouse mm9 coordinates (from http://genome.ucsc.edu/cgi-bin/hgLiftOver). Random promoter-enhancer pairs were filtered for adapter sequences using cutadapt software version 1.9.1 and sequences mapping to the plasmid backbone according to bowtie2 were removed. Cutadapt was also used in mapping the TSS position based on template switching. PWM match scores to DNA were calculated using MOODS software (version 1.9.3). Logistic regression classifiers were implemented using the LogisticRegression function from scikit-learn library (version 0.21.3). Lasso regression models were implemented using LassoCV method in scikit-learn (version 0.24.1). Convolutional neural network (CNN)

classifiers were implemented using Keras (version 2.2.4-tf) with TensorFlow 1.14.0 backend. BWA aligner version 0.7.15-r1142-dirty was used in creating the extended blacklist for genomic machine learning analyses. DeepLift (version 0.6.12) was used to visualize the features the promoter STARR-seq trained CNN used for predicting promoter activity. Motif discovery from the random enhancer STARR-seq CNN model was conducted using TF-MoDISco program (version 0.5.14.1). TOMTOM version 5.4.1 was used to compare TF binding motifs learned by the CNN to de novo motifs from random enhancer STARR-seq sequences. FastQC software (version 0.11.2) was used for CAGE data quality control. CAGE reads were aligned to combined Phi X 174 and hg19 genomes with the BWA aligner (version 0.7.10-r789) and the CAGE mapped read clustering was done using paraclu software version 9. RNA-seq differential expression analysis was conducted using kallisto and sleuth program versions 0.46.1 and 0.30.0, respectively. Gene set enrichment analysis was done using preranked analysis with GSEA (version 4.1.0). SciPy (version 1.1.0) functions stats.ttest_rel and stats.spearmanr were used to compute paired Student's t-test and Spearman correlations, respectively.

All custom code central to the study used in the data analysis is described in the Methods and available at Zenodo with accession 10.5281/zenodo.5159644 as mentioned in the Code Availability Statement. The code is divided into six packages: 1) StarrTrack: Java code for processing motif STARR-seq library data; 2) AssignTSS: Perl script that assigns the TSS positions based on the template-switch data; 3) CountMotifPairs: Two R scripts that count motif match pairs from promoter enhancer pairs and motif match spacings and orientations from enhancers, respectively; 4) trainCNN: Python scripts for training the CNN classifiers; 5) trainLogReg: Python scripts for training the logistic regression classifiers; 6) trainReg: Python scripts for training the differential expression predictor.

For manuscripts utilizing custom algorithms or software that are central to the research but not yet described in published literature, software must be made available to editors and reviewers. We strongly encourage code deposition in a community repository (e.g. GitHub). See the Nature Research guidelines for submitting code & software for further information.

Data

Policy information about availability of data

All manuscripts must include a data availability statement. This statement should provide the following information, where applicable:

- Accession codes, unique identifiers, or web links for publicly available datasets
- A list of figures that have associated raw data
- A description of any restrictions on data availability

All sequence data generated in this study is available under GEO accession GSE180158. All pre-trained machine learning models are available at Zenodo with accession 10.5281/zenodo.5101420. Training, test, and validation data sets for the CNN models are available at Zenodo with accession 10.5281/zenodo.5101420. Genome browser session is available at UCSC portal with tracks for all genomic data sets generated in this study (https://genome.ucsc.edu/s/kivioja/Sahu_et_al_Human_regulatory_elements).
ENCODE blacklisted genomic regions for hg19 (accession ENCSR636HFF) were downloaded from ENCODE, RepeatMasker file for hg19 was downloaded using the UCSC table browser. The Eukaryotic Promoter Database (EPD) for human TSSs can be found from: https://epd.epfl.ch/EPDnew_database.php. In addition, transcript annotations downloaded from Ensembl (GRCh37, release 101) were used. The saturation mutagenesis results of the TERT promoter can be found from: https://doi.org/10.17605/OSF.IO/75B2M. GERP conservation scores for the hg19 reference genome can be found from: http://mendel.stanford.edu/SidowLab/downloads/gerp/. The following datasets were downloaded from the ENCODE portal: ATAC-seq (ENCSR042AWH, replicate 1), histone modification ChIP-seq experiments for H3K27ac (ENCSR000AMO), H3K27me3 (ENCSR000AOL), H3K9me3 (ENCSR000ATD), and H3K4me1 (ENCFF424GUI), as well as ChIP-seq data sets for TP53 (ENCSR980EGJ), MED1 (ENCFF493UFO), and MED13 (ENCFF003HBS). ChIP-seq peak sets were also downloaded from GEO accession GSE104247. Super-enhancers for HepG2 were downloaded from http://www.licpathway.net/sedb. Previously published RNA-seq data used in the study is available at EGA (accession https://ega-archive.org/studies/EGAS00001002966).
Accession numbers for all data sets used in this study are also mentioned in Data Availability Statement.

Field-specific reporting

Please select the one below that is the best fit for your research. If you are not sure, read the appropriate sections before making your selection.

☒ Life sciences ☐ Behavioural & social sciences ☐ Ecological, evolutionary & environmental sciences

For a reference copy of the document with all sections, see nature.com/documents/nr-reporting-summary-flat.pdf

Life sciences study design

All studies must disclose on these points even when the disclosure is negative.

Sample size

The achievable sample sizes in STARR-seq experiments are limited by cloning, cell culture and other steps of the large-scale experiments. The ultra-high complexities of the input libraries were verified using state-of-art computational tools as described in Methods. The reliability of the biological conclusions made from the achievable sample sizes was ensured by i) manual inspection of the genomic peaks, ii) replicate comparisons, iii) statistical testing, and iv) power analysis where appropriate. The numbers of the sequencing reads used in each analysis are listed in Supplementary Tables.

Data exclusions

In all experiments, sequencing reads were filtered using defined quality parameters and the genomic reads overlapping with the ENCODE blacklisted regions were excluded. IRF3 ChIP-seq signals were weak in HepG2 cells based on the lack of peak overlaps with motif sites and the manual inspection of the top peaks, and they were excluded from the downstream analysis.

Replication

STARR-seq experiments were performed in two replicates with random enhancer libraries in GP5d and HepG2 cells and with random promoter-enhancer libraries in GP5d cells; genomic STARR-seq experiments were performed in two replicates in HepG2 cells and in four different conditions in GP5d cells (wild type and TP53-null GP5d cells using both methylated and non-methylated input DNA libraries); experiments with random promoter-enhancer libraries in HepG2 and RPE1 cells had no replicates. RNA-seq experiments were performed in three replicates, ChIP-seq and ATAC-seq from one sample per condition.

Similar conclusions were obtained from independently generated data sets and the replicate experiments showed high concordance (Extended Data Figures 2a and 5a).

Randomization	Not applicable. Experiments were performed on uniform biological material i.e. commercial cell lines, so randomization for different experimental groups was not relevant for this study. Randomization of the sequencing reads for the purposes of specific statistical or computational analysis, such as for training, test, and validation sets used in the machine learning analyses, is described for each specific analysis in the Methods section.
Blinding	Analyses were performed using computational algorithms for large data sets of sequencing reads, and thus blinding of the investigators was not relevant for this study.

Reporting for specific materials, systems and methods

We require information from authors about some types of materials, experimental systems and methods used in many studies. Here, indicate whether each material, system or method listed is relevant to your study. If you are not sure if a list item applies to your research, read the appropriate section before selecting a response.

Materials & experimental systems

n/a	Involved in the study	
☐	☒	Antibodies
☐	☒	Eukaryotic cell lines
☒	☐	Palaeontology and archaeology
☒	☐	Animals and other organisms
☒	☐	Human research participants
☒	☐	Clinical data
☒	☐	Dual use research of concern

Methods

n/a	Involved in the study	
☐	☒	ChIP-seq
☒	☐	Flow cytometry
☒	☐	MRI-based neuroimaging

Antibodies

Antibodies used

H3K27ac, H3K9me3, and H3K27me3 (C15410196, C15410193, and C15410195, Diagenode, respectively); FOXA1 (#ab23738, Abcam); p53, HNF4a, IRF3 and CTCF [sc-126x (DO-1), sc-8987x (H-171), sc-33641x (SL-12), and sc-15914x (C-20), Santa Cruz, respectively]; SMC1 (A300-055A, Bethyl lab), and normal mouse, rabbit and goat IgG from Santa Cruz (#sc-2025, #sc-2027 and #sc-2028, respectively)

Validation

The anti-H3K27ac polyclonal antibody is raised in rabbit against the region of histone H3 containing the acetylated lysine 27 (H3K27ac), using a KLH-conjugated synthetic peptide. It is recommended for detecting H3K27ac in ChIP experiments in human by the manufacturer, and there is validation data and >50 citations available for this antibody on the manufacturer’s website (https://www.diagenode.com/en/p/h3k27ac-polyclonal-antibody-premium-50-mg-18-ml).
The anti-H3K9me3 polyclonal antibody is raised in rabbit against the region of histone H3 containing the trimethylated lysine 9 (H3K9me3), using a KLH-conjugated synthetic peptide. It is recommended for detecting H3K9me3 in ChIP experiments in human by the manufacturer, and there is validation data and >30 citations available for this antibody on the manufacturer’s website (https://www.diagenode.com/en/p/h3k9me3-polyclonal-antibody-premium-50-mg).
The anti-H3K27me3 polyclonal antibody is raised in rabbit against the region of histone H3 containing the trimethylated lysine 27 (H3K27me3), using a KLH-conjugated synthetic peptide. It is recommended for detecting H3K27me3 in ChIP experiments in human by the manufacturer, and there is validation data and >60 citations available for this antibody on the manufacturer’s website (https://www.diagenode.com/en/p/h3k27me3-polyclonal-antibody-premium-50-mg-27-ml).
The anti-FOXA1 polyclonal antibody is raised in rabbit against the synthetic peptide within human FOXA1 aa 450 to the C-terminus (C terminal) conjugated to keyhole limpet haemocyanin. It is recommended for detecting FOXA1 in ChIP experiments in human by the manufacturer, and there is validation data and >100 citations available for this antibody on the manufacturer’s website (https://www.abcam.com/foxa1-antibody-chip-grade-ab23738.html?productWallTab=ShowAll).
The anti-p53 (DO-1) is a mouse monoclonal antibody raised against a short amino acid sequence containing Ser315 phosphorylated p53 of human origin. It is a ChIP-grade antibody recommended for detecting human p53 by the manufacturer. There is validation data on the manufacturer's website, including 5780 citations for previous literature (https://www.scbt.com/p/p53-antibody-do-1).
The anti-IRF3 (SL-12) is a mouse monoclonal antibody raised against recombinant IRF-3 fusion protein corresponding to human IRF-3 (amino acids 56-427). It is a ChIP-grade antibody recommended for detecting human IRF3 by the manufacturer. There is validation data on the manufacturer's website, including 46 citations for previous literature (https://www.scbt.com/p/irf-3-antibody-sl-12).
The anti-HNF4a (H171) is a rabbit polyclonal antibody raised against an epitope corresponding to amino acids 295-465 mapping at the C-terminus of HNF-4α of human origin. It is a ChIP-grade antibody recommended for detecting human HNF4a by the manufacturer. There is validation data on the manufacturer's website, including 71 citations for previous literature (https://www.scbt.com/p/hnf-4alpha-antibody-h-171).
The anti-CTCF (C-20) is a goat polyclonal antibody raised against an epitope mapping near the C-terminus of CTCF of human origin. It is a ChIP-grade antibody recommended for detecting human CTCF by the manufacturer with >20 citations available for this antibody (https://www.citeab.com/antibodies/782679-sc-15914-ctcf-antibody-c-20).
The anti-SMC1 is a rabbit polyclonal antibody raised against an epitope mapping between 1175 and C-terminus of SMC1 of human origin. It is recommended for detecting human SMC1 by the manufacturer with >180 citations available for this antibody (https://www.bethyl.com/product/A300-055A/SMC1+Antibody).
Normal IgGs from Santa Cruz are commonly used controls.

Eukaryotic cell lines

Policy information about cell lines

Cell line source(s)	Colon cancer cell line GP5d (Sigma #95090715), liver cancer cell line HepG2 (ATCC #HB-8065), retinal pigmented epithelial cell line hTERT-RPE1 (ATCC #CRL-4000).
Authentication	All cell lines were directly obtained from trusted vendors (ATCC, Sigma) and not from other laboratories, and only low-passage cells were used in the experiments. Cell lines were not authenticated.
Mycoplasma contamination	All cell lines tested negative for mycoplasma contamination upon purchase and were routinely monitored as per standard good laboratory practices.
Commonly misidentified lines (See ICLAC register)	Cell lines used in this study are not on the list of commonly misidentified cell lines

ChIP-seq

Data deposition

☒ Confirm that both raw and final processed data have been deposited in a public database such as GEO.

☒ Confirm that you have deposited or provided access to graph files (e.g. BED files) for the called peaks.

Data access links
May remain private before publication.

Raw and processed reads are available under GEO accession GSE180158.

Files in database submission

H3K27ac_Mock_HepG2_ChIP_10_S10_L1-2.fastq.gz
p53_Mock_HepG2_ChIP_7_S7_L1-2.fastq.gz
IRF3_Mock_HepG2_ChIP_13_S13_L1-2.fastq.gz
H3K27ac_SS-NLE_HepG2_ChIP_11_S11_L1-2.fastq.gz
p53_SS-NLE_HepG2_ChIP_8_S8_L1-2.fastq.gz
IRF3_SS-NLE_HepG2_ChIP_14_S14_L1-2.fastq.gz
H3K27ac_5-FU_HepG2_ChIP_12_S12_L1-2.fastq.gz
p53_5-FU_HepG2_ChIP_9_S9_L1-2.fastq.gz
IRF3_5-FU_HepG2_ChIP_15_S15_L1-2.fastq.gz
rIgG_Mock_HepG2_ChIP_1_S1_L1-2.fastq.gz
rIgG_SS-NLE_HepG2_ChIP_2_S2_L1-2.fastq.gz
rIgG_5-FU_HepG2_ChIP_3_S3_L1-2.fastq.gz
mIgG_Mock_HepG2_ChIP_4_S4_L1-2.fastq.gz
mIgG_SS-NLE_HepG2_ChIP_5_S5_L1-2.fastq.gz
mIgG_5-FU_HepG2_ChIP_6_S6_L1-2.fastq.gz
Input_HepG2_ChIP_21_S21_L1-2.fastq.gz
GP5d-Ctrl-p53-DO-1_S2_R1_001.fastq.gz
GP5d-5-FU_p53-DO-1_S3_R1_001.fastq.gz
GP5d-5-FU_mIgG_S1_R1_001.fastq.gz
CTCF2_GP5d_S11_R1_001.fastq.gz
gIgG-GP5D_S1_R1_001.fastq.gz
GP5D-H3K27ac_S45_R1_001.fastq.gz
GP5D-rIgG_S39_R1_001.fastq.gz
SMC1-GP5D_S8_R1_001.fastq.gz
GP5D-H3K9me3_S63_R1_001.fastq.gz
rIgG-GP5D_S2_R1_001.fastq.gz
FOXA1_2_GP5d_S12_R1_001.fastq.gz
HNF4a2_GP5d_S13_R1_001.fastq.gz
rIgG2_GP5D_S8_R1_001.fastq.gz
mIgG-GP5D_S3_R1_001.fastq.gz
GP5d-1_H3K27me3_S10_R1_001.fastq.gz
GP5d-rIgG-hm_S8_R1_001.fastq.gz
H3K27ac_Mock_HepG2_vs_Input_peaks.narrowPeak
p53-Mock_HepG2_vs_Input_peaks.narrowPeak
IRF3-Mock_HepG2_vs_Input_peaks.narrowPeak
H3K27ac_SS-NLE_HepG2_vs_Input_peaks.narrowPeak
p53-SS-NLE_HepG2_vs_Input_peaks.narrowPeak
IRF3-SS-NLE_HepG2_vs_Input_peaks.narrowPeak
H3K27ac_5-FU_HepG2_vs_Input_peaks.narrowPeak
p53-5-FU_HepG2_vs_Input_peaks.narrowPeak
IRF3-5-FU_HepG2_vs_Input_peaks.narrowPeak
Ctrl_TP53_vs_mIgG_peaks.narrowPeak
5FU_TP53_vs_mIgG_peaks.narrowPeak
CTCF2_GP5D_vs_gIgG2_peaks.narrowPeak
H3K27ac_vs_rIgG_GP5d_peaks.narrowPeak
SMC1-GP5D_vs_rIgG_peaks.narrowPeak
H3K9me3_GP5D_broad_vs_rIgG_peaks.broadPeak

FOXA1_2_GP5D_vs_rIgG2_peaks.narrowPeak
HNF4a_2_GP5D_vs_rIgG2_peaks.narrowPeak
GP5d-1_H3K27me3_vs_rIgG-hm_peaks.broadPeak

Genome browser session (e.g. UCSC)

https://genome.ucsc.edu/s/kivioja/Sahu_et_al_Human_regulatory_elements

Methodology

Replicates

The main findings of the study are based on the STARR-seq experiments, and ChIP-seq data for was used to support the conclusions. Due to the large number of different ChIP-seq data sets needed, we only used one replicate for each condition.

Sequencing depth

Mapped reads for GP5d-TP53: total = 63021885; unique = 44989603
Mapped reads for GP5d-mIgG control for TP53: total = 40330794; unique = 28699379
Mapped reads for GP5d-FOXA1: total = 47301290; unique = 4007819
Mapped reads for GP5d-rIgG control for FOXA1: total = 49338820; unique = 43678360
Mapped reads for GP5d-CTCF: total = 32829873; unique = 28644653
Mapped reads for GP5d-gIgG control for CTCF: total = 26247818; unique = 23128132
Mapped reads for GP5d-H3K27ac: total = 61776858; unique = 50807468
Mapped reads for GP5d-rIgG control for H3K27ac: total = 37885351; unique = 32031305
Mapped reads for GP5d-H3K9me3: total = 20163128; unique = 17797650
Mapped reads for GP5d-rIgG control for H3K9me3: total = 49338820; unique = 43678360
Mapped reads for GP5d-H3K27me3: total = 35415410; unique = 24816970
Mapped reads for GP5d-rIgG control for H3K27me3: total = 31003987; unique = 22463136
Mapped reads for GP5d-HNF4a: total = 20180294; unique = 15124792
Mapped reads for GP5d-rIgG control for HNF4a: total = 48864528; unique = 30269040
Mapped reads for GP5d-SMC1: total = 41817385; unique = 36074245
Mapped reads for GP5d-rIgG control for SMC1: total = 49338820; unique = 43678360
Mapped reads for HepG2-p53-SS-NLE_HepG2_vs_Input : total = 23008852; unique = 18888219
Mapped reads for HepG2-Input control for p53-SS-NLE_HepG2: total = 30690201; unique = 26599619
Mapped reads for HepG2-p53-Mock_HepG2_vs_Input : total = 24501270; unique = 19517558
Mapped reads for HepG2-Input control for p53-Mock_HepG2: total = 30690201; unique = 26599619
Mapped reads for HepG2-p53-5-FU_HepG2_vs_Input : total = 16674402; unique = 10798674
Mapped reads for HepG2-Input control for p53-5-FU_HepG2: total = 30690201; unique = 26599619
Mapped reads for HepG2-IRF3-SS-NLE_HepG2_vs_Input : total = 16165814; unique = 7363592
Mapped reads for HepG2-Input control for IRF3-SS-NLE_HepG2: total = 30690201; unique = 26599619
Mapped reads for HepG2-IRF3-Mock_HepG2_vs_Input: total = 11264345; unique = 5765858
Mapped reads for HepG2-Input control for IRF3-Mock_HepG2: total = 30690201; unique = 26599619
Mapped reads for HepG2-IRF3-5-FU_HepG2_vs_Input: total = 19588013; unique = 9655159
Mapped reads for HepG2-Input control for IRF3-5-FU_HepG2 total = 30690201; unique = 26599619
Mapped reads for HepG2-H3K27ac_SS-NLE_HepG2_vs_Input: total = 30963011; unique = 22680118
Mapped reads for HepG2-Input control for H3K27ac_SS-NLE_HepG2 total = 30690201; unique = 26599619
Mapped reads for HepG2-H3K27ac_Mock_HepG2_vs_Input: total = 40104064; unique = 28382584
Mapped reads for HepG2-Input control for H3K27ac_Mock_HepG2 total = 30690201; unique = 26599619
Mapped reads for HepG2-H3K27ac_5-FU_HepG2_vs_Input: total = 31156671; unique = 21425466
Mapped reads for HepG2-Input control for H3K27ac_5-FU_HepG2 total = 30690201; unique = 26599619

Antibodies

H3K27ac, H3K9me3, and H3K27me3 (C15410196, C15410193, and C15410195, Diagenode, respectively); FOXA1 (#ab23738, Abcam); p53, HNF4a, IRF3 and CTCF (sc-126x, sc-8987x, sc-33641x, and sc-15914x, Santa Cruz, respectively); SMC1 (A300-055A, Bethyl lab), and normal mouse, rabbit and goat IgG from Santa Cruz (#sc-2025, #sc-2027 and #sc-2028, respectively)

Peak calling parameters

Peak calling was performed using MACS2 with default parameters (narrow peaks were called for all samples except broad peaks for repressive histone modifications).

Data quality

Correct TF motifs were discovered from the ChIP-seq peaks for all samples except IRF3 ChIP-seq in HepG2, and thus IRF3 data was not used in further analysis.

Software

Bowtie2 (Langmead, & Salzberg, Nat Methods 9, 357-359, 2012)
MACS2 (Zhang et al., Genome Biol. 9, pp. R137, 2008)
