# Supplementary material for: Sequence determinants of human gene regulatory elements
Source: Nat Genet. 2022 Feb 21;54(3):283–94. doi: 10.1038/s41588-021-01009-4 (PMC8920891; doi:10.1038/s41588-021-01009-4)
Supplement: Supplementary file 3 — Peer Review Information [file 41588_2021_1009_MOESM3_ESM.pdf]

natureresearch

Peer Review Information

Journal: Nature Genetics
Manuscript Title: Sequence determinants of human gene regulatory elements
Corresponding author name(s): Professor Jussi Taipale

Reviewer Comments & Decisions:

Decision Letter, initial version:

27th May 2021

Dear Jussi,

Your Article, "Sequence determinants of human gene regulatory elements" has now been seen by 3 referees. I apologize for the long review process. Unfortunately, reviewer #4 has not submitted a timely report despite our multiple chase emails. We have now decided to proceed based on the current reviews.

You will see from the reviewers' comments copied below that while they find your work of considerable potential interest, they have raised quite substantial concerns that must be carefully and thoroughly addressed. In light of these comments, we cannot accept the manuscript for publication, but would be interested in considering a revised version that addresses these serious concerns.

Reviewer #1 thinks that the dataset is valuable and that the findings are potentially exciting but that the manuscript is very complex. The reviewer feels that it should either be split into several studies or that you need to restructure it; we recommend the latter. Importantly, the reviewer asks for all the data/methods to be adequately provided/explained. They raise some salient technical issues but these are not necessarily damaging.
Reviewer #2 is positive about this study technically and thinks that it is thought-provoking. They have

a number of very insightful and constructive comments; some are about data interpretation/display, others about additional analyses.
Reviewer #3's comments are not very detailed, unfortunately. The reviewer thinks that the manuscript is "impenetrable", which is reminiscent of what Reviewer #1 said. They note that the claims are potentially important but seem unsubstantiated at this stage; the reviewer doesn't provide details, though.

We hope you will find the referees' comments useful as you decide how to proceed. If you wish to submit a substantially revised manuscript, please bear in mind that we will be reluctant to approach the referees again in the absence of major revisions.

If you choose to revise your manuscript taking into account all reviewer and editor comments, please highlight all changes in the manuscript text file. At this stage we will need you to upload a copy of the manuscript in MS Word .docx or similar editable format.

We are committed to providing a fair and constructive peer-review process. Do not hesitate to contact me if there are specific requests from the reviewers that you believe are technically impossible or unlikely to yield a meaningful outcome.

If revising your manuscript:

*1) Include a "Response to referees" document detailing, point-by-point, how you addressed each referee comment. If no action was taken to address a point, you must provide a compelling argument. This response will be sent back to the referees along with the revised manuscript.

*2) If you have not done so already please begin to revise your manuscript so that it conforms to our Article format instructions, available here.
Refer also to any guidelines provided in this letter.

*3) Include a revised version of any required Reporting Summary:
https://www.nature.com/documents/nr-reporting-summary.pdf
It will be available to referees (and, potentially, statisticians) to aid in their evaluation if the manuscript goes back for peer review.
A revised checklist is essential for re-review of the paper.

Please be aware of our guidelines on digital image standards.

You may use the link below to submit your revised manuscript and related files:

[REDACTED]

Note: This URL links to your confidential home page and associated information about manuscripts you may have submitted, or that you are reviewing for us. If you wish to forward this email to co-authors, please delete the link to your homepage.

If you wish to submit a suitably revised manuscript we would hope to receive it within 6 months. If

you cannot send it within this time, please let us know. We will be happy to consider your revision so long as nothing similar has been accepted for publication at Nature Genetics or published elsewhere. Should your manuscript be substantially delayed without notifying us in advance and your article is eventually published, the received date would be that of the revised, not the original, version.

Please do not hesitate to contact me if you have any questions or would like to discuss the required revisions further.

Nature Genetics is committed to improving transparency in authorship. As part of our efforts in this direction, we are now requesting that all authors identified as ‘corresponding author’ on published papers create and link their Open Researcher and Contributor Identifier (ORCID) with their account on the Manuscript Tracking System (MTS), prior to acceptance. ORCID helps the scientific community achieve unambiguous attribution of all scholarly contributions. You can create and link your ORCID from the home page of the MTS by clicking on ‘Modify my Springer Nature account’. For more information please visit please visit www.springernature.com/orcid.

Thank you for the opportunity to review your work.

Sincerely,

Tiago

Tiago Faial, PhD
Senior Editor
Nature Genetics
https://orcid.org/0000-0003-0864-1200

Reviewers' Comments:

Reviewer #1:
Remarks to the Author:
In their manuscript "Sequence determinants of human gene regulatory elements" Biswajyoti Sahu et al. performed a number of very interesting reporter assays (STARR-Seq) and in vitro binding (ATI) experiments, as well as created a number of supporting RNA-seq, ATAC-seq and ChIP-seq data sets. The result is a very interesting (but also very complex) read out of regulatory elements in (mostly) two cancer cell-lines (HepG2 and GP5d). There are multiple different STARR-Seq assays in this study and the authors branch out into studying effects of methylation, the interaction of regulatory sequences ("promoter"-"enhancer" interactions) as well as the effects of a specific TF knockout (TP53). This all is supplemented by an in-depth modeling analysis using regression, gapped-kmer or CNN models.

Trying to put all that into a single study comes at the cost of oversimplifying a story in the main text

(without a comprehensive discussion of individual limitations) while providing a very long method description that is too complex to follow for anyone who is not acquainted with the study. My first suggestion would be to "divide and conquer" and to publish this work as a set of two to three articles in the "same issue" of a journal. I believe that we are looking at some very interesting work here, which is really not well represented the way it is currently written up. If splitting up is no option, I would highly recommend to write a structured supplemental note where the individual experiments are motivated and outlined, and most importantly their limitations discussed.

Another high-level criticism is the availability of models and raw as well as processed data. While the authors announce that models and raw data will be released, this is something that should be done before submitting revisions of the manuscript. The reviewers need to see that all data was deposited with the appropriate meta-information. Specifically, all data should be deposited through NCBI GEO to allow that also processed data is released. Similarly, data/methods cannot be referenced with a manuscript "in preparation" (L733). Either this manuscript is available as pre-print or the information is provided as "personal communication", in which case a sufficient description has to be provided to the reader/reviewers.

As briefly mentioned above, the current version of the main manuscript appears to me more of a big picture story telling; while many important details are only available in the methods and a discussion of experimental limitations is missing. In this context it seems most important that even if trying to simplify for a broad readership, a correct picture is conveyed. For me issues arise for the following topics:

(1) It is essential to mention the applied experimental methods early on (i.e. in the abstract) and the authors should refrain from comparing to an "aggregated length" of tested sequences throughout the manuscript. The authors tested millions of short sequences, they never tested long sequences and a concatenated length is of no relevance here as these sequences did not act in that aggregate form.

(2) The authors talk about "evolved" sequences, but what they mean is that they tested random sequences and that a proportion of those showed activity. This has nothing to do with molecular evolution. In some contexts, it might be sufficient to talk about motifs/sequences that are enriched/overrepresented among those activating expression.

(3a) The authors are doing an unusual normalization of STARR-Seq data, which is typically used as signal over background measure. Due to the high library complexity, sequencing the genomic background is uneconomical (L846ff) and the authors try to normalize differently (e.g. by comparing motif enrichments vs a random sample of the input). This however cannot correct for artifacts in the STARR-Seq data or for more subtle effects like GC content. The authors need to discuss these technical aspects of their data (and not just in the methods).

(3b) STARR-Seq (like also MPRA/CRE-seq approaches) seem inherently biased (e.g. due to a minimal promoter) towards activating rather than repressing effects. How does this impact your study and the drawn conclusions?

(3c) STARR-Seq assays also cause a strong cellular "immune" response and you seem to be dealing with a rather high number of plasmids per cell (L876), how does that impact your results? What about plasmid silencing?

(Very incomplete list of) minor/other comments:

- L67: provide length/context of TF binding motif MPRA

- L110: active TF identification (ATI) need more of an introduction

- L220: discuss how this relates to "primed" enhancers and whether these are active enhancers in other cell-types that only become active because they are moved into an active chromatin context.

- L347: "Molecular evolution approach" -- Is there a prior definition of what this entails? I am having issues with the term evolution for this study.

- L401ff: The statement about TFs as basic unit makes sense and is probably not challenged. The argument whether "regulatory elements are not atomic units of gene expression" seems to be semantic one, especially as the authors argue for an additive effect of multiple TFs with some activation limit. Considering that the authors are testing these TFs in (very) close proximity (due to technical limitations), it seems an unsupported statement that there would be no length-restricted local neighborhood within the TFs act additively on expression regulation and that these should not be called regulatory elements.

- L522ff: Details about amount of DNA and cycles missing

- L539ff: Amount of material, estimate of oligo complexity

- L558ff: It remains unclear to me how the reporter gene is inserted in between "promoter" and "enhancer" random oligo regions, the size of 555 bp seems too short to include it. Please clarify.

- L647ff: Template switching seems to be the required method for reading the "promoter"-"enhancer" STARR-seq library. This needs to be clearer. Also Fig4a (L1890) needs a complete description of the process. You should also bring the corresponding analysis methods closer in text (i.e. L995ff and L1010ff).

- L729: Description of bioinformatics analysis of data as well as of enriched motifs/known factors seems to be missing.

- L830ff: In a previous analysis you used the LFC package, why are you not using it here?

- L1000: "Hamming distance of less than 3". You are probably trying to handle synthesis and sequencing errors here. Please provide more insight into why you are doing things and why you are picking these thresholds.

- L1070: You are using Ensembl (L) and EPD (L1031) for TSS annotation across different analyses, you therefore need to specify the source in each case.

- L1098: Why adding a pseudo count of 10?

- L1150ff: Regularization does not protect from issues with correlated features. It might slightly help the interpretation, but it does not solve the actual issue.

- L1843: Not STARR-seq on its own, but the combination of readouts reveals the different types of regulatory elements. Especially as STARR-Seq does not show signal for some otherwise defined elements.

Textual errors:
- L187: [the] GP5d genome
- L702: correct sentence around "lysed"
- L867: which -> with
- L1817: delete "relatively"

Overall, this is a great set of experiments and a very exciting resource. I hope the authors can make all this a bit better accessible to the reader. Even as a very interested reader, I could only get through a very thin layer of this work.

Reviewer #2:
Remarks to the Author:
The authors present an interesting study examining the transcriptional activity of 400 billion DNA bases in three cellular contexts using STARR-seq. Collectively, their study examines ~100 times the sequence capacity of the human genome, and therefore serves as a strong platform for a large-scale unbiased view of the sequence determinants of human gene regulatory element activity.

Several key observations are presented based on the data:
1. There are three classes of enhancers, with different mechanisms and motif content
2. Transcription factors (TFs) generally have (weak) additive effects
3. Only a few TFs are strongly active in a cell type, with the strongest differences between cells representing known TFs that are important for specification and/or function of the specific lineages
4. Only a subset of TFs have TSS positional dependency
5. There is no evidence for 'beyond additive' interactions between promoters and enhancers

Overall this is a well designed and well executed study. I expect it will be very interesting for the community and will spark a good amount of discussion and debate. That said, I have several comments, most of which involve data interpretation.

Analysis/presentation/interpretation issues:
1. "The most active motifs displayed similar activities when placed in different sequence contexts, and between experiments using two different basal promoters, δ1-crystallin and CpG-free EF1α promoters" – not sure I totally agree with that 2nd part. For the top motifs (p53, IRF HT2 in Ext Data Fig 2B), it looks like 1000-fold vs 30-fold induction. I agree that the rankings look similar, but I would hardly call that "similar activity". I get that EF1alpha is a stronger promoter, but the wording should be tweaked a bit here.
2. How was a single TF chosen to plot the expression levels shown in Figure 1C and Figure 1D? any of those ETS motifs could correspond to several different Ets-family proteins, for example (same with NFAT/Fox/IRF/others). Something like a violin plot, which would show all possible TFs that recognize each motif, might be more informative.
3. "As the library contained each single-base substitution to the p53 family consensus sequence, we

were able to generate an activity position weight matrix (PWM) for the consensus. The activity PWM was highly similar to the SELEX derived motif for the p53 family” – can a similar exercise be performed for some other TFs? P53 has a very information-rich motif (and very high activity), so I would think it might be a bit of an outlier in terms of TF behavior in these assays.
4. Only five de novo motifs are shown in Extended Data Fig. 5a. One of these looks like a likely ‘composite element’. This is presented as evidence that “the backbone of the transcriptional system is based on individual TFs acting together without strict spacing preferences or grammar”. I would need to see at least the top 40 de novo motifs in the random enhancer library, along with their p-values, predicted frequencies of occurrence, and enrichment scores to really buy this argument. This would also help bolster the results of the regression analysis.
5. What happens if you use the de novo motifs in the regression analysis? I like seeing the results that use known motifs (since it is less circular), but it would be interesting to see how that compares in overall performance, and also to see if any composite motifs have strong weights.
6. Ext Data Fig 7i – how can the “random Starr-seq CNN” achieve such a strong precision/recall curve? I cant quite tell from the legend what it really is plotting (“random STARR-seq enhancer sequences”). Are these regions that have enhancer activity that were randomly selected? Are they random selections of tested enhancer sequences, regardless of Starr-seq activity? Were they from the “random DNA” library, and had enhancer activity? Are they random genomic regions? (If it is the latter, then something must be wrong with the calibration, given how strong these precision/recall curves are). Please clarify, and perhaps further emphasize what you are plotting by adding e.g. a dotted line at the random expectation level (i.e., some sort of clear baseline).
7. Fig 3b – it seems very odd that bZIP motifs have stronger activity at promoters than enhancers, given that AP-1 (a major class) is famous for being a marker of enhancers. Please clarify what type of bZIP motif this is (perhaps it is CEBP instead?)
8. How were the motifs shown in Fig 3d selected? Were these hand picked, or are you showing all motifs with some sort of positional enrichment?
9. Fig 4g – “The score indicates the fraction of predicted TSS positions falling within ±25 bp (the area shaded with green) from the annotated TSS positions in the genome for each model separately.” How many predicted TSS positions are being made by each method? A more selective model that emphasizes specificity over sensitivity would have an advantage in this scoring system. For an extreme example, a trivial method that only makes one very strong prediction could easily achieve a perfect score of 1.
10. How certain are you regarding the conclusions of Fig 5C? I agree with the data as presented. But I am not sure that you have a large enough sequence space, even given the huge size of your library. To me, the data presented indicate that random DNA that can act as an enhancer does not generally “hit the jackpot” and activate a given promoter more than would be expected by an additive model. But this does not preclude the possibility of a small handful of enhancer DNA sequences that can. Given that evolution acts in a highly non-random manner, it still seems quite feasible that there might be many examples in the genome of enhancers with stronger than additive effects on particular promoters. (If Fig 5C is actually using genomics sequences and not random sequences, then my argument does not hold – if so, perhaps this can be clarified in the legend and text).
11. Was ‘broad peak calling’ really used for ATAC-seq, as stated in the methods? ‘Narrow’ is standard for ATAC-seq, and it looks from your browser shots that this would be more appropriate for your data
12. Very little is presented regarding the effect of DNA methylation on the Starr-seq libraries. Some results appear to be buried in Extended data Fig 7, but they are never explicitly mentioned in the main text, even at a high level. Can any conclusions be drawn from these data?
13. These cells are not being stimulated (other than the transfections themselves) – you therefore are likely missing out on a lot of stimulation/context-dependent TFs (e.g., immune system response) –

please add this to the discussion.
14. Ref 19 (de boer et al) is a very similar study to this one (although it is in yeast, not human). Please add a comparison of the overarching conclusions of these two studies to the discussion – they represent very nice "extreme ends" of our current regulatory logic knowledge in eukaryotes.
15. Previous studies have demonstrated strong motif spacing effects, and motif dependency, on transcriptional output (e.g., early MPRA-type work from Segal lab). It is quite possible that, even with your enormous libraries, you are unable to detect these dependencies, especially if it is only true for a subset of TF pairs/sets and spacings. Please mention this as a caveat to your overall conclusions.
16. In the "Transcriptional enhancers can readily evolve de novo from random sequences" section, a convolutional neural network (CNN)-based classifier similar to DeepBind was fit to the data to "identify all sequence features". While a CNN based classifier is a powerful tool to understand and extract useful sequence patterns, it does not necessarily extract all sequence features, which are highly dependent on the network architecture design, and its capacity. According to the CNN configurations presented in S. Table 9, the filter/kernel numbers in the first convolutional module was limited to 256, while the kernel size was limited to 5. At best, this would approximate ~¼ of the sequence space of the same size without any probabilistic modeling. This issue needs to either be addressed in the design of the CNN, or mentioned as a caveat to any interpretations of the data.
17. In the same section, the authors discuss that analysis of the trained CNN classifier revealed that it has learned motif features similar to those identified by the logistic regression. It is not clear how the authors reached such a conclusion. The authors discussed interpretation of convolution neural network classifiers in the methods section and presented DeepLift-based visualization coupled with the mutual information (MI) analysis pipeline. But it would be more informative to directly see the aggregated sequence patterns the network has really learned by further pursing the model interpretation analysis, e.g., by extending the work with TF-Modisco analysis following the DeepLift output.

Minor text/figure issues:
1. A more through introduction about the library design would be useful in the main text, especially for the TF motif library.
2. As written, it was unclear to me at first that the ATI experiments were performed specifically for this study (I initially thought you were comparing to the published mouse data)
3. Ext Data Fig 6 would be cleaner if it restricted to only showing TFs (looks to me like it is showing all genes)
4. The Venn Diagrams shown in Fig 2 are misleading (they do not include the entire "universe" – the versions shown in extended data fig 7g are more true to the actual data
5. Fig 5c – (important typo) – in the figure legend, based on the text and the simple equations shown, I think you swapped the "enhancer from input" and "promoter from input" labels
6. Fig 5D – why are CREB, ETS, and NRF shown downstream of the TSS in the model? Based on Fig 4d, they should be upstream along with the TATA box
7. A public genome browser session containing your data would be very useful for the community.
8. The "Data and code availability" section currently has placeholders for code and data access – please make sure they are available upon publication

Reviewer #3:
Remarks to the Author:
This manuscript describes the results from a large Starr-seq experiment performed across multiple cell types. The experiment is designed to test combinations of TF binding sites, genomic DNA sequences,

and randomly generated sequences for regulatory activity. The primary claims of the resulting analyses are 1) that enhancers come in four distinct functional classes, 2) that only a small number of TFs are specific for each cell type, 3) that interactions between TFs do not play a large role in setting enhancer activity, and 4) that there are few if any specific interactions between enhancers and promoters.

It would be very exciting and impactful if these claims could be supported. However, as written, the manuscript does not support these claims. The text was disorganized and I could not align what was claimed in the manuscript with what was being shown in the figures. I kept thinking that there were interesting results in this paper, but I was unable to understand the rationale for the design of the experiments, what was being shown in the figures, or how the results lead to the interpretations. My only comment about the manuscript is to suggest that the authors make a much more determined effort at a clear and methodical presentation of their study.

Reviewer #4:
None

Author Rebuttal to Initial comments

Response to reviewers of Sahu et al.

General response to reviewers and editorial comments

In general, the referees have a positive response to our manuscript, stating that it is "*a well designed and well executed study"* (#2), as well as *"a great set of experiments and a very exciting resource"* (#1). Moreover, the referees state that our manuscript could be "*very exciting and impactful"* (#3) and "*very interesting for the community"* (#2).

The referees raise some points regarding the structure of the manuscript (#1 and #3) and interpretation of the data (#1 and #2), especially regarding the motif grammar. We have addressed all the concerns raised by the referees by re-organizing and re-writing the manuscript as well as by clarifying the text and adding new data analyses as detailed in the point-by-point response to the referees. We acknowledge that large amount of different data sets and analyses make the manuscript complex, and we have paid particular attention to making the revised version clearer for the general audience.

The main changes in the revised version include:

1. We have substantially revised the main text to improve readability and accessibility of the work to the general reader
2. We have included a new Supplementary Note with more detailed description of the rationale of the experiments and the methods used as suggested by Reviewer #1
3. We have re-structured the figure panels to make the message of the manuscript clearer
4. We have included more detailed analysis of the motif spacing and interactions to address the questions regarding motif grammar
5. We have performed new analyses to characterize in detail the motifs enriched in the STARR-seq experiments and the motifs that were learned by the convolutional neural networks
6. We have added new experimental data to address the extent of cellular alarm responses in our experimental system

We thank the reviewers for their constructive comments and feel that the revisions they suggested have significantly strengthened the manuscript. In the following pages is our point-by-point response to all specific criticisms of the reviewers. The reviewers' comments are in *italic*, and our response to them is in roman, with the changes made to the manuscript indicated in **bold**. Affected page and line(s) are also indicated.

Reviewers' Comments:

Reviewer #1:
Remarks to the Author:
In their manuscript "Sequence determinants of human gene regulatory elements" Biswajyoti Sahu et al. performed a number of very interesting reporter assays (STARR-Seq) and in vitro binding (ATI) experiments, as well as created a number of supporting RNA-seq, ATAC-seq and ChIP-seq data sets. The result is a very interesting (but also very complex) read out of regulatory elements in (mostly) two cancer cell-lines (HepG2 and GP5d). There are multiple different STARR-Seq assays in this study and the authors branch out into studying effects of methylation, the interaction of regulatory sequences ("promoter"-"enhancer" interactions) as well as the effects of a specific TF knockout (TP53). This all is supplemented by an in-depth modeling analysis using regression, gapped-kmer or CNN models.

We thank the reviewer for the interest and positive feedback to our study.

Trying to put all that into a single study comes at the cost of oversimplifying a story in the main text (without a comprehensive discussion of individual limitations) while providing a very long method description that is too complex to follow for anyone who is not acquainted with the study. My first suggestion would be to "divide and conquer" and to publish this work as a set of two to three articles in the "same issue" of a journal. I believe that we are looking at some very interesting work here, which is really not well represented the way it is currently written up. If splitting up is no option, I would highly recommend to write a structured supplemental note where the individual experiments are motivated and outlined, and most importantly their limitations discussed.

We agree that the large amount of different data sets and analyses make the manuscript complex, and we thank the reviewer for pointing out these important concerns. As suggested by the reviewer, **we have now included a Supplementary Note structured according to the main figures of the manuscript that describes the individual experiments with discussion about their limitations. In addition, the manuscript text, sub-headings, figure headings and the organization of the figure panels has been revised in order to make the manuscript clearer.**

Another high-level criticism is the availability of models and raw as well as processed data. While the authors announce that models and raw data will be released, this is something that should be done before submitting revisions of the manuscript. The reviewers need to see that all data was deposited with the appropriate meta-information.
Specifically, all data should be deposited through NCBI GEO to allow that also processed data is released. Similarly, data/methods cannot be referenced with a manuscript "in preparation" (L733). Either this manuscript is available as pre-print or the information is provided as "personal communication", in which case a sufficient description has to be provided to the reader/reviewers.

We agree that it is important to release the data, and we apologize for not having them available during the initial submission. As suggested by the reviewer, **we have now submitted the raw and processed data to GEO (GSE180158) and machine learning models to Zenodo (10.5281/zenodo.5101420). The data is currently private and will be made public upon publication of the manuscript. However, read-only access token to the GEO data will be available to the Reviewers through the Editor. Furthermore, a genome browser session with the analyzed data will be made available upon publication** as suggested by Reviewer #2. **The accession numbers have been updated to the revised manuscript (page 67, lines 1634-1639; page 69, lines 1682-1686). We have also updated the**

reference for TT-seq in the Methods (page 32, line 786), so the text no longer states "manuscript in preparation".

As briefly mentioned above, the current version of the main manuscript appears to me more of a big picture story telling; while many important details are only available in the methods and a discussion of experimental limitations is missing. In this context it seems most important that even if trying to simplify for a broad readership, a correct picture is conveyed. For me issues arise for the following topics:

(1) It is essential to mention the applied experimental methods early on (i.e. in the abstract) and the authors should refrain from comparing to an "aggregated length" of tested sequences throughout the manuscript. The authors tested millions of short sequences, they never tested long sequences and a concatenated length is of no relevance here as these sequences did not act in that aggregate form.

We agree that it is important to describe the methods early in the manuscript. **To give more emphasis to the methods, we have modified Figure 1 to provide more details about different reporter libraries, experimental workflow, and data analysis types that were used in this manuscript. Moreover, more experimental details are given in the beginning of the Results section (page 3, lines 60-64) and throughout the Supplementary Note. As suggested by the Reviewer, we have modified abstract to mention the main methods (page 1, line 23), but we feel that the word limit for the abstract makes it difficult to cover the methods in more detail there.**

We thank the reviewer for pointing out the potential confusion in using the term "aggregate length" of the tested sequences. **We have revised these statements in the abstract (page 1, line 22) and the main text (page 13, line 322; page 18, line 440) to better reflect the nature of the experiments** that indeed test collections of short sequences as pointed out by the reviewer but represent a sequence space that is ~ 100x larger than that of the human genome.

(2) The authors talk about "evolved" sequences, but what they mean is that they tested random sequences and that a proportion of those showed activity. This has nothing to do with molecular evolution. In some contexts, it might be sufficient to talk about motifs/sequences that are enriched/overrepresented among those activating expression.

We agree that molecular evolution typically refers to changes in sequence in response to selection pressure and apologize for using this imprecise term. **We have now changed or removed the term "evolved sequences" throughout the revised manuscript and replaced it with more accurate ones (page 5, line 113; page 6, line 132; page 7, line 175; page 10, lines 241, 250; page 11, line 260; page 13, lines 307, 315; page 15, line 364; page 17, line 407, page 92, line 2122; page 95, line 2151; page 96, lines 2178, 2185).**

(3a) The authors are doing an unusual normalization of STARR-Seq data, which is typically used as signal over background measure. Due to the high library complexity, sequencing the genomic background is uneconomical (L846ff) and the authors try to normalize differently (e.g. by comparing motif enrichments vs a random sample of the input). This however cannot correct for artifacts in the STARR-Seq data or for more subtle effects like GC content. The authors need to discuss these technical aspects of their data (and not just in the methods).

We thank the reviewer for the important comment and apologize if the details of the analysis of genomic STARR-seq were not clear in the original version of the manuscript. **We have now clarified on page 8, lines 180-182 in the Results section of the revised manuscript that for genomic STARR-seq we**

have used standard peak caller MACS2 (as used previously for example in Peng et al., 2020, PMID: 3291229) **and further discussed the analysis of genomic STARR-seq in Supplementary Note (page 6, lines 267-278). For the random STARR-seq, we analyzed enrichment of sequence features, and used *de novo* motif mining and convolutional neural network (CNN)-based methods. We agree that motif mining approach (but not CNN) could miss subtle effects like differences in mononucleotide or dinucleotide content, but we have now re-analyzed the data and confirm that these effects seem minimal since the dinucleotide frequencies follow those expected from the mononucleotide frequencies and the GC-contents of the motifs do not correlate with the motif activities. We have added these analyses to the revised manuscript (pages 44, lines 1084-1086; page 46, lines 1128-1133) and further discussion about the analysis of ultra-complex libraries to Supplementary Note (page 6, line 29–page 7, line 308)**. We have also clarified that we have corrected technical artifacts by removing sequences containing parts of the primer sequences or long stretches of G's that originate from the Illumina NovaSeq sequencing chemistry. **We now explain this in the revised manuscript (page 44, lines 1083-1084).**

(3b) STARR-Seq (like also MPRA/CRE-seq approaches) seem inherently biased (e.g. due to a minimal promoter) towards activating rather than repressing effects. How does this impact your study and the drawn conclusions?

We agree that MPRA approaches primarily measure transcriptional activation. We do, however, detect some repressive motifs such OVOL1 and CUX but with weaker sensitivity, and OVOL1 was also identified as a negative feature by our logistic regression model. **These findings are now highlighted in updated Figures 2d and 5b and described in the revised manuscript (page 6, lines 137-139 and page 11, 267-269)**. Most regions of the genome are not actively repressed by sequence-specific DNA binding proteins, but simply fail to activate transcription due to lack of transcriptional activators. Thus, we feel that meaningful conclusions about the active TF motifs within enhancers can be drawn from our results. However, designing experiments for measuring repressive effects would certainly be an interesting direction for future studies. **Discussion about measuring the repressive effects is also added to the Supplementary Note (page 7, lines 309-314).**

(3c) STARR-Seq assays also cause a strong cellular "immune" response and you seem to be dealing with a rather high number of plasmids per cell (L876), how does that impact your results? What about plasmid silencing?

We agree that this is a genuine concern based on the previous reports in the literature (such as Muerdter et al., 2018; PMID: 29256496) and since we also observe strong enhancer activity from the p53 and IRFs. **To address the extent of the cellular alarms in our experimental system, we performed new control experiments in which the cells were transfected with the genomic reporter plasmid library or treated with 5-fluorouracil to induce p53. Cellular responses were analyzed by RNA-seq for gene expression, ATAC-seq for chromatin accessibility, and ChIP-seq for p53 and IRF3 binding as well as for active histone mark H3K27ac.** Importantly, we found that plasmid transfection itself caused very minor changes in the cellular transcriptome or p53 and IRF binding, and gene set enrichment analysis showed that p53 target genes were strongly enriched in 5-fluorouracil-treated cells, but not in cells transfected with the STARR-seq library. These results indicate that in our system, the alarm response is very mild and the enrichment of p53 and IRF motifs in the STARR-seq experiments is not an artefact from the experimental system but a measurement of their actual enhancer activity in the cells analyzed. **These findings are shown in new Extended Data Fig. 1c-f and referred to in the revised manuscript (page 3, lines 83-85), described in the Methods (page 28, line 695-page 29 line 699;**

page 29 lines 702, 714 onwards; page 31, lines 750-752), and further discussed in the Supplementary Note (page 2, lines 53-71).

(Very incomplete list of) minor/other comments:

- L67: provide length/context of TF binding motif MPRA

The length of the sequence context has been added as suggested by the reviewer (page 3, lines 63-64 of the revised manuscript).

- L110: active TF identification (ATI) need more of an introduction

Main text has been revised to clarify the part describing ATI results (page 4, lines 92, 95-97), and more detailed introduction to the ATI method has been included in the new Supplementary Note (page 2, lines 83-97).

- L220: discuss how this relates to "primed" enhancers and whether these are active enhancers in other cell-types that only become active because they are moved into an active chromatin context.

We agree that it is an interesting question whether the enhancer classes described in this manuscript have features of the previously described primed enhancers. **We addressed this question by overlapping the peaks representing different enhancer classes with the H3K4me1 histone mark that is generally considered to mark the primed enhancers. This analysis revealed that cryptic enhancers do not show overlap with the H3K4me1 mark, but around one fourth of the closed enhancers are enriched for H3K4me1. These results are now shown in new Extended Data Fig. 9d.** However, since primed enhancers are considered to be located in open chromatin (based on e.g. Heinz et al., 2015, PMID: 25650801), closed enhancers do not fully represent the features of “primed” enhancers. **These aspects are now discussed in the Results (page 9, lines 224-227) and Supplementary Note (page 5, line 248-page 6, line 266).**

- L347: "Molecular evolution approach" -- Is there a prior definition of what this entails? I am having issues with the term evolution for this study.

We thank the reviewer for the comment. **We have now modified the statement and removed the term “molecular evolution” (page 15, line 358).**

- L401ff: The statement about TFs as basic unit makes sense and is probably not challenged. The argument whether "regulatory elements are not atomic units of gene expression" seems to be semantic one, especially as the authors argue for an additive effect of multiple TFs with some activation limit. Considering that the authors are testing these TFs in (very) close proximity (due to technical limitations), it seems an unsupported statement that there would be no length-restricted local neighborhood within the TFs act additively on expression regulation and that these should not be called regulatory elements.

We agree that the statement was too strong, and we did not intend to imply that there are no gene regulatory elements. **We have now removed this statement and re-written the sentence to clarify this (page 17, lines 419-421).**

- L522ff: Details about amount of DNA and cycles missing

Missing details have been provided in the revised manuscript (page 23, lines 546-547).

- L539ff: Amount of material, estimate of oligo complexity

We thank the reviewer for an important comment and apologize for the missing detail. **The amount of material and estimate of oligo complexity (~3 x 10^{12} molecules) have been added to the revised manuscript (page 23, lines 567-568).**

- L558ff: It remains unclear to me how the reporter gene is inserted in between "promoter" and "enhancer" random oligo regions, the size of 555 bp seems too short to include it. Please clarify.

We thank the reviewer for the comment and apologize for the confusing statement. The promoter-enhancer library was cloned into a modified plasmid backbone that harbors a small *Drosophila* ORF instead of a reporter gene, as described in Methods (page 20, lines 484-487). **To indicate this also in the context of library cloning, we have clarified the statement on page 24, lines 584-585.**

- L647ff: Template switching seems to be the required method for reading the "promoter"-"enhancer" STARR-seq library. This needs to be clearer. Also Fig4a (L1890) needs a complete description of the process. You should also bring the corresponding analysis methods closer in text (i.e. L995ff and L1010ff).

We thank the reviewer for the comment and apologize if the usage of the template switch approach was not clear in the original version of the manuscript. **We have revised the figure legend for current Fig. 6a (page 95, lines 2149-2150) to indicate, for which figure panels the template switch data was used.** Specifically, readout from the template switch experiment was used for the analyses in which the information about the TSS positioning within the random promoter was required (i.e. in Figs 6b-d). However, template switch method is not required for other types of analyses of the promoter-enhancer STARR-seq data, such as the motif enrichment and interaction analyses shown in Figs 5 and 7. **Datasets that were used for generating different figures in the manuscript are detailed in Supplementary Table 7, and reference for that is now also provided in the legend for Fig. 6a (page 95, line 2150). In the revised legend for Fig. 6a, we have also provided more details of the experimental workflow of the template switch method, as suggested by the reviewer (page 94, line 2144-page 95, line 2149). We have also clarified in the Methods the relationship between template switch library preparation and data analyses (page 28, lines 688-689; page 42, lines 1030-1031; page 48, lines 1183-1185).**

- L729: Description of bioinformatics analysis of data as well as of enriched motifs/known factors seems to be missing.

We thank the reviewer for pointing out this omission. **We have now modified the Methods (page 32, lines 781-783) to describe that the data preprocessing was done as described earlier (Wei et al, 2018; 29786094) and that the analysis of enriched motifs was done as for the other data sets. We have also mentioned in the Supplementary Note (page 2, lines 94-97) that the two most strongly enriched motifs in GP5d cells from the ATI assay, NFI and NRF1, correspond to TFs that bind strongly in many tissues (Wei et al, 2018; 29786094).**

- L830ff: In a previous analysis you used the LFC package, why are you not using it here?

We thank the reviewer for pointing out this inconsistency. **We have now re-analyzed this data using the LFC package and updated this information to Methods (page 36, line 882). We have also otherwise revised and extended the activity motif position weight matrix (PWM) analysis and generated activity PWMs for other TFs as well as described in response to point 3 by Reviewer #2. The updated activity PWM for p53 is now shown in Fig. 1d and the activity PWMs for other TFs in new Extended Data Fig. 2d. The results are described in the revised manuscript on page 4, lines 84-87.**

- L1000: "Hamming distance of less than 3". You are probably trying to handle synthesis and sequencing errors here. Please provide more insight into why you are doing things and why you are picking these thresholds.

We thank the reviewer for the comment and admit that the rationale for choosing the threshold was not properly explained. The thresholds were chosen in such a way that it would be very unlikely to have multiple sequencing errors in one sequencing read to avoid a situation in which PCR duplicates with sequencing errors would mistakenly be considered as unique reads. **We have now described the rationale in the Methods (page 37, lines 903-905) in the context of preprocessing of random enhancer-library sequences as it is the first place where the issue comes up, and indicated this also in the section for Preprocessing of the random promoter-enhancer pairs (page 44, lines 1077-1078).**

- L1070: You are using Ensembl (L) and EPD (L1031) for TSS annotation across different analyses, you therefore need to specify the source in each case.

We apologize that we have not been clear enough when describing the TSS analysis**. We have now updated the Methods section to indicate which TSS annotation or data set was used in each analysis (page 42, line 1030; page 48, lines 1183-1185; page 49, lines 1202-1203).** Specifically, the section “Motif match positioning relative to TSS and STARR-seq vector” refers to the TSS derived from the random STARR-seq promoter library and thus the **human TSS annotation was not used there (clarified on page 48, lines 1183-1185**), whereas in the cases where human TSS have been analyzed, the specific human TSS annotation has been used as indicated in respective sections (page 41, line 1012; page 42, line 1020; page 45, line 1114; page 65, line 1603; page 66, lines 1609, 1631).

- L1098: Why adding a pseudo count of 10?

We thank the reviewer for pointing out the missing description about adding the pseudo count. **We have now clarified this in the Methods (page 50, lines 1216-1223).**

- L1150ff: Regularization does not protect from issues with correlated features. It might slightly help the interpretation, but it does not solve the actual issue.

We thank the reviewer for the comment. **We have now clarified the text regarding the correlated features and L1-regularization in the Methods (page 52, lines 1273-1281)**. In fact, LASSO regression (regression with L1-regularization) has been shown to work well with correlated features given that the regularization strength is adjusted properly (e.g. Hebiri & Lederer, 2012, arXiv:1204.1605). With a properly adjusted regularization strength, L1 norm will select only one out of a set of highly correlated features. This is in contrast to L2 norm which will favor weighting collinear features equally. Thus, we feel that L1-regularization is suitable for the analysis in this manuscript.

- L1843: Not STARR-seq on its own, but the combination of readouts reveals the different types of regulatory elements. Especially as STARR-Seq does not show signal for some otherwise defined elements.

We thank the reviewer for pointing out the imprecise wording**. We have now modified the figure title (page 91, line 2096) and the respective sub-heading in the Results section (page 7, line 175) to accurately reflect the analysis that was performed.**

Textual errors:
- L187: [the] GP5d genome
- L702: correct sentence around "lysed"
- L867: which -> with
- L1817: delete "relatively"

We thank the reviewer for the detailed comments regarding the manuscript text. **We have now corrected these points in the revised version of the manuscript (page 7, line 178; page 31, line 754; page 38; line 931; page 85, line 2022).**

Overall, this is a great set of experiments and a very exciting resource. I hope the authors can make all this a bit better accessible to the reader. Even as a very interested reader, I could only get through a very thin layer of this work.

We thank the reviewer for very detailed and constructive comments regarding our manuscript. We have carefully revised the manuscript to make it clearer and paid special attention to the points raised by the reviewers. We hope that the revised manuscript is more accessible to the readers.

Reviewer #2:
Remarks to the Author:
The authors present an interesting study examining the transcriptional activity of 400 billion DNA bases in three cellular contexts using STARR-seq. Collectively, their study examines ~100 times the sequence capacity of the human genome, and therefore serves as a strong platform for a large-scale unbiased view of the sequence determinants of human gene regulatory element activity.

Several key observations are presented based on the data:
1. There are three classes of enhancers, with different mechanisms and motif content
2. Transcription factors (TFs) generally have (weak) additive effects
3. Only a few TFs are strongly active in a cell type, with the strongest differences between cells representing known TFs that are important for specification and/or function of the specific lineages
4. Only a subset of TFs have TSS positional dependency
5. There is no evidence for 'beyond additive' interactions between promoters and enhancers
Overall this is a well designed and well executed study. I expect it will be very interesting for the community and will spark a good amount of discussion and debate. That said, I have several comments, most of which involve data interpretation.

We thank the reviewer for the positive comments and the interest in our study.

Analysis/presentation/interpretation issues:
1. "The most active motifs displayed similar activities when placed in different sequence contexts, and between experiments using two different basal promoters, δ1-crystallin and CpG-free EF1α promoters" – not sure I totally agree with that 2nd part. For the top motifs (p53, IRF HT2 in Ext Data Fig 2B), it looks like 1000-fold vs 30-fold induction. I agree that the rankings look similar, but I would hardly call that "similar activity". I get that EF1alpha is a stronger promoter, but the wording should be tweaked a bit here.

We thank the reviewer for pointing out the imprecise wording. **We have revised the statement to indicate that the motif activities are consistent between the backbones (page 3, lines 78-79).**

2. How was a single TF chosen to plot the expression levels shown in Figure 1C and Figure 1D? any of those ETS motifs could correspond to several different Ets-family proteins, for example (same with NFAT/Fox/IRF/others). Something like a violin plot, which would show all possible TFs that recognize each motif, might be more informative.

We acknowledge that assigning the enriched motif activity to a specific TF is not always possible due to the facts that many TFs bind to highly similar motifs and the binding specificities for all individual TFs are not yet known. **We have therefore updated old Fig. 1c to show expression levels of all TFs in the same structural family and moved it as new Extended Data Fig. 2a and added discussion about these limitations to the new Supplementary Note (page 1, line 33 onwards). In addition, we have a new main figure panel (Fig. 3a), which shows the difference in mRNA levels for all TFs in two cell lines that in part can explain the observed differences in motif activity.**

3. "As the library contained each single-base substitution to the p53 family consensus sequence, we were able to generate an activity position weight matrix (PWM) for the consensus. The activity PWM was highly similar to the SELEX derived motif for the p53 family" – can a similar exercise be performed for some other TFs? P53 has a very information-rich motif (and very high activity), so I would think it might be a bit of an outlier in terms of TF behavior in these assays.

We agree that it is an interesting question whether PWMs for other TFs besides p53 can be derived from the measured motif activities. To address this, **we extended the analysis of motif activities with one nucleotide substitutions to other TFs as well and were able to generate activity-based PWMs for several other TFs. To highlight these results, the activity PWM for p53 is now shown in the revised main figure (Fig. 1d) and the activity PWMs for other TFs in new Extended Data Fig. 2d. The results are described in the revised manuscript on page 4, lines 84-89 and further discussed in the Supplementary Note (page 1, lines 24-32).**

4. Only five de novo motifs are shown in Extended Data Fig. 5a. One of these looks like a likely 'composite element'. This is presented as evidence that "the backbone of the transcriptional system is based on individual TFs acting together without strict spacing preferences or grammar". I would need to see at least the top 40 de novo motifs in the random enhancer library, along with their p-values, predicted frequencies of occurrence, and enrichment scores to really buy this argument. This would also help bolster the results of the regression analysis.

We thank the reviewer for the important comment regarding the motif spacing. **We agree that the statement pointed out by the reviewer was too strict and thus we have removed it from the revised manuscript. To address the question regarding motif spacing and grammar, we have now performed more thorough *de novo* motif mining of the random enhancer STARR-seq data. The new results are shown as Extended Data Fig.6a and described on page 5, lines 121-122 of the revised manuscript. We have also performed new motif matching analyses (shown in new Extended Data Figs. 5c, d, page 9, lines 213-216; page 10, lines 234-236) as well as further analyses to address the motif spacing as described in the response to point 15.**

5. What happens if you use the de novo motifs in the regression analysis? I like seeing the results that use known motifs (since it is less circular), but it would be interesting to see how that compares in overall performance, and also to see if any composite motifs have strong weights.

We thank the reviewer for the comment and agree that it is an interesting question whether the *de novo* motifs can be used in the regression analysis. **To address this, we have performed logistic regression analysis of the random enhancer STARR-seq experiment using the *de novo* motifs. The classification performance of the *de novo* motif-based logistic regression classifier was similar to the HT-SELEX motif-based logistic regression (*de novo* logistic regression and the HT-SELEX motif-based logistic regression both obtained AUprc≈0.55). The weights of the most important features in the *de novo* motif-based logistic regression are shown in new Supplementary Figure 6b and described in the Supplementary Note (page 3, lines 131-146).**

6. Ext Data Fig 7i – how can the "random Starr-seq CNN" achieve such a strong precision/recall curve? I cant quite tell from the legend what it really is plotting ("random STARR-seq enhancer sequences"). Are these regions that have enhancer activity that were randomly selected? Are they random selections of tested enhancer sequences, regardless of Starr-seq activity? Were they from the "random DNA" library, and had enhancer activity? Are they random genomic regions? (If it is the latter, then something must be wrong with the calibration, given how strong these precision/recall curves are). Please clarify, and perhaps further emphasize what you are plotting by adding e.g. a dotted line at the random expectation level (i.e., some sort of clear baseline).

We thank the reviewer for the comment and apologize for the unclear figure legend. The random STARR CNN was trained using the data from the GP5d random enhancer STARR-seq experiment. We have used balanced test sets (equal number of samples from the classes) in all binary classification tasks for

easier interpretability, meaning that area under precision-recall curve is 0.5 if class labels are assigned at random by a predictor. **We have now modified the figure legend for the current Extended Data Fig. 9e to clarify these points.**

7. Fig 3b – it seems very odd that bZIP motifs have stronger activity at promoters than enhancers, given that AP-1 (a major class) is famous for being a marker of enhancers. Please clarify what type of bZIP motif this is (perhaps it is CEBP instead?)

We thank the reviewer for pointing out the naming of the bZIP motif. **Specifically, the motif in the figure is JUN that is now indicated in the current Fig. 5b.**

8. How were the motifs shown in Fig 3d selected? Were these hand picked, or are you showing all motifs with some sort of positional enrichment?

We thank the reviewer for pointing out the missing details of the selection of the motifs shown in the figure. The motifs that are shown in the heatmap are the classical TSS-associated motifs (Initiator, TATA), the most highly enriched motifs at the promoters compared to enhancers, and generally highly enriched motifs (p53 family, YY, and CREB:MAF). **This is now clarified in the figure legend for current Fig. 6d in the revised manuscript (page 95, lines 2160-2163).** Interestingly, a very recent report has described a novel BANP motif that is enriched at gene promoters (Grand et al, 2021; PMID: 34234345). To analyze whether the BANP motif can also be detected from the *de novo* promoters enriched from the random sequences, we re-ran the analysis with BANP included. Interestingly, we observed a strong enrichment for the BANP motif at the *de novo* promoters upstream of the TSS. **These new results are shown in updated Fig. 5b, c and Fig. 6d and described on page 11, lines 264-265 and page 12, lines 301-302 of the revised manuscript.**

9. Fig 4g – "The score indicates the fraction of predicted TSS positions falling within ±25 bp (the area shaded with green) from the annotated TSS positions in the genome for each model separately." How many predicted TSS positions are being made by each method? A more selective model that emphasizes specificity over sensitivity would have an advantage in this scoring system. For an extreme example, a trivial method that only makes one very strong prediction could easily achieve a perfect score of 1.

We thank the reviewer for the comment and apologize for the confusing figure legend. In fact, in the analysis we made one prediction per TSS from each model, and the accuracy of these predictions was compared. **This aspect is now clarified in the figure legend (currently Fig. 6g) on page 96, lines 2180-2182.**

10. How certain are you regarding the conclusions of Fig 5C? I agree with the data as presented. But I am not sure that you have a large enough sequence space, even given the huge size of your library. To me, the data presented indicate that random DNA that can act as an enhancer does not generally "hit the jackpot" and activate a given promoter more than would be expected by an additive model. But this does not preclude the possibility of a small handful of enhancer DNA sequences that can. Given that evolution acts in a highly non-random manner, it still seems quite feasible that there might be many examples in the genome of enhancers with stronger than additive effects on particular promoters. (If Fig 5C is actually using genomics sequences and not random sequences, then my argument does not hold – if so, perhaps this can be clarified in the legend and text).

We thank the reviewer for bringing up this important aspect and we agree that genomic enhancers can be stronger than the random enhancers and have specific promoter-enhancer interactions. **To address this,**

we have now modified the Discussion on page 18, lines 433-437 and added examples of such cases, such as the multi-chromosome structures that control the expression of the repertoire of olfactory receptor genes and the complex regulatory landscape of the HOX genes (refs Spitz, Nature 2019 and de Laat & Duboule, 2013, PMID: 24153303)**.** These highly evolved genomic elements involve specificity that we did not observe in our data, suggesting that they have properties of the chromatin-dependent enhancers that STARR-seq cannot detect.

11. Was 'broad peak calling' really used for ATAC-seq, as stated in the methods? 'Narrow' is standard for ATAC-seq, and it looks from your browser shots that this would be more appropriate for your data

We thank the reviewer for the comment. Our ATAC-seq pipeline indeed uses broad peak calling, but in the genome browser snapshots, traces are shown from the BAM coverage file. **This is now clarified in the Methods (page 31, line 762-763) as well as respective figure legend (page 91, line 2112-2113) in the revised version of the manuscript.**

12. Very little is presented regarding the effect of DNA methylation on the Starr-seq libraries. Some results appear to be buried in Extended data Fig 7, but they are never explicitly mentioned in the main text, even at a high level. Can any conclusions be drawn from these data?

We agree that the results from the experiments using methylated genomic library were described in a superficial manner**. In the revised version of the manuscript, we have given more emphasis to these results by including three figure panels in the new Fig. 3 and by describing them on page 8, lines 186-187 and page 9, lines 204-207 and in the Supplementary Note (page 4, line 191-page 5, line 216).** Briefly, we show that cryptic enhancers are generally not silenced by DNA methylation, and that motif enrichment from the STARR-seq peaks called from the experiments using methylated and non-methylated libraries agree with the previously described methyl-plus and methyl-minus TF motifs.

13. These cells are not being stimulated (other than the transfections themselves) – you therefore are likely missing out on a lot of stimulation/context-dependent TFs (e.g., immune system response) – please add this to the discussion.

We agree that the cells in our experimental system are un-stimulated. **This is now mentioned in the new Supplementary Note (page 2, lines 73-76).** Furthermore, the plasmid transfection itself does not induce strong cellular alarm signals. We verified this by performing new control experiments where cellular responses to the plasmid transfection were analyzed using RNA-seq, ATAC-seq and ChIP-seq (see also response to Reviewer #1, point 3C). **These aspects are shown in new Extended Data Fig. 1c-f and referred to in the revised manuscript (page 3, lines 83-85), and further discussed in the Supplementary Note (page 2, lines 53-71).**

14. Ref 19 (de boer et al) is a very similar study to this one (although it is in yeast, not human). Please add a comparison of the overarching conclusions of these two studies to the discussion – they represent very nice "extreme ends" of our current regulatory logic knowledge in eukaryotes.

We thank the reviewer for this important suggestion. **We have now added a sentence in the Discussion to compare the yeast and mammalian regulatory systems (page 15, lines 367-369).**

15. Previous studies have demonstrated strong motif spacing effects, and motif dependency, on transcriptional output (e.g., early MPRA-type work from Segal lab). It is quite possible that, even with your

enormous libraries, you are unable to detect these dependencies, especially if it is only true for a subset of TF pairs/sets and spacings. Please mention this as a caveat to your overall conclusions.

We agree that the motif grammar and spacing are important questions. **Our new analysis of spacing between motif matches identified few significantly overrepresented spacing preferences for motif pairs such GRHL–ATF6. These results are now shown as new main Fig. 2c. Based on mutual information analysis, weak overall preference for motifs being relatively close (<50 bp) was also observed, as shown in new Extended Data Fig. 4e, f. We have described these findings in the Results (page 5, lines 196-202) and discussed them in the context of previous work in the Discussion (page 15, lines 363-365, 369-371) and Supplementary Note (page 3, lines 108-118).**

16. In the "Transcriptional enhancers can readily evolve de novo from random sequences" section, a convolutional neural network (CNN)-based classifier similar to DeepBind was fit to the data to "identify all sequence features". While a CNN based classifier is a powerful tool to understand and extract useful sequence patterns, it does not necessarily extract all sequence features, which are highly dependent on the network architecture design, and its capacity. According to the CNN configurations presented in S. Table 9, the filter/kernel numbers in the first convolutional module was limited to 256, while the kernel size was limited to 5. At best, this would approximate ~¼ of the sequence space of the same size without any probabilistic modeling. This issue needs to either be addressed in the design of the CNN, or mentioned as a caveat to any interpretations of the data.

We thank the reviewer for pointing out this important aspect about the CNN architecture, and we agree that the statement regarding the CNN learning all the sequence features was too strong. **We have now revised the sentence and removed the claim that the CNN can learn all sequence features (page 6, line 141-142). Moreover, to acknowledge the limitations of CNN models in extracting sequence features, we have included discussion about the limit for the number of motifs/filters the CNN models can learn in the new Supplementary Note (page 3, lines 131-161).**

17. In the same section, the authors discuss that analysis of the trained CNN classifier revealed that it has learned motif features similar to those identified by the logistic regression. It is not clear how the authors reached such a conclusion. The authors discussed interpretation of convolution neural network classifiers in the methods section and presented DeepLift-based visualization coupled with the mutual information (MI) analysis pipeline. But it would be more informative to directly see the aggregated sequence patterns the network has really learned by further pursing the model interpretation analysis, e.g., by extending the work with TF-Modisco analysis following the DeepLift output.

We thank the reviewer for pointing out that the statement regarding the CNN model learning similar motif features than the logistic regression model was not sufficiently supported by the results shown in the previous version of the manuscript. **To address this in more detail, we have now analyzed the motif patterns learned by the random enhancer CNN model in three different complementary ways, including the TF-MoDISco analysis suggested by the reviewer. The discovered motif patterns are shown in new Extended Data Figs 6c, 7, and 8 and compared both against the *de novo* motifs from the random enhancer STARR-seq experiment and against the most important motif features used by the logistic regression model.** This analysis shows that the CNN has learned similar motifs than the logistic regression model is using, but that there are differences especially in the lower information content regions of the motifs. **These results are described in the revised manuscript (page 6, line 146-page 7, line 155) and further discussion about the new model interpretation analyses has been included to Supplementary Note (page 4, lines 162-189).**

Minor text/figure issues:
1. A more through introduction about the library design would be useful in the main text, especially for the TF motif library.

As suggested also by reviewer #1, **we have included more thorough introduction to the manuscript text describing the STARR-seq experiments and the used libraries (page 3, lines 60-64; Supplementary Note, page 1, lines 14-23), and, in particular, we have modified Fig. 1a to include more details about the reporter libraries and to highlight the different analyses approaches.**

2. As written, it was unclear to me at first that the ATI experiments were performed specifically for this study (I initially thought you were comparing to the published mouse data)

We apologize for not stating it clearly that the ATI experiments from GP5d cells were performed as part of this study. **The text describing the results from the ATI experiments has now been clarified (page 4, lines 92, 95-97), and more thorough introduction to the ATI method has been included in the new Supplementary Note (page 2, lines 83-97).**

3. Ext Data Fig 6 would be cleaner if it restricted to only showing TFs (looks to me like it is showing all genes)

We thank the reviewer for the comment and agree that showing only the TFs makes the message clearer**. We have now updated the figure to only show the TFs as suggested by the reviewer. The updated figure is currently shown as the main Fig. 3a and referred to on the revised manuscript on page 7, line 166.**

4. The Venn Diagrams shown in Fig 2 are misleading (they do not include the entire "universe" – the versions shown in extended data fig 7g are more true to the actual data

We agree that the Euler diagrams shown in the current Fig. 4a do not include all possible overlaps, but we feel that it is still a good way to visualize the main finding of the different regulatory element classes. **We have modified the legend for current Fig. 4a to indicate this more clearly (page 91, lines 2101-2102).** The full comparison is shown in the Extended Data Fig. 9c. and is referred to in the legend for Fig. 4a.

5. Fig 5c – (important typo) – in the figure legend, based on the text and the simple equations shown, I think you swapped the "enhancer from input" and "promoter from input" labels

We agree that the legend for the figure (current **Fig. 7c**) might have been confusing; however, the labels are correct. **To clarify the matter, we have updated the legend to state that in the "enhancer from input" model, the classification is purely based on promoter features and in the "promoter from input" purely on enhancer features (page 98, lines 2209-2210).**

6. Fig 5D – why are CREB, ETS, and NRF shown downstream of the TSS in the model? Based on Fig 4d, they should be upstream along with the TATA box

We thank the reviewer for the comment and agree that the place where the CREB, ETS, and NRF were located was confusing. **We have now modified the current Fig. 7d to indicate these factors upstream of the TSS as suggested by the reviewer.**

7. A public genome browser session containing your data would be very useful for the community.

We agree that a public genome browser session would be useful for the community, and **a genome browser session with the analyzed data will be made available upon publication (mentioned in the manuscript on page 67, lines 1637-1639; page 69, lines 1685-1686).** We apologize for not having it available yet; we tried making a UCSC genome browser session to be included with this revision, but including several different data sets behind one password-protected link was not supported by the system.

8. The "Data and code availability" section currently has placeholders for code and data access – please make sure they are available upon publication

We agree that it is important to release the raw data as well as machine learning models, and we apologize for not having them available during the initial submission. As suggested by the reviewer #1, **we have now submitted the raw data and the processed files to GEO (GSE180158) and the machine learning models are available at Zenodo (10.5281/zenodo.5101420), and the Data availability statement has been updated (page 67, lines 1634-1639; page 69, lines 1682-1686). The data is currently private and will be made public upon publication of the manuscript. However, read-only access token to the GEO data will be available to the Reviewers through the Editor.**

Reviewer #3:
Remarks to the Author:
This manuscript describes the results from a large Starr-seq experiment performed across multiple cell types. The experiment is designed to test combinations of TF binding sites, genomic DNA sequences, and randomly generated sequences for regulatory activity. The primary claims of the resulting analyses are 1) that enhancers come in four distinct functional classes, 2) that only a small number of TFs are specific for each cell type, 3) that interactions between TFs do not play a large role in setting enhancer activity, and 4) that there are few if any specific interactions between enhancers and promoters.

It would be very exciting and impactful if these claims could be supported. However, as written, the manuscript does not support these claims. The text was disorganized and I could not align what was claimed in the manuscript with what was being shown in the figures. I kept thinking that there were interesting results in this paper, but I was unable to understand the rationale for the design of the experiments, what was being shown in the figures, or how the results lead to the interpretations. My only comment about the manuscript is to suggest that the authors make a much more determined effort at a clear and methodical presentation of their study.

We thank the reviewer for the interest in our study. We agree that the amount of different data sets and analyses make the manuscript dense, and we apologize for providing insufficient or superficial information for following the rationale of the experiments and the conclusions drawn from the results. We have done major revisions to the manuscript with particular emphasis on making it clearer and more approachable. Main changes are the following: (1) we have included a structured Supplementary Note that describes the rationale of the experiments and the methods used in every figure as well discusses the limitations of the chosen methods; (2) we have re-organized the figure panels to have seven main figures instead of five to give more emphasis for individual analyses; (3) we have modified **Fig. 1** to have clearer presentation of the libraries as well as experimental and analysis methods used in this study; and (4) we have revised the manuscript text, including the sub-headings and figure titles to reflect the obtained results in each chapter. We hope that the revised version of the manuscript is more approachable.

Reviewer #4:
None

natureresearch

Decision Letter, first revision:

3rd Aug 2021

Dear Jussi and Biswa,

Thank you for submitting your revised manuscript, "Sequence determinants of human gene regulatory elements".

Due to the current lack of access to all the computer code, we are concerned that sending the manuscript back to the reviewers would lead to unnecessary delays and quite possibly an undesirable outcome of the review process.

Please see https://www.nature.com/nature-portfolio/editorial-policies/reporting-standards for more information on our policies regarding access to data and code.

We shall hope to receive your revised version, including separate data and code accessibility statements, as soon as possible.

Please ensure that you have completed the Reporting Summary required for review:
https://www.nature.com/documents/nr-reporting-summary.pdf

Please use the link below to submit a suitably revised manuscript and updated forms(s):

[REDACTED]

Best wishes,

Tiago

Tiago Faial, PhD
Senior Editor
Nature Genetics
https://orcid.org/0000-0003-0864-1200

Decision Letter, second revision:

24th Aug 2021

Dear Jussi,

Your Article, "Sequence determinants of human gene regulatory elements" has now been seen by the 3 original referees. You will see from their comments below that while they find your work improved, some important points are raised. We are interested in the possibility of publishing your study in Nature Genetics, but would like to consider your response to these concerns in the form of a revised manuscript before we make a final decision on publication.

Reviewer #1 thinks that the study has improved but still feels that it is too complex. Apart from some minor points, their main request is that you include more quantitative information throughout the text.
Reviewer #2 is fully satisfied.
Reviewer #3 raises some pertinent conceptual criticisms. Like reviewer #1, they also think that the amount of data/analyses makes the paper difficult to read/interpret. Perhaps more importantly, the reviewer thinks that the broad conclusions are not supported by the data in the sense that the number of sequences analyzed is biased/limited and therefore you cannot make such strong general statements. We think that this point is well taken and warrants a careful textual revision.

We therefore invite you to revise your manuscript taking into account all reviewer comments. Please highlight all changes in the manuscript text file. At this stage we will need you to upload a copy of the manuscript in MS Word .docx or similar editable format.

When revising your manuscript:

*1) Include a "Response to referees" document detailing, point-by-point, how you addressed each referee comment. If no action was taken to address a point, you must provide a compelling argument. This response may be sent back to the referees along with the revised manuscript.

*2) If you have not done so already please begin to revise your manuscript so that it conforms to our Article format instructions, available
here.
Refer also to any guidelines provided in this letter.

*3) Include a revised version of any required Reporting Summary:
https://www.nature.com/documents/nr-reporting-summary.pdf
It will be available to referees (and, potentially, statisticians) to aid in their evaluation if the manuscript goes back for peer review.
A revised checklist is essential for re-review of the paper.

Please be aware of our guidelines on digital image standards.

Please use the link below to submit your revised manuscript and related files:

[REDACTED]

Note: This URL links to your confidential home page and associated information about manuscripts you may have submitted, or that you are reviewing for us. If you wish to forward this email to co-authors, please delete the link to your homepage.

We hope to receive your revised manuscript within eight weeks. If you cannot send it within this time, please let us know.

Please do not hesitate to contact me if you have any questions or would like to discuss these revisions further.

Nature Genetics is committed to improving transparency in authorship. As part of our efforts in this direction, we are now requesting that all authors identified as 'corresponding author' on published papers create and link their Open Researcher and Contributor Identifier (ORCID) with their account on the Manuscript Tracking System (MTS), prior to acceptance. ORCID helps the scientific community achieve unambiguous attribution of all scholarly contributions. You can create and link your ORCID from the home page of the MTS by clicking on 'Modify my Springer Nature account'. For more information please visit please visit www.springernature.com/orcid.

We look forward to seeing the revised manuscript and thank you for the opportunity to review your work.

Sincerely,

Tiago

Tiago Faial, PhD
Senior Editor
Nature Genetics
https://orcid.org/0000-0003-0864-1200

Reviewers' Comments:

Reviewer #1:
Remarks to the Author:
With the revision of their manuscript "Sequence determinants of human gene regulatory elements", Biswajyoti Sahu et al. addressed a large number of comments of three reviewers. As a result, the

main text is much more streamlined and easier to access. The methods have been extended with some missing details and the data has been prepared for public release. Further, a separate supplemental note was added to provide more motivation and a discussion of some limitations for the large number of experiments and analyses performed. This addresses my major criticism of the first round of reviews.

I still believe that the amount of material and complexity of performed analyses is too much for a single paper and that a comprehensive review of such kind of study is simply not possible. While I feel that, I have conquered a little more territory of this work while reading the updated manuscript, I will not claim that I totally got through the presented materials and analyses. As I said before, trying to put all that information into a single study comes at the cost of oversimplifying a story in the main text. It results in a very long method description that is too complex for most readers to follow and moves the discussion of individual limitations to supplementary documents that will also not be read by most readers. While I understand the pressures that underlie the publication of these massive studies, I do not think that we are doing anyone a good service with it.

I have some random notes/comments below. As indicated above, this is largely incomplete due to the amount of materials available here. There is only one point that I would like to generalize from my notes: Where possible, it would be nice to include quantitative results (number, proportions, statistical test) in the text rather than referring the reader to a figure. Examples (again an incomplete list) of that are line 189 (providing the proportion and maybe significance test), l190 (proportion of ATAC peaks predicted), l230 (number of motifs), l263-l265 (providing some numbers rather than qualitative statements like enrichment, preferential, exclusive), or l299 (enrichment values?). Here line numbers are referring to the updated manuscript and not the one with tracked changes.

Minor comments:
- l42: remove ", respectively"? it suggests that there is a clear cut difference in the presence of these marks while I believe it is more of an enrichment.
- l51: "the 1639 TFs" – approximately/at least? We still do not know all TFs and even if a reference provides a number, I would express some uncertainty.
- l61: "minimal promoter to an exon" to "minimal promoter in a reporter gene"?
- l67: "ultra-high complexity[,] reaching billions"
- l96: For me this comes not as naturally as "this suggests" would imply.
- l132: "to determine all sequence features", I would leave out the "all" as it is an absolute statement that is difficult to make.
- l426: "was found[,] is consistent"
- l1394: "external evaluation[,] we trained"
- l1633-1639: repeated later in the document

Reviewer #2:
Remarks to the Author:
The authors have done an excellent job of responding to my comments and criticisms. No further issues remain on my end.

Reviewer #3:
Remarks to the Author:
This is a revision of the manuscript from Sahu et al. describing a large set of MPRA experiments. I appreciate the authors' efforts to improve the clarity and organization of the manuscript. I still think that there is a lot of material here for one paper, and that this is making the paper difficult to understand. However, the larger problem is that the data in the manuscript do not support the unusually broad conclusions.

One claim the manuscript makes is that the study is "comprehensive", which implies that all of regulatory space has been assayed. The text makes a compelling argument that regulatory space is too big to be encompassed by sequences found in the genome, and that therefore, synthetic DNA sequences must be included. However, even though the DNA assayed here is "~100 times larger sequence space than the human genome" (line 22) when one considers "all combinations, orientations and spacings of the 1639 TFs in multiple independent sequence contexts" (lines 51-52) the sequence space assayed in this study still represents only a tiny fraction regulatory space, which undermines the claim of being comprehensive.

Another caveat related to the size of regulatory space is the claim that most TFs work independently and additively, without cooperative interactions. Since only a small fraction of regulatory space is being assayed, most combinations of TF sites, in most orientations and spacings, were not measured. Thus, the study is underpowered to detect interactions between TFs relative to the power to detect the additive contributions of TFs. No strong conclusions should be drawn from the lack of detected interactions: it is the expected result from a study with limited power to detect interactions.

Another example of a strong conclusion drawn from a negative result is on lines 95-97, where the authors invoke the sequential activity of TFs based on the lack of concordance between enhancer activities and in vitro binding data. The possibility that one or both data sets may contain significant errors is not considered.

The claim that only a small number of TFs are active in any cell type cannot be supported from assays in just two cell types, especially when both of the cell types derive from the endoderm.

The claim that there are multiple categories of enhancers is only weakly supported and comes only categorizing active sequences by their epigenetic marks. Any randomly picked group of sequences could likewise be separated by the types of marks they carry, so the mere fact that enhancers have different marks does not prove that they function in qualitatively different ways from each other.

The TFs with the largest response in the experiments are p53 and IRF, two TFs that are part of a known artifact of Starr-seq that has to do with the cellular response to foreign DNA. The authors have done experiments to rule out that the cellular alarm system is not responsible for their measured activities of p53 and IRF, but it remains worrying that this is the largest effect in the data.

Author Rebuttal, second revision:

Response to reviewers of Sahu et al.

General response to reviewers and editorial comments

In general, the referees have responded positively to the revisions we made to our manuscript. They state that "*the authors have done an excellent job of responding to my comments and criticisms*" (#2) and that the manuscript "*addresses my major criticism of the first round of reviews*" (#1). Moreover, the referees mention that they *"appreciate the authors' efforts to improve the clarity and organization of the manuscript"* (#3) and that "*main text is much more streamlined and easier to access*" (#1).

The referees raise some further points regarding missing quantitative details (#1) and interpretation of the data (#3), especially whether the number of sequences analyzed supports the conclusions in the manuscript. We have addressed all the concerns pointed out by the referees by clarifying the text and by adding new analyses of the data, as detailed in the point-by-point response to the referees.

The main changes in the revised version are the following:
1. Addition of exact quantitative information about different analyses throughout the Results-section.
2. New analysis to estimate the power of our assay to detect true motif-motif interactions
3. New analysis to study how large effect p53 has on overall enhancer activity in the cells
4. Re-writing the text to clarify points regarding data interpretation

We thank the reviewers for their constructive comments and feel that the revisions they suggested have further strengthened the manuscript. In the following pages is our point-by-point response to all specific criticisms of the reviewers. The reviewers' comments are in *italic*, and our response to them is in roman, with the changes made to the manuscript indicated in **bold**. Affected page and line(s) are also indicated, the exact line numbers referring to the manuscript document in which the changes have been highlighted.

Reviewers' Comments:

Reviewer #1:
Remarks to the Author:
With the revision of their manuscript "Sequence determinants of human gene regulatory elements", Biswajyoti Sahu et al. addressed a large number of comments of three reviewers. As a result, the main text is much more streamlined and easier to access. The methods have been extended with some missing details and the data has been prepared for public release. Further, a separate supplemental note was added to provide more motivation and a discussion of some limitations for the large number of experiments and analyses performed. This addresses my major criticism of the first round of reviews.

We thank the reviewer for the positive feedback to our revised manuscript.

I still believe that the amount of material and complexity of performed analyses is too much for a single paper and that a comprehensive review of such kind of study is simply not possible. While I feel that, I have conquered a little more territory of this work while reading the updated manuscript, I will not claim that I totally got through the presented materials and analyses. As I said before, trying to put all that information into a single study comes at the cost of oversimplifying a story in the main text. It results in a very long method description that is too complex for most readers to follow and moves the discussion of individual limitations to supplementary documents that will also not be read by most readers. While I understand the pressures that underlie the publication of these massive studies, I do not think that we are doing anyone a good service with it.

I have some random notes/comments below. As indicated above, this is largely incomplete due to the amount of materials available here. There is only one point that I would like to generalize from my notes: Where possible, it would be nice to include quantitative results (number, proportions, statistical test) in the text rather than referring the reader to a figure. Examples (again an incomplete list) of that are line 189 (providing the proportion and maybe significance test), l190 (proportion of ATAC peaks predicted), l230 (number of motifs), l263-l265 (providing some numbers rather than qualitative statements like enrichment, preferential, exclusive), or l299 (enrichment values?). Here line numbers are referring to the updated manuscript and not the one with tracked changes.

We agree that the exact quantitative information was missing from the main text in many instances, and we thank the reviewer for pointing out this shortcoming. **We have now carefully revised the manuscript and added quantitative details throughout the revised manuscript, including the specific examples pointed out by the reviewer. All the affected page and line numbers are the following: page 5, lines 117, 121; page 6, lines 136-137, 147; page 7, lines 164-165; page 8, lines 198-199, 201-202; page 10, lines 242-243; page 11, lines 269-272, 277-280; page 13, lines 314-316; page 14, lines 333-335; page 15, lines 365-366; page 90, lines 2093-2094; page 95, lines 2162-2164).**

Minor comments:

We thank the reviewer for detailed comments on the manuscript text. **We have modified the indicated sentences in the revised manuscript as suggested by the reviewer, and the affected page and line numbers are shown after each comment.**

- l42: remove ", respectively"? it suggests that there is a clear cut difference in the presence of these marks while I believe it is more of an enrichment.

We have modified the sentence to indicate that there is a preferential enrichment and not clear-cut distinction between the marks (page 2, line 41).

- l51: "the 1639 TFs" – approximately/at least? We still do not know all TFs and even if a reference provides a number, I would express some uncertainty.

Modified as suggested (page 2, line 52).

- l61: "minimal promoter to an exon" to "minimal promoter in a reporter gene"?

Sentence clarified (page 3, line 62).

- l67: "ultra-high complexity[,] reaching billions"

Modified as suggested (page 3, line 68).

- l96: For me this comes not as naturally as "this suggests" would imply.

We thank the reviewer for pointing out this confusing statement. **We have now clarified the text on page 4, line 95-97 and removed the confusing statement from the revised manuscript.**

- l132: "to determine all sequence features", I would leave out the "all" as it is an absolute statement that is difficult to make.

Modified as suggested (page 6, line 133).

- l426: "was found[,] is consistent"

Modified as suggested (page 19, line 453).

- l1394: "external evaluation[,] we trained"

Modified as suggested (page 59, line 1436).

- l1633-1639: repeated later in the document

Repeated text was removed from page 69.

Reviewer #2:
Remarks to the Author:
The authors have done an excellent job of responding to my comments and criticisms. No further issues remain on my end.

We thank the reviewer for the positive response to our revised manuscript.

Reviewer #3:
Remarks to the Author:
This is a revision of the manuscript from Sahu et al. describing a large set of MPRA experiments. I appreciate the authors' efforts to improve the clarity and organization of the manuscript. I still think that there is a lot of material here for one paper, and that this is making the paper difficult to understand. However, the larger problem is that the data in the manuscript do not support the unusually broad conclusions.

We thank the reviewer for the feedback on our manuscript and for appreciating the improvements that were introduced in the previous revision. We apologize if the relationship between the results and the conclusions drawn from them was not communicated well enough. We have now carefully addressed each specific concern as detailed below. We have included more quantitative information about the specific analyses throughout the manuscript as also suggested by Reviewer#1, clarified and moderated statements that we agree on re-reading the manuscript reasonably could have been perceived as overly broad and qualitative. We hope that these modifications help to clarify the relationship between the results and the conclusions.

One claim the manuscript makes is that the study is "comprehensive", which implies that all of regulatory space has been assayed. The text makes a compelling argument that regulatory space is too big to be encompassed by sequences found in the genome, and that therefore, synthetic DNA sequences must be included. However, even though the DNA assayed here is "~100 times larger sequence space than the human genome" (line 22) when one considers "all combinations, orientations and spacings of the 1639 TFs in multiple independent sequence contexts" (lines 51-52) the sequence space assayed in this study still represents only a tiny fraction regulatory space, which undermines the claim of being comprehensive.

We thank the reviewer for the comment and agree that the word "comprehensive" was too strong. **We have now toned down the statements on page 3, line 58 and page 66, line 1605 and used more appropriate words.**

Another caveat related to the size of regulatory space is the claim that most TFs work independently and additively, without cooperative interactions. Since only a small fraction of regulatory space is being assayed, most combinations of TF sites, in most orientations and spacings, were not measured. Thus, the study is underpowered to detect interactions between TFs relative to the power to detect the additive contributions of TFs. No strong conclusions should be drawn from the lack of detected interactions: it is the expected result from a study with limited power to detect interactions.

We thank the reviewer for the comment and agree that the question of the statistical power to detect specific spacings and orientations is an important one. **We have now conducted a power analysis that simulates different effect sizes and motif match thresholds with the same data size as was used in the motif spacing analysis shown in the manuscript (Fig. 2c).** The analysis shows that we do have sufficient power to detect moderate effects (fold change ≥ 3 if 1% of the pairs had a specific interaction) with high probability (> 0.77) using the same motif match threshold as in the manuscript (10^{-4}) that is consistent with the interactions shown in the figure. Moreover, even with a more stringent threshold (6×10^{-5}) that corresponds approximately to a motif with information content of 15 bits typical for TFs, we would be able to detect fold changes ≥ 5 (probability > 0.92), but only p53/p63 motif interactions were detected with this threshold. Thus, although it is difficult to rule out that some specific interactions could have been missed, the power analysis indicates that if strong specific interactions existed between individually enriched motifs in the assay, we would have detected them with very high likelihood. **These aspects are now described in the Supplementary Note (page 3, lines 111-124) of the revised manuscript.**

Another example of a strong conclusion drawn from a negative result is on lines 95-97, where the authors invoke the sequential activity of TFs based on the lack of concordance between enhancer activities and in vitro binding data. The possibility that one or both data sets may contain significant errors is not considered.

We thank the reviewer for the comment and agree that the conclusions drawn from the weak correlation between motif activity and in vitro binding data were too strong. **We have now toned down the statement in the Results section of the revised manuscript (page 4, line 95-97), and**

also added further discussion to the Supplementary Note (page 2, lines 87-89) to acknowledge the limitations in comparing the results from two different assays.

The claim that only a small number of TFs are active in any cell type cannot be supported from assays in just two cell types, especially when both of the cell types derive from the endoderm.

We thank the reviewer for the comment. In the analysis shown in Fig. 2f, we have indeed used two cell lines of endodermal origin (GP5d and HepG2). However, the binary STARR-seq assays shown in Fig. 5a were performed from three cell lines, one of which (RPE1) is of ectodermal origin. Strong correlation observed in these three cell lines is consistent with our conclusion that the active motifs enriched within the regulatory elements are largely similar in different cell types. We however agree that further studies are needed for comprehensive understanding of regulatory activity in diverse cell types. **We have now clarified this issue (page 11, lines 266-267) and state in the discussion that "further analysis using main cell types representing all three germ layers is needed to determine whether and to what extent differentiated human cell types have retained the regulatory mechanisms that existed in their common unicellular ancestor" (page 17, lines 404-406). We also clarify in the Discussion that our result is consistent with our previous work showing that relatively few TFs show strong DNA binding activity in a cell, and that many of the strong binders are common to various cell types (Wei et al., 2018; PMID: 29786094; page 16, line 393-page 17, line 395). To further clarify the statements regarding the similarity of motif activities in different cell types, we have also now provided correlation coefficient values for Fig. 2f and Fig. 5a in the Results section of the revised manuscript (page 7, lines 164-165; page 11, lines 271-272).**

The claim that there are multiple categories of enhancers is only weakly supported and comes only categorizing active sequences by their epigenetic marks. Any randomly picked group of sequences could likewise be separated by the types of marks they carry, so the mere fact that enhancers have different marks does not prove that they function in qualitatively different ways from each other.

We apologize that the results regarding different enhancer classes were not explained clearly enough. **We have now modified the statement on page 9, lines 205-207, 211 to clarify that the classes were defined on the basis of the chromatin structure (ATAC-seq) and classical enhancer activity measured using a reporter assay (STARR-seq), and not only by enrichment of different epigenetic marks.** However, the enrichment for repressive histone marks was used for differentiating cryptic (silenced) enhancers from closed chromatin enhancers (both of these classes being STARR-seq+ and ATAC-seq- regions) and activating mark H3K27ac was correlated with activity of chromatin-dependent and classical enhancers. We feel that taking the epigenetic information into account is in line with how enhancer classification has been done previously (for example poised, primed, and active enhancers in Heinz et al., 2015, PMID: 25650801 were defined based on differential enrichment for H3K27me3, H3K4me1, and H3K27ac).

To further address the reviewer's concern, we also tested whether randomly picked genomic elements would show differential enrichment for regulatory features. For this, we applied a similar approach to that used for analyzing ATAC-seq peaks either overlapping or not overlapping with STARR-seq peaks (Fig. 3e in the manuscript, shown as panel on the left in **Editorial Figure 1**) for two randomized sets of ATAC-seq peaks (**Editorial Figure 1, *right***). This analysis showed that there were no differentially enriched features in the two randomized sets of ATAC-seq peaks, indicating that the differential features detected when the ATAC-seq peaks were classified on the basis of STARR-seq signal arise from genuine differences between the STARR-seq+ and STARR-seq- regions.

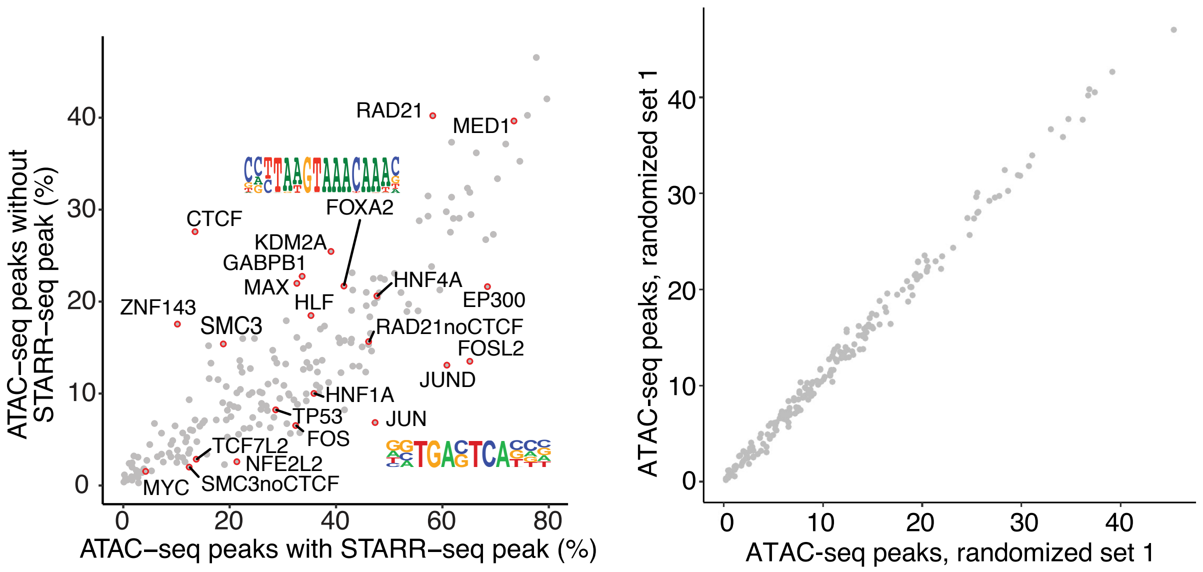

Editorial Figure 1. Left: Fig. 3e from the manuscript that shows comparison of ChIP-seq peaks within ATAC-seq peaks with (x-axis) and without (y-axis) STARR-seq peak in HepG2 cells. For each TF or other chromatin-associated protein, the percentage of respective ATAC-seq peaks overlapping with at least one ChIP-seq peak is shown. Right: Similar analysis as in left is applied to two randomized sets of ATAC-seq peaks generated by taking the peaks having the same size as the set overlapping the STARR-seq peaks (set1) and the rest of the peaks (set 2).

The TFs with the largest response in the experiments are p53 and IRF, two TFs that are part of a known artifact of Starr-seq that has to do with the cellular response to foreign DNA. The authors have done experiments to rule out that the cellular alarm system is not responsible for their measured activities of p53 and IRF, but it remains worrying that this is the largest effect in the data.

We thank the reviewer for the important comment. We agree that from the previous version of our manuscript it may have appeared that p53 and IRF would dominate the cellular regulatory landscape. We have now performed additional analysis and clarified in the text that although p53 and IRF were the most enriched motifs, the large majority of active promoter or enhancer elements peaks do not contain these elements. As shown in Fig. 2f, p53 family motifs are the strongest activators in HepG2 and GP5d cells, having the largest fold change of motif match count over input in each cell line. To determine what fraction of all detected elements are regulated by p53, we analyzed what proportion of the genomic STARR-seq peaks overlap with TP53 ChIP-seq peaks. This analysis revealed that p53 is not responsible for most of the enhancer activity detected using STARR-seq. Specifically, 16% of the genomic STARR-seq peaks overlap with p53 ChIP-seq peak in GP5d cells, whereas only 5.1% of the STARR-seq peaks harbor a p53 motif (Extended Data Fig. 9b). In HepG2 cells, 4.9% of the genomic STARR-seq peaks overlap with p53 ChIP-seq peaks, and only 5.4% of the STARR-seq peaks harbor a p53 motif (shown in **updated Extended Data Fig. 9d**). Similarly, only 3.0% of the GP5d STARR-seq peaks harbor an IRF3 motif (shown in **updated Extended Data Fig. 9b**). **These results are now described in page 8, lines 188-194 of the revised manuscript where we state that although p53 and IRF were the most enriched motifs, the large majority of active enhancers did not contain these elements. This suggests that gene regularly system of the cell is not dominated by these cellular alarms, which is further discussed in the Supplementary Note (page 6, line 293-page 7, line 304). Certainly, the extent to which different factors contribute to the enhancer activity in a cell is an interesting question for further studies that could be addressed for example by performing the STARR-seq assay after introducing mutations throughout the genome, which is now mentioned in the Discussion of the revised manuscript on page 17, lines 406-408.**

We again thank all the reviewers for their constructive comments and hope that the revised manuscript would be acceptable for publication in *Nature Genetics*.

naturerresearch

Final Decision Letter:

Our ref: NG-A57325R2

4th Oct 2021

Dear Jussi,

Thank you for submitting your revised manuscript entitled "Sequence determinants of human gene regulatory elements" (NG-A57325R2). My colleagues and I find that the paper has improved in revision, and therefore we'll be happy in principle to publish it in Nature Genetics, pending minor revisions to comply with our editorial and formatting guidelines.

We are now performing detailed checks on your paper and will send you a checklist detailing our editorial and formatting requirements soon. Please do not upload the final materials and make any revisions until you receive this additional information from us.

Thank you again for your interest in Nature Genetics. Please do not hesitate to contact me if you have any questions.

Congratulations!

Sincerely,

Tiago

Tiago Faial, PhD
Senior Editor
Nature Genetics
https://orcid.org/0000-0003-0864-1200
